# Author Correction: Associations between dimensions of behaviour, personality traits, and mental-health during the COVID-19 pandemic in the United Kingdom

**DOI:** 10.1038/s41467-021-25271-6

**Published:** 2021-08-16

**Authors:** Adam Hampshire, Peter J. Hellyer, Eyal Soreq, Mitul A. Mehta, Konstantinos Ioannidis, William Trender, Jon E. Grant, Samuel R. Chamberlain

**Affiliations:** 1 Imperial College London, London, UK; 2 King’s College London, London, UK; 3 Cambridgeshire and Peterborough NHS Foundation Trust, Cambridge, UK; 4 Department of Psychiatry, University of Cambridge, Cambridge, UK; 5 Department of Psychiatry, University of Chicago, Chicago, IL USA; 6 Department of Psychiatry, University of Southampton, Southampton, UK; 7 Southern Health NHS Foundation Trust, Southampton, UK

**Keywords:** Human behaviour, Medical research

Correction to: *Nature Communications* 10.1038/s41467-021-24365-5, published online 16 July 2021.

The original version of this Article contained an error in Fig. 2, in which graphs shown under the headings “Feeling tired or having little energy”, “Trouble concentrating on things, such as reading the newspaper or watching television” and “Not being able to get to sleep or stay asleep?” were inadvertently duplicated from the graph under the heading “Feeling down or depressed” during the preparation of the files.

The correct version of Fig. 2 is:
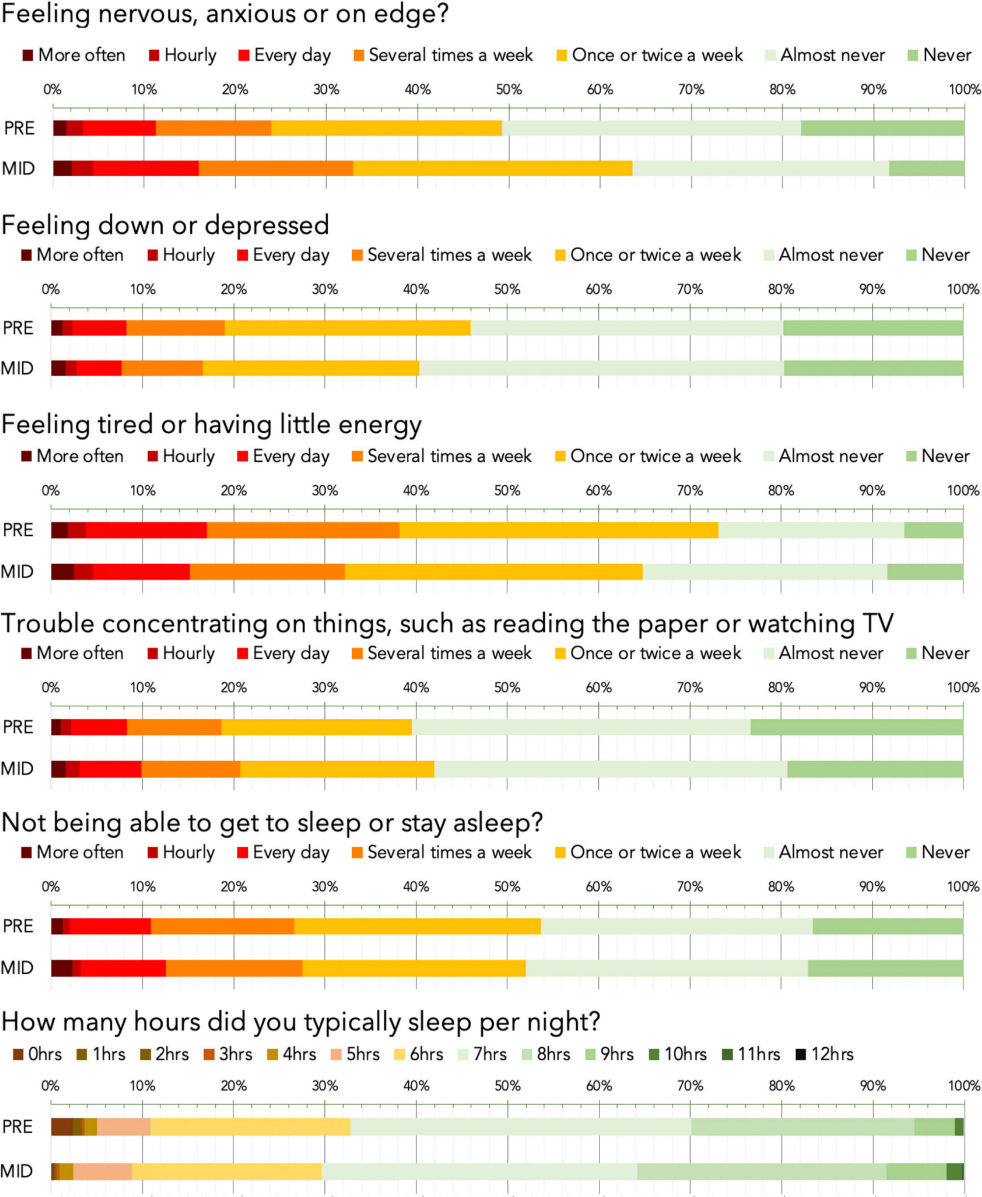


which replaces the previous incorrect version:
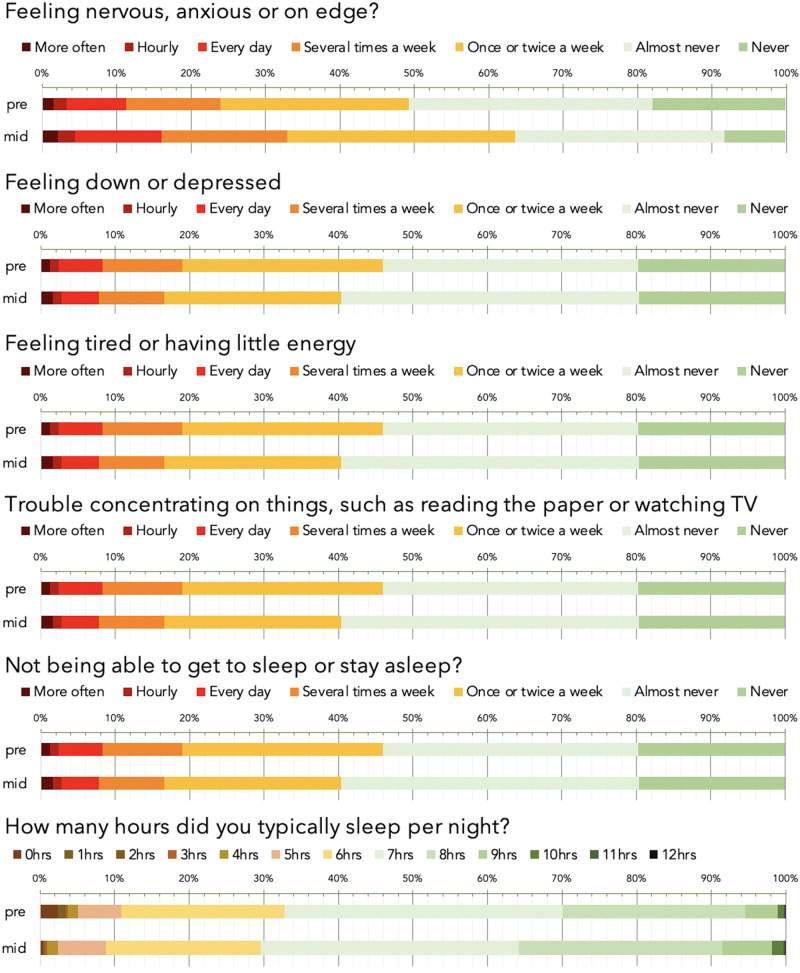


This has been corrected in both the PDF and HTML versions of the Article.

The original version of the Source Data file associated with this Article included errors in the data underlying Fig. 2, in which the data listed for the categories “Feeling tired or having little energy”, “Trouble concentrating on things, such as reading the newspaper or watching television” and “Not being able to get to sleep or stay asleep?” were inadvertently duplicated from the data under heading “feeling down or depressed” during preparation of the files.

The HTML has been updated to include a corrected version of the Source Data file. The original incorrect version of Source Data (Supplementary Information 1) and the correct version of Source Data (Supplementary Information 2) can be found as Supplementary Information associated with this correction.

## Supplementary information

Supplementary Information 1

Supplementary Information 2

