# Supplementary material for: Associations between dimensions of behaviour, personality traits, and mental-health during the COVID-19 pandemic in the United Kingdom
Source: Nat Commun. Author manuscript; Available in PMC 2021 Jul 20. (PMC8285408; doi:10.1038/s41467-021-24365-5)
Supplement: Supplementary Information [file EMS127615-supplement-Supplementary_Information.docx]

**Supplementary information: Hampshire et al.**

**Contents**

**Pages 2-4 Supplementary methods** - Study Questionnaires

**Pages 5-8 Supplementary figures 1-8 & Supplementary table 1** - Comparison of demographic distributions in the pre-, early- and mid-pandemic datasets

**Page 9 Supplementary figure 9** – Analysis of day-by-day differences in mood self-assessment scores in January and May

**Pages 10-16 Supplementary tables 2-16** – Relationship between standard mental health score differences pre- to mid- pandemic lockdown and population factors

**Page 17 Supplementary results 1** - PD-GIS mental health sample bias analysis

**Page 17 Supplementary figure 10** - PD-GIS Principal Component Analysis

**Page 18 Supplementary figure 11** - PD-GIS vs Mental Health CCA train-test and sub sampling analysis

**Page 19-34 Supplementary tables 17-31** - Predicting individual differences in PD-GIS component scores from population variables

**Pages 35-44 Supplementary tables 32-41 & supplementary figures 12-16** - Principal Component Analyses of questionnaire scales.

**Page 35 Figure - data key**

**Supplementary methods - Study Questionnaires**

**(a) Demographic and other contextual information**

The following background information was collected from participants: age, gender, ethnicity, country of residence, level of education, occupational status, and income.

**(b) Mood, anxiety, and sleep**

Mood and anxiety symptoms were recorded using items from the extensively validated Patient Health Questionnaire 2 (PHQ-2) and GAD-7 respectively ^1,2^The PHQ-2 and GAD-7 ask about symptoms over the preceding two weeks, and each question is answered on a 4-point scale, from 0 (not at all) to 3 (nearly every day). Additionally, we asked how many hours on average participants slept per night.

**(c) Personality traits, and compulsivity**

Personality traits were quantified using the extensively validated Big-5 Inventory, which comprises 44 questions^3^. Each question is a short phrase and is answered on a 5-point rating scale from 1 (strongly disagree) to 5 (strongly agree). Aspects of personality classically reflect extraversion, agreeableness, conscientiousness, neuroticism and openness to experience ^3^Based on prior factor analysis of data from 60,000 participants, we used an abbreviated version, comprising 18 questions with a data-driven structure of 6 components. These are reported in the factor analysis in **Appendix 2**.

Compulsivity is a trans-diagnostic concept representing the tendency towards repetitive habits, and was measured using the Cambridge-Chicago Compulsivity Trait Scale (CHI-T)^4^. This is a 15-item questionnaire that is answered on a 4-point rating scale ranging from 1 (strongly disagree) to 4 (strongly agree). The CHI-T is sensitive to compulsivity across a range of disorders ^4,5^.

**(d) Impact of the pandemic**

The Pandemic General Impact Scale (PD-GIS) was developed specifically for the current study to quantify the self-perceived negative and positive impacts of the COVID-19 pandemic on daily life, as well as outlook, on multiple levels of psycho-socio-economic investigation. These pandemic-oriented measures can be juxtaposed against more generic scales, e.g., measuring of mental health symptoms. The PD-GIS was generated in response to the need for a scale that captured aspects of how people considered their daily lives to have been affected, that is, as opposed to more generic measures of their mental health status. The items were generated by the authors of this article, comprising psychiatrists, psychologists and neuroscientists. At a coarse grain, it was designed to have three main sub-sections. (1) Aspects of positive impact. (2) Aspects of negative impact. (3) Outlook. The exact wording of the scale was refined through multiple iterations by the researchers, who are experienced in developing new scales, with feedback from producers at BBC and BBC media, who are knowledgeable regarding the wording of questions such that people will be comfortable answering them online. Prior to application in the main study, the preliminary scale was deployed in ~1,000 participants as a pilot. On analysing the pilot data it was observed that there appeared to be multiple dimensions to the PD-GIS latent variable structure, that these were quite independent within the positive and negative domains, and that the item-component loadings were relatively simple and readily interpretable. Therefore, the full scale study included the scale as originally designed, so no items were removed.

The instrument comprises 47 questions, relating to potential negative and positive aspects of the situation, and longer-term outlook. Each item is answered on a 5-point scale ranging from 1 (strongly disagree) to 4 (strongly agree). Negative and positive impact items are generally couched in terms of how things have changed due to the pandemic and are contextualised by 'Please indicate how well the following statements describe the impact of the pandemic on you.' Negative impact questions were designed to cover areas of concern for health (own health and that of others), being concerned with the consequences of contracting COVID-19, loneliness, conflict at home, negative emotions from reading or listening to news, grieving, loss of employment, job or income, loss of leisure and wellbeing activities, loss of daily structure, disruption of sleep patterns, less healthy lifestyles, less focus on personal hygiene, loss of productivity, social disconnection, life being dominated by infection control routines, loss of important goods, medication or services, more arguments in the household, and going on the internet to avoid people at home. Positive impact questions were designed to cover less commuting time, more structure to the day, joy at being able to spent more time with people at home, more connections with people online, sense of shared community, more efficient or productive work, being more relaxed due to more time at home, better sleep due to spare time, greater sense of purpose in work, greater opportunity to exercise, improved natural environment, time to read for pleasure, work less stressful due to doing it from home, spending more time on hobbies, spending less and saving money, more social contact outside of the home, feeling less tired, feeling better connected with people at home, more wildlife, taking greater appreciation for the simple things in life, and being less stressed by daily responsibilities.

Full-text for the PD-GIS is as follows:

*Cue- Please indicate how well the following statements describe the impact of lockdown on you*

I am more concerned about my personal health

I am more concerned about the health of my loved ones

I feel more lonely than before

There is an increased frequency or intensity of conflict at home

I am preoccupied with consequences of getting COVID-19

Watching or reading the news brings on unpleasant emotions, or distressing thoughts that are hard to get rid of

I have been grieving due to the loss of someone close to me

I have lost employment, job opportunities or income

I have lost leisure opportunities or activities important for my well-being

I feel that my daily routine no longer has enough structure

I have experienced changes in my sleep/wake patterns

My lifestyle and/or daily routine has become more unhealthy

I have not paid as much attention to my personal hygiene

My productivity has gone down

I have felt disconnected from important people in my life

Infection control routines more than ever dominate my life

I have lost access to essential goods, services or medication

I am arguing more often with the people I live with

I go online more to avoid the people I live with

I feel that I have more time as am commuting less

There is now more structure to my day

I am happier as am able to spend more time with people within my home

I am connecting online with people who I had trouble finding the time for before

There is a greater sense of shared community

I am working more efficiently/productively now

I feel more relaxed as am spending more time at home

I am sleeping better as have more spare time

I feel a greater sense of purpose in the work that I do

I am able to exercise more often

When I go outside, the environment is quieter and more relaxing than it was before

I have more time to read just for pleasure

I find work less stressful now that I am doing it from home

I am now spending more time on hobbies that I enjoy

I am spending less and saving more money than before

I have more social contact outside of my home

I feel less tired now

I am better connected now with the people I live with

There seems to be more wildlife now

I am enjoying the simple things in life more

I feel less stressed by my daily responsibilities

*Cue - How will things change in the long term?*

I believe the world will be a better place than it was

I believe the negative impact on the economy will be short lived

Things will change but not necessarily for the worse

I have more belief that we can cope with global problems like climate change

Technology science and healthcare will improve more rapidly than before

**(e) Online Technology use**

Technology use was quantified by asking about frequency of use of the following, over the previous 4 week period: Smart Phone, Computer (Desktop or Laptop), Tablet Device, Gaming Console, Email, Social Media, reading the news, playing computer games, online gambling, working, learning/studying, shopping, streaming films or music, and searching for information online. Each question was responded to on a 7-point scale, from 0 (never) to 7 (more often than hourly every day).

**(f) Stress from online technology**

Stress from online technology was measured by asking the participants the following questions, regarding the past 4 weeks: When you checked Email, did it tend to make you feel stressed/unhappy or relieved/happy? When you used social media, did it tend to make you feel stressed/unhappy or relieved/happy? When you read the news, did it tend to make you feel stressed/unhappy or relieved/happy? When you played computer games, did it tend to make you feel stressed/unhappy or relieved/happy? The response options for each question were: "Mostly stressed/unhappy", "Mostly relieved/happy", "Both", or "Neither".

**(g) Maladaptive (‘Addictive’) use of online technology**

Maladaptive use of online technology was quantified using the following questions, which were based on expert consensus amongst the study team in the field of Problematic Usage of the Internet: How often did you check email or social media accounts after you went to bed? How often did you use internet related activities to block out disturbing thoughts or soothe yourself? How often did you choose to spend time on internet related activities to battle loneliness or boredom? How often did you suffer from negative financial consequences because of an online activity? How often did you check your email or social media account or equivalent before something else that you needed to do? How often did you try to stop an excessive online activity but feel a compulsion to continue? How often did you try to cut down the amount of time you spend on-line and fail? The questions asked about these areas over the preceding 4-week period. For the first question (using technology before bed), response options were 1 (never) to 5 (daily). For the other questions, response options were: 1 (never) to 7 (more than hourly every day).

**Comparison of demographic distributions in the pre, early and mid-pandemic datasets Proportions of participants in final analyses by population factor (Source data are provided as a Source Data file).**

**Supplementary figure 1. Age sample probability distributions**

**
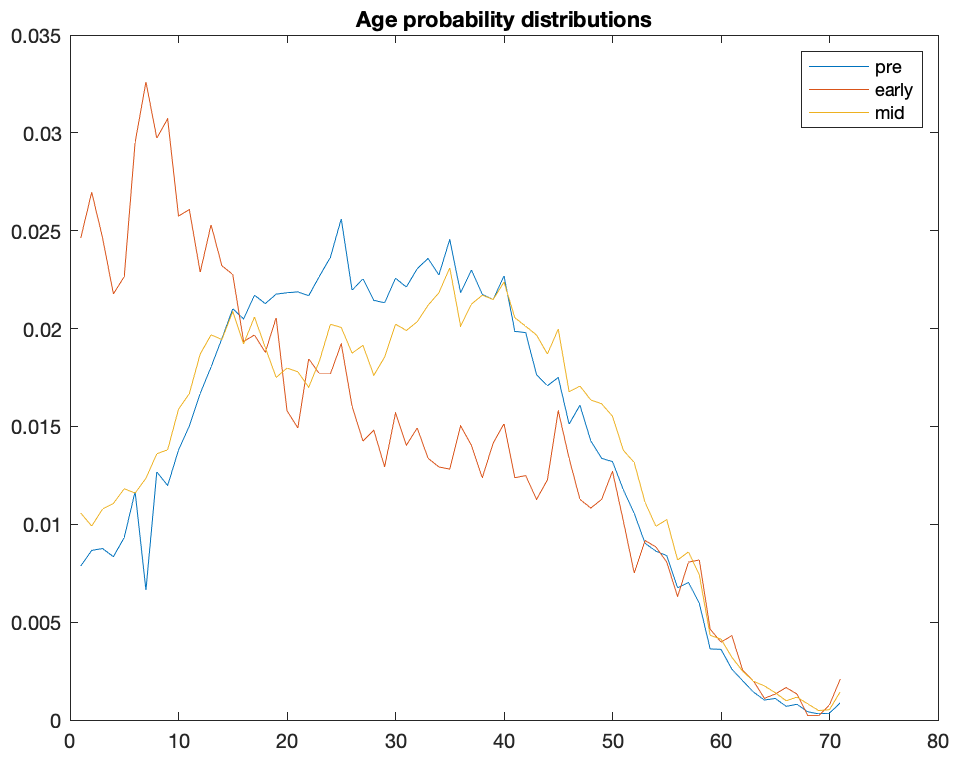
**

**Proportion of participants per age year within the pre-, early- and mid-pandemic epochs. X axis is age, Y axis is proportion per epoch.**

**Supplementary figure 2. Sex sample probability distributions**

**
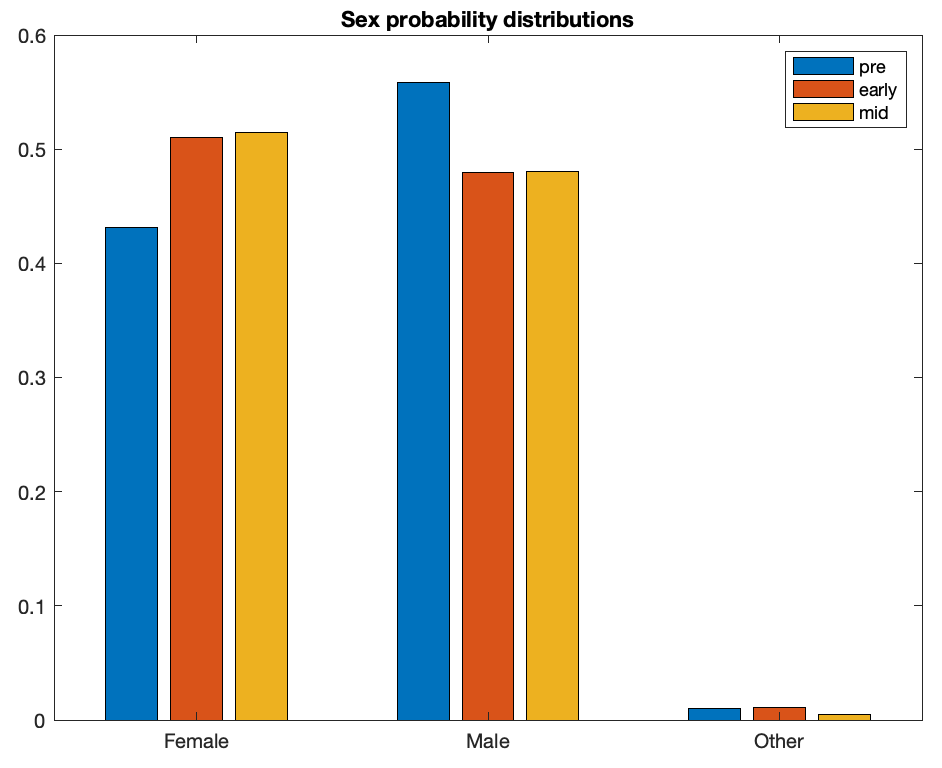
**

**Proportion of participants per sex group (male, female or other) within the pre-, early- and mid-pandemic epochs. Y axis is proportion per epoch.**

**Supplementary figure 3. Handedness sample probability distributions**

**
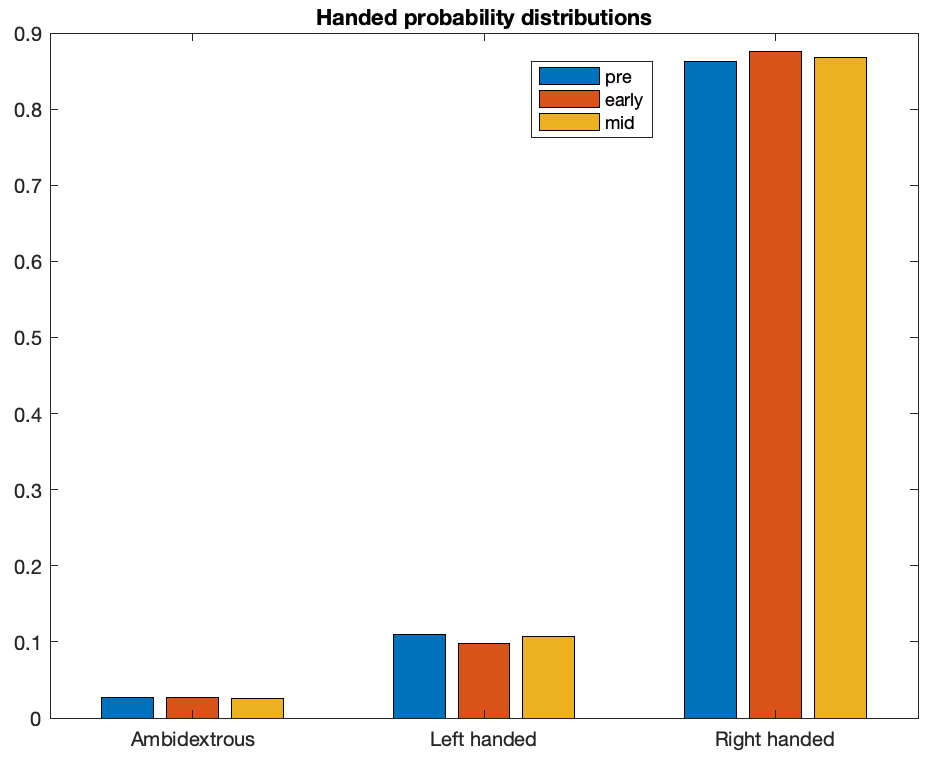
**

**Proportion of participants per handedness group within the pre-, early- and mid-pandemic epochs. Y axis is proportion per epoch.**

**Supplementary figure 4. Education level sample probability distributions**

**
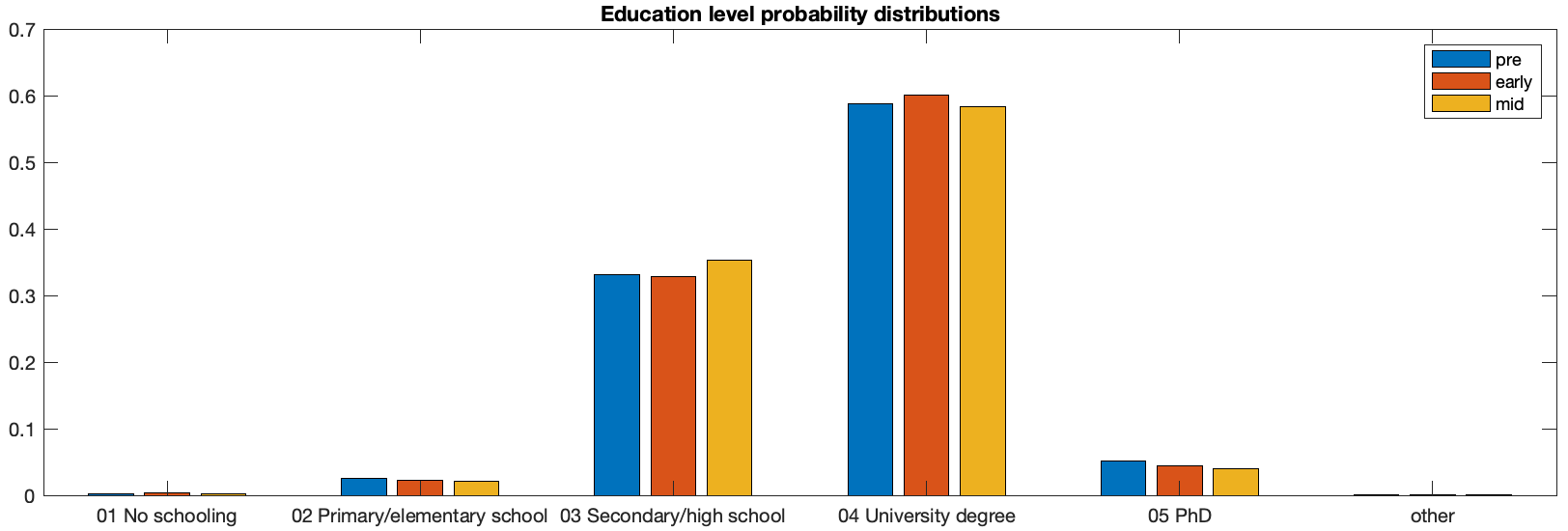
**

**Proportion of participants per education level within the pre-, early- and mid-pandemic epochs. Y axis is proportion per epoch.**

**Supplementary figure 5.** **Occupational status sample probability distributions**

**
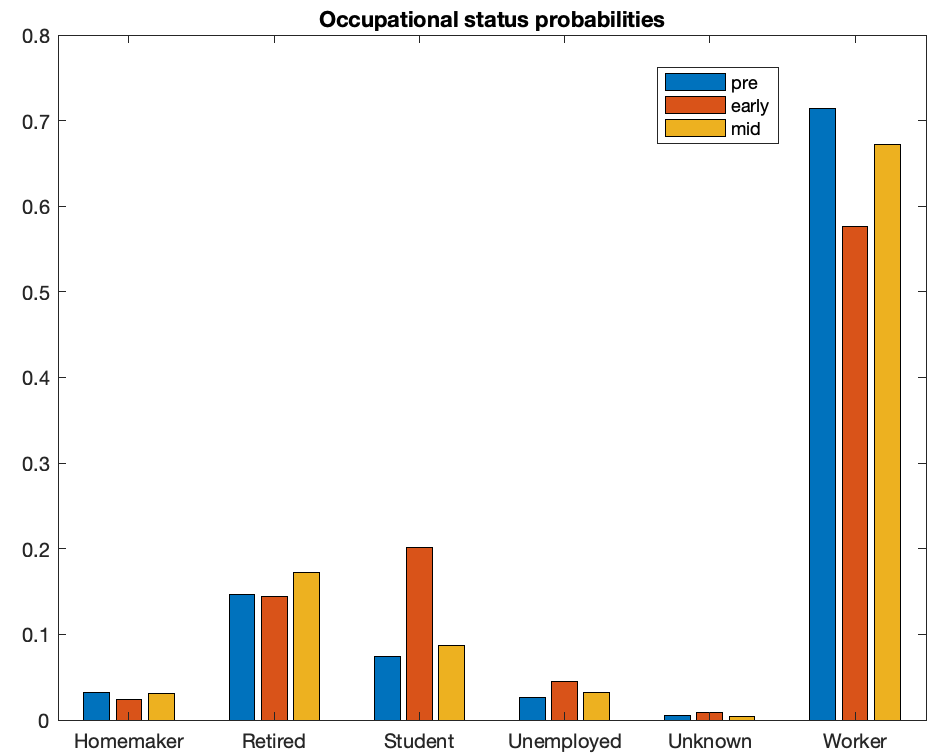
**

**Proportion of participants per occupational status group within the pre-, early- and mid-pandemic epochs. Y axis is proportion per epoch.**

**Supplementary figure 6. First language sample probability distributions**

**
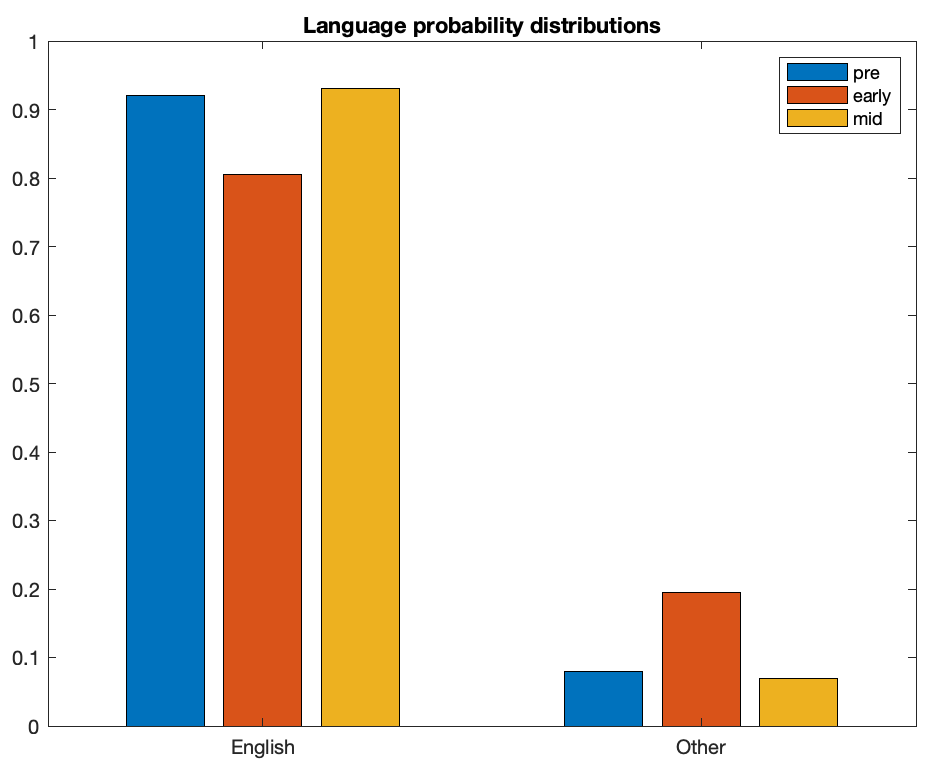
**

**Proportion of participants per first language within the pre-, early- and mid-pandemic epochs. Y axis is proportion per epoch.**

**Supplementary figure 7 Country of residence sample probability distributions**

**
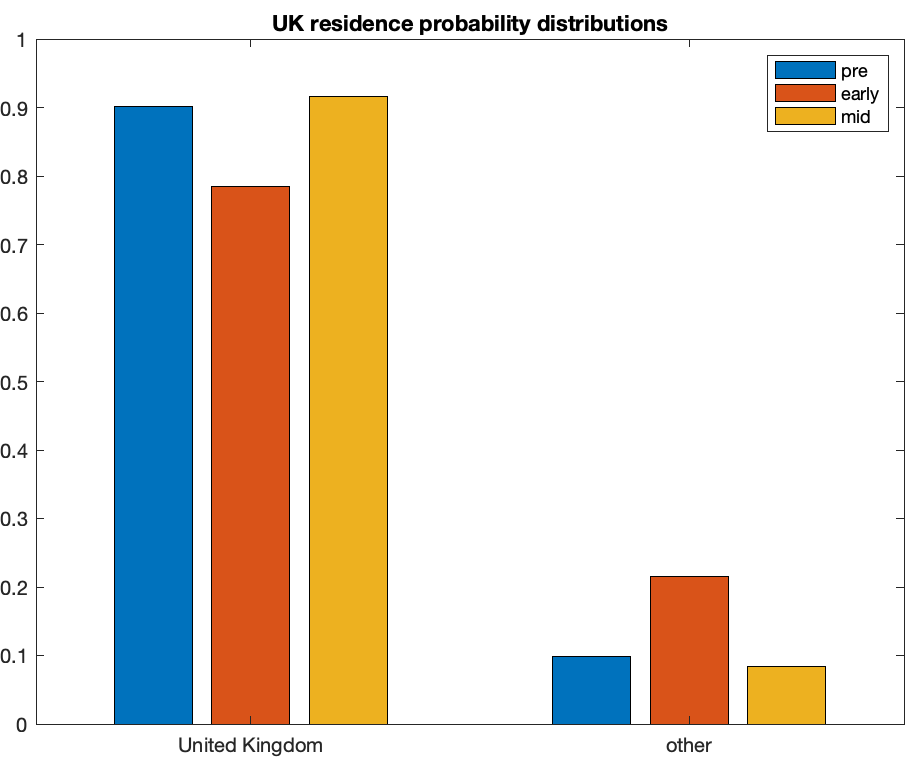
**

**Proportion of participants per country of residence within the pre-, early- and mid-pandemic epochs. Y axis is proportion per epoch.**

**Supplementary figure 8 Earnings sample probability distributions**

**
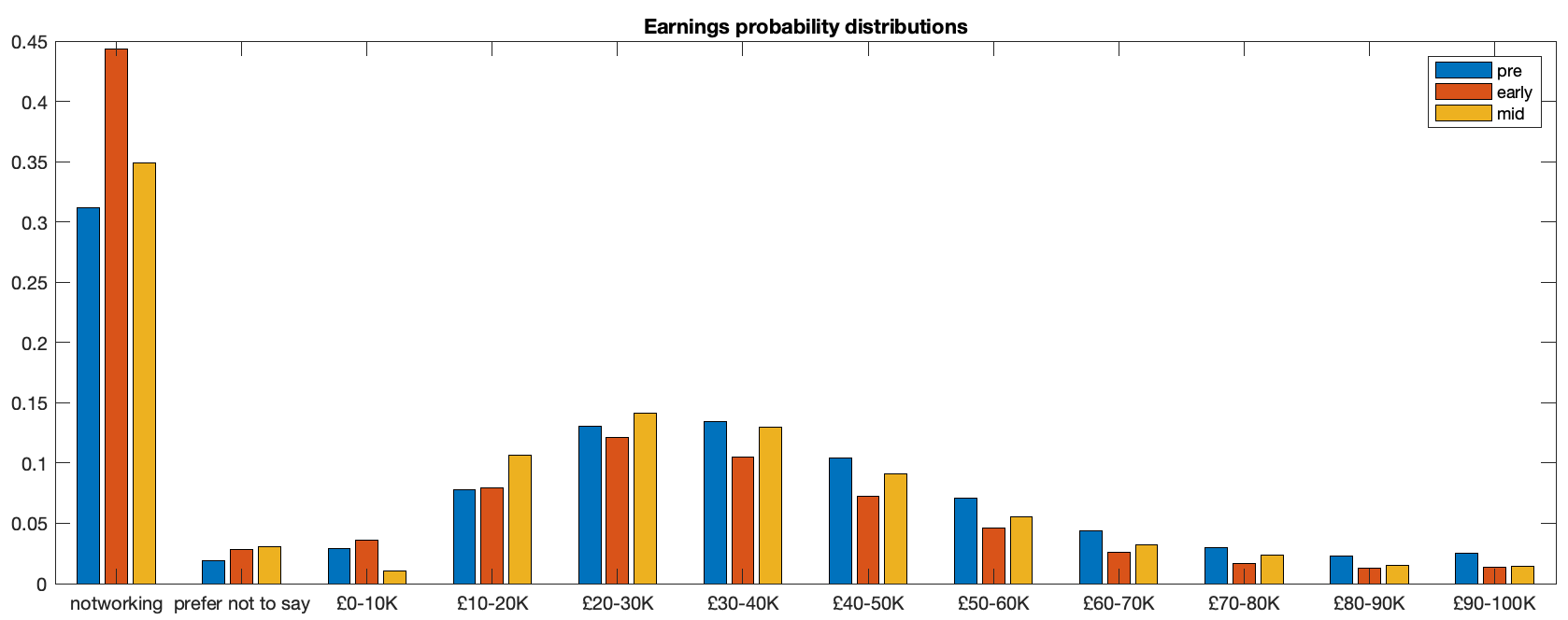
**

**Proportion of participants per earnings bracket within the pre-, early- and mid-pandemic epochs. Y axis is proportion per epoch.**

**Supplementary table 1 Sample probability distributions for ethnicity within the pre-, early- and mid-pandemic epochs**

|  | | |  |
| --- | --- | --- | --- |
| Pre | Early | Mid |  |
| 0.838 | 0.759 | 0.897 | White European or North American |
| 0.045 | 0.102 | 0.014 | East Asian |
| 0.037 | 0.039 | 0.035 | Indian, South Asian or South-East Asian |
| 0.035 | 0.026 | 0.006 | American Hispanic |
| 0.023 | 0.034 | 0.025 | Mixed ethnicity |
| 0.011 | 0.017 | 0.015 | Unknown/other |
| 0.006 | 0.009 | 0.004 | Sub-Saharan African or Afro-American |
| 0.004 | 0.013 | 0.004 | West-Central Asian |
| 0.003 | 0.003 | 0.002 | North African |

**Supplementary figure 9 – Analysis of day-by-day mood self-assessment scores in January and May**

Mean scores for mood self-assessment measures were contrasted separately for each of 31 days after the two promotion launches on January 1^st^ (blue, pre-pandemic) and May 2^nd^ (orange, mid-lockdown). Demographic variables including age, sex, handedness, ethnicity, first language, country of residence, education level, employment status and earning have been factored out. Y axis is in standard deviation units. X axis is days since launch. Shading represents the standard error of the mean for data collected on that day. The overall pattern of differences can be seen to be consistent throughout these two months with increased anxiety, increased sleep, reduced tiredness, and similar mean levels of depression, insomnia and problems concentrating. Therefore, the observed differences in mental health measures reflect sustained differences throughout these epochs, that is, as opposed to transient spikes in national mood on individual days. Source data are provided as a Source Data file.

**Relationship of mental health score differences pre- to mid- pandemic lockdown with population factors**

**Supplementary table 2 Anxiety – statistical significance of association between pre to mid pandemic difference with population variables**

|  | **DF** | **F** | p |
| --- | --- | --- | --- |
| **age** | 1 | 8683.70 | <0.0001 |
| **sex** | 2 | 1943.50 | <0.0001 |
| **handedness** | 2 | 3.52 | 0.0295 |
| **first language** | 1 | 45.67 | <0.0001 |
| **ethnic group** | 9 | 26.87 | <0.0001 |
| **country of residence** | 1 | 18.29 | <0.0001 |
| **education** | 5 | 44.82 | <0.0001 |
| **occupational status** | 5 | 701.29 | <0.0001 |
| **earnings** | 12 | 97.43 | <0.0001 |
| **epoch** | 1 | 7172.50 | <0.0001 |
| **age * epoch** | 1 | 75.94 | <0.0001 |
| **sex * epoch** | 2 | 152.25 | <0.0001 |
| **handedness * epoch** | 2 | 3.18 | 0.0415 |
| **first language * epoch** | 1 | 1.85 | 0.1732 |
| **ethnic group * epoch** | 9 | 4.80 | <0.0001 |
| **country of residence * epoch** | 1 | 4.34 | 0.0372 |
| **education * epoch** | 5 | 11.10 | <0.0001 |
| **occupational status * epoch** | 5 | 24.80 | <0.0001 |
| **earnings * epoch** | 11 | 4.14 | <0.0001 |
| **Error** | 3.442E+05 |  |  |

**Supplementary table 3 Depression – statistical significance of association between pre to mid pandemic difference with population variables**

|  | **DF** | **F** | p |
| --- | --- | --- | --- |
| **age** | 1 | 6975.91 | <0.0001 |
| **sex** | 2 | 451.96 | <0.0001 |
| **handedness** | 2 | 0.89 | 0.4087 |
| **first language** | 1 | 9.57 | 0.0020 |
| **ethnic group** | 9 | 20.84 | <0.0001 |
| **country of residence** | 1 | 11.86 | 0.0006 |
| **education** | 5 | 4.42 | 0.0005 |
| **occupational status** | 5 | 982.65 | <0.0001 |
| **earnings** | 12 | 217.32 | <0.0001 |
| **epoch** | 1 | 641.03 | <0.0001 |
| **age * epoch** | 1 | 12.23 | 0.0005 |
| **sex * epoch** | 2 | 3.26 | 0.0385 |
| **handedness * epoch** | 2 | 0.45 | 0.6370 |
| **first language * epoch** | 1 | 2.33 | 0.1271 |
| **ethnic group * epoch** | 9 | 4.33 | <0.0001 |
| **country of residence * epoch** | 1 | 4.90 | 0.0269 |
| **education * epoch** | 5 | 5.00 | 0.0001 |
| **occupational status * epoch** | 5 | 13.30 | <0.0001 |
| **earnings * epoch** | 11 | 3.19 | 0.0002 |
| **Error** | 3.44E+05 |  |  |

**Supplementary table 4 Tiredness – statistical significance of association between pre to mid pandemic difference with population variables**

|  | **DF** | **F** | p |
| --- | --- | --- | --- |
| **age** | 1 | 6828.41 | <0.0001 |
| **sex** | 2 | 1436.80 | <0.0001 |
| **handedness** | 2 | 3.68 | 0.0253 |
| **first language** | 1 | 233.96 | <0.0001 |
| **ethnic group** | 9 | 18.71 | <0.0001 |
| **country of residence** | 1 | 8.79 | 0.0030 |
| **education** | 5 | 39.21 | <0.0001 |
| **occupational status** | 5 | 1014.74 | <0.0001 |
| **earnings** | 12 | 121.64 | <0.0001 |
| **epoch** | 1 | 2193.40 | <0.0001 |
| **age * epoch** | 1 | 0.24 | 0.6271 |
| **sex * epoch** | 2 | 15.13 | <0.0001 |
| **handedness * epoch** | 2 | 3.42 | 0.0328 |
| **first language * epoch** | 1 | 0.72 | 0.3962 |
| **ethnic group * epoch** | 9 | 2.84 | 0.0024 |
| **country of residence * epoch** | 1 | 0.00 | 0.9528 |
| **education * epoch** | 5 | 4.08 | 0.0010 |
| **occupational status * epoch** | 5 | 2.72 | 0.0184 |
| **earnings * epoch** | 11 | 3.72 | <0.0001 |
| **Error** | 3.44E+05 |  |  |

**Supplementary table 5 Trouble concentrating – statistical significance of association between pre to mid pandemic difference with population variables**

|  | **DF** | **F** | p |
| --- | --- | --- | --- |
| **age** | 1 | 8034.33 | <0.0001 |
| **sex** | 2 | 145.74 | <0.0001 |
| **handedness** | 2 | 2.05 | 0.1290 |
| **first language** | 1 | 30.00 | <0.0001 |
| **ethnic group** | 9 | 13.63 | <0.0001 |
| **country of residence** | 1 | 0.28 | 0.5993 |
| **education** | 5 | 5.80 | <0.0001 |
| **occupational status** | 5 | 707.83 | <0.0001 |
| **earnings** | 12 | 81.65 | <0.0001 |
| **epoch** | 1 | 550.19 | <0.0001 |
| **age * epoch** | 1 | 2.34 | 0.1259 |
| **sex * epoch** | 2 | 3.92 | 0.0199 |
| **handedness * epoch** | 2 | 1.40 | 0.2456 |
| **first language * epoch** | 1 | 2.16 | 0.1412 |
| **ethnic group * epoch** | 9 | 5.18 | <0.0001 |
| **country of residence * epoch** | 1 | 0.36 | 0.5476 |
| **education * epoch** | 5 | 4.14 | 0.0009 |
| **occupational status * epoch** | 5 | 10.83 | <0.0001 |
| **earnings * epoch** | 11 | 1.54 | 0.1097 |
| **Error** | 3.44E+05 |  |  |

**Supplementary table 6 Insomnia – statistical significance of association between pre to mid pandemic difference with population variables**

|  | **DF** | **F** | p |
| --- | --- | --- | --- |
| **age** | 1 | 395.76 | <0.0001 |
| **sex** | 2 | 885.26 | <0.0001 |
| **handedness** | 2 | 6.36 | 0.0017 |
| **first language** | 1 | 471.69 | <0.0001 |
| **ethnic group** | 9 | 14.87 | <0.0001 |
| **country of residence** | 1 | 4.41 | 0.0357 |
| **education** | 5 | 74.51 | <0.0001 |
| **occupational status** | 5 | 521.86 | <0.0001 |
| **earnings** | 12 | 63.87 | <0.0001 |
| **epoch** | 1 | 18.22 | <0.0001 |
| **age * epoch** | 1 | 24.19 | <0.0001 |
| **sex * epoch** | 2 | 0.36 | 0.6948 |
| **handedness * epoch** | 2 | 1.71 | 0.1817 |
| **first language * epoch** | 1 | 9.24 | 0.0024 |
| **ethnic group * epoch** | 9 | 2.06 | 0.0294 |
| **country of residence * epoch** | 1 | 2.37 | 0.1240 |
| **education * epoch** | 5 | 2.39 | 0.0356 |
| **occupational status * epoch** | 5 | 2.27 | 0.0449 |
| **earnings * epoch** | 11 | 2.66 | 0.0020 |
| **Error** | 3.44E+05 |  |  |

**Supplementary table 7 Hours slept – statistical significance of association between pre to mid pandemic difference with population variables**

|  | **DF** | **F** | p |
| --- | --- | --- | --- |
| **age** | 1 | 3985.93 | <0.0001 |
| **sex** | 2 | 39.91 | <0.0001 |
| **handedness** | 2 | 38.85 | <0.0001 |
| **first language** | 1 | 0.63 | 0.4273 |
| **ethnic group** | 9 | 75.53 | <0.0001 |
| **country of residence** | 1 | 11.35 | 0.0008 |
| **education** | 5 | 213.37 | <0.0001 |
| **occupational status** | 5 | 215.77 | <0.0001 |
| **earnings** | 12 | 9.45 | <0.0001 |
| **epoch** | 1 | 1397.34 | <0.0001 |
| **age * epoch** | 1 | 197.47 | <0.0001 |
| **sex * epoch** | 2 | 0.33 | 0.7163 |
| **handedness * epoch** | 2 | 0.17 | 0.8431 |
| **first language * epoch** | 1 | 14.97 | 0.0001 |
| **ethnic group * epoch** | 9 | 3.07 | 0.0011 |
| **country of residence * epoch** | 1 | 8.69 | 0.0032 |
| **education * epoch** | 5 | 0.76 | 0.5782 |
| **occupational status * epoch** | 5 | 20.49 | <0.0001 |
| **earnings * epoch** | 11 | 1.12 | 0.3438 |
| **Error** | 3.44E+05 |  |  |

**Supplementary table 8 Differences in mean mid-pandemic minus pre-pandemic mental health scores by age (SD units)**

| **AGE** | **Anxiety** | **Depression** | **Tiredness** | **Problems concentrating** | **Insomnia** | **Hours Slept** |
| --- | --- | --- | --- | --- | --- | --- |
| **16** | 0.09 | -0.17 | -0.15 | 0.03 | 0.07 | 0.49 |
| **17** | 0.17 | -0.05 | -0.03 | 0.17 | 0.15 | 0.41 |
| **18** | 0.10 | -0.14 | -0.16 | 0.03 | 0.07 | 0.44 |
| **19** | 0.10 | -0.14 | -0.09 | 0.06 | -0.03 | 0.42 |
| **20** | 0.15 | -0.15 | -0.15 | 0.05 | -0.02 | 0.26 |
| **21** | 0.13 | -0.17 | -0.14 | 0.04 | 0.01 | 0.29 |
| **22** | 0.22 | -0.10 | -0.06 | 0.08 | 0.03 | 0.36 |
| **23** | 0.24 | -0.12 | -0.09 | 0.08 | 0.08 | 0.21 |
| **24** | 0.20 | -0.15 | -0.17 | 0.06 | -0.02 | 0.28 |
| **25** | 0.22 | -0.09 | -0.13 | 0.08 | 0.04 | 0.25 |
| **26** | 0.24 | -0.08 | -0.16 | 0.10 | 0.07 | 0.25 |
| **27** | 0.23 | -0.10 | -0.14 | 0.12 | 0.00 | 0.26 |
| **28** | 0.21 | -0.14 | -0.14 | 0.05 | -0.01 | 0.30 |
| **29** | 0.23 | -0.10 | -0.13 | 0.09 | 0.06 | 0.19 |
| **30** | 0.23 | -0.12 | -0.17 | 0.06 | 0.00 | 0.21 |
| **31** | 0.26 | -0.11 | -0.17 | 0.08 | 0.04 | 0.21 |
| **32** | 0.28 | -0.10 | -0.17 | 0.08 | 0.00 | 0.18 |
| **33** | 0.30 | -0.07 | -0.17 | 0.11 | 0.03 | 0.11 |
| **34** | 0.26 | -0.09 | -0.16 | 0.06 | -0.01 | 0.13 |
| **35** | 0.29 | -0.05 | -0.17 | 0.06 | 0.02 | 0.13 |
| **36** | 0.27 | -0.06 | -0.15 | 0.08 | 0.01 | 0.14 |
| **37** | 0.27 | -0.08 | -0.15 | 0.11 | -0.01 | 0.14 |
| **38** | 0.30 | -0.05 | -0.11 | 0.13 | 0.04 | 0.09 |
| **39** | 0.33 | -0.05 | -0.12 | 0.10 | 0.00 | 0.13 |
| **40** | 0.33 | -0.08 | -0.16 | 0.04 | 0.01 | 0.13 |
| **41** | 0.27 | -0.05 | -0.15 | 0.09 | 0.01 | 0.07 |
| **42** | 0.31 | -0.05 | -0.13 | 0.09 | 0.03 | 0.07 |
| **43** | 0.30 | -0.08 | -0.13 | 0.06 | -0.01 | 0.06 |
| **44** | 0.28 | -0.09 | -0.17 | 0.02 | -0.02 | 0.09 |
| **45** | 0.29 | -0.05 | -0.12 | 0.10 | -0.01 | 0.13 |
| **46** | 0.29 | -0.09 | -0.16 | 0.03 | -0.06 | 0.12 |
| **47** | 0.29 | -0.10 | -0.14 | 0.05 | -0.02 | 0.12 |
| **48** | 0.27 | -0.07 | -0.14 | 0.07 | -0.02 | 0.13 |
| **49** | 0.25 | -0.11 | -0.19 | 0.06 | -0.08 | 0.17 |
| **50** | 0.32 | -0.04 | -0.14 | 0.09 | 0.00 | 0.08 |
| **51** | 0.32 | -0.07 | -0.12 | 0.09 | -0.01 | 0.07 |
| **52** | 0.30 | -0.08 | -0.12 | 0.09 | 0.00 | 0.09 |
| **53** | 0.31 | -0.07 | -0.13 | 0.09 | 0.00 | 0.11 |
| **54** | 0.29 | -0.06 | -0.11 | 0.06 | 0.01 | 0.07 |
| **55** | 0.31 | -0.06 | -0.16 | 0.06 | 0.00 | 0.05 |
| **56** | 0.31 | -0.08 | -0.12 | 0.05 | -0.02 | 0.10 |
| **57** | 0.36 | -0.01 | -0.12 | 0.08 | -0.01 | 0.11 |
| **58** | 0.35 | -0.03 | -0.13 | 0.10 | 0.00 | 0.03 |
| **59** | 0.36 | -0.02 | -0.09 | 0.16 | 0.00 | 0.02 |
| **60** | 0.34 | -0.03 | -0.12 | 0.11 | 0.01 | 0.06 |
| **61** | 0.39 | -0.03 | -0.16 | 0.07 | 0.00 | 0.01 |
| **62** | 0.36 | -0.02 | -0.12 | 0.11 | -0.05 | 0.06 |
| **63** | 0.39 | 0.01 | -0.09 | 0.14 | 0.00 | 0.03 |
| **64** | 0.42 | 0.04 | -0.06 | 0.14 | 0.02 | -0.03 |
| **65** | 0.43 | 0.04 | -0.09 | 0.17 | 0.01 | 0.01 |
| **66** | 0.39 | 0.03 | -0.07 | 0.15 | -0.04 | -0.02 |
| **67** | 0.40 | 0.04 | -0.10 | 0.16 | -0.03 | -0.02 |
| **68** | 0.41 | 0.05 | -0.07 | 0.17 | -0.02 | -0.02 |
| **69** | 0.32 | -0.02 | -0.14 | 0.09 | -0.03 | 0.02 |
| **70** | 0.40 | 0.01 | -0.09 | 0.13 | -0.03 | -0.04 |
| **71** | 0.43 | 0.07 | -0.05 | 0.16 | 0.04 | -0.06 |
| **72** | 0.38 | 0.02 | -0.13 | 0.10 | 0.01 | -0.06 |
| **73** | 0.39 | 0.01 | -0.13 | 0.10 | -0.09 | 0.05 |
| **74** | 0.43 | 0.04 | -0.03 | 0.17 | 0.01 | -0.01 |
| **75** | 0.31 | -0.02 | -0.16 | 0.13 | -0.02 | 0.01 |
| **76** | 0.43 | 0.07 | -0.03 | 0.08 | 0.03 | -0.09 |
| **77** | 0.40 | 0.09 | -0.02 | 0.11 | 0.04 | -0.04 |
| **78** | 0.28 | -0.11 | -0.13 | 0.05 | -0.05 | -0.05 |
| **79** | 0.27 | -0.08 | 0.00 | 0.06 | 0.04 | -0.04 |
| **80** | 0.39 | 0.05 | -0.13 | 0.05 | 0.00 | -0.02 |
| **81** | 0.44 | 0.13 | -0.05 | 0.07 | -0.16 | 0.02 |
| **82** | 0.38 | 0.10 | -0.15 | 0.19 | -0.05 | 0.19 |
| **83** | 0.53 | 0.18 | -0.08 | 0.25 | 0.14 | -0.05 |
| **84** | 0.40 | 0.33 | 0.03 | 0.27 | -0.01 | -0.15 |
| **85** | 0.43 | 0.18 | -0.18 | 0.01 | -0.08 | 0.15 |
| **86** | 0.29 | -0.02 | -0.15 | -0.01 | -0.14 | 0.19 |

**Supplementary table 9 Differences in mean mid- minus pre-pandemic mental health scores by gender (SD units)**

| **AGE** | **Anxiety** | **Depression** | **Tiredness** | **Problems concentrating** | **Insomnia** | **Hours Slept** |
| --- | --- | --- | --- | --- | --- | --- |
| **Female** | 0.33 | -0.08 | -0.17 | 0.08 | 0.00 | 0.12 |
| **Male** | 0.22 | -0.07 | -0.14 | 0.08 | -0.01 | 0.14 |
| **Other** | 0.39 | 0.07 | -0.02 | 0.22 | 0.07 | 0.22 |

**Supplementary table 10 Differences in mean mid- minus pre-pandemic mental health scores by handedness (SD units)**

| **Handed** | **Anxiety** | **Depression** | **Tiredness** | **Problems concentrating** | **Insomnia** | **Hours Slept** |
| --- | --- | --- | --- | --- | --- | --- |
| **Ambidextrous** | 0.23 | -0.05 | -0.08 | 0.10 | 0.04 | 0.12 |
| **Left** | 0.30 | -0.07 | -0.14 | 0.07 | 0.00 | 0.14 |
| **Right** | 0.29 | -0.07 | -0.14 | 0.08 | 0.01 | 0.14 |

**Supplementary table 11 Differences in mean mid- minus pre-pandemic mental health scores by 1^st^ language (SD units)**

| **1st language** | **Anxiety** | **Depression** | **Tiredness** | **Problems concentrating** | **Insomnia** | **Hours Slept** |
| --- | --- | --- | --- | --- | --- | --- |
| **English** | 0.29 | -0.06 | -0.14 | 0.09 | 0.01 | 0.13 |
| **Other** | 0.23 | -0.13 | -0.17 | 0.03 | -0.03 | 0.21 |

**Supplementary table 12 Differences in mean mid- minus pre-pandemic mental health scores by ethnic group (SD units)**

|  | **Anxiety** | **Depression** | **Tiredness** | **Problems concentrating** | **Insomnia** | **Hours Slept** |
| --- | --- | --- | --- | --- | --- | --- |
| **American Hispanic** | 0.16 | -0.20 | -0.28 | 0.00 | -0.08 | 0.13 |
| **Mixed** | 0.22 | -0.07 | -0.09 | 0.06 | 0.00 | 0.19 |
| **African** | 0.19 | -0.13 | -0.15 | 0.03 | 0.03 | 0.29 |
| **Asian** | 0.21 | -0.09 | -0.18 | 0.06 | 0.02 | 0.18 |
| **Other/unknown** | 0.27 | -0.07 | -0.11 | 0.09 | 0.06 | 0.10 |
| **White** | 0.30 | -0.06 | -0.13 | 0.09 | 0.01 | 0.13 |

**Supplementary table 13 Differences in mean mid- minus pre-pandemic mental health scores by country of residence (SD units)**

|  | **Anxiety** | **Depression** | **Tiredness** | **Problems concentrating** | **Insomnia** | **Hours Slept** |
| --- | --- | --- | --- | --- | --- | --- |
| **UK** | 0.29 | -0.06 | -0.14 | 0.09 | 0.00 | 0.14 |
| **Other** | 0.24 | -0.11 | -0.14 | 0.05 | 0.03 | 0.09 |

**Supplementary table 14 Differences in mean mid- minus pre-pandemic mental health scores by education (SD units)**

|  | **Anxiety** | **Depression** | **Tiredness** | **Problems concentrating** | **Insomnia** | **Hours Slept** |
| --- | --- | --- | --- | --- | --- | --- |
| **No school** | 0.34 | 0.15 | 0.09 | 0.22 | 0.09 | 0.18 |
| **Primary/elementary** | 0.22 | -0.03 | -0.14 | 0.10 | 0.02 | 0.05 |
| **Secondary/high** | 0.26 | -0.05 | -0.13 | 0.08 | 0.02 | 0.12 |
| **Degree** | 0.31 | -0.07 | -0.15 | 0.08 | 0.00 | 0.15 |
| **PhD** | 0.31 | -0.09 | -0.13 | 0.10 | -0.01 | 0.12 |

**Supplementary table 15 Differences in mean mid- minus pre-pandemic mental health scores by occupational status (SD units)**

|  | **Anxiety** | **Depression** | **Tiredness** | **Problems concentrating** | **Insomnia** | **Hours Slept** |
| --- | --- | --- | --- | --- | --- | --- |
| **Disabled** | 0.01 | -0.12 | -0.06 | -0.06 | -0.07 | 0.00 |
| **Homemaker** | 0.31 | -0.07 | -0.13 | 0.05 | -0.02 | 0.02 |
| **Retired** | 0.38 | 0.02 | -0.09 | 0.13 | -0.02 | -0.01 |
| **Student** | 0.18 | -0.11 | -0.11 | 0.07 | 0.04 | 0.37 |
| **Unemployed** | 0.16 | -0.14 | -0.10 | 0.07 | -0.04 | 0.11 |
| **Worker** | 0.29 | -0.08 | -0.15 | 0.08 | 0.01 | 0.14 |

**Supplementary table 16 Differences in mean mid- minus pre-pandemic mental health scores by earnings (SD units)**

|  | **Anxiety** | **Depression** | **Tiredness** | **Problems concentrating** | **Insomnia** | **Hours Slept** |
| --- | --- | --- | --- | --- | --- | --- |
| **Not saying** | 0.38 | -0.01 | -0.04 | 0.09 | 0.08 | 0.15 |
| **Not working** | 0.28 | -0.05 | -0.11 | 0.09 | -0.01 | 0.11 |
| **£0-10K** | 0.32 | 0.01 | -0.10 | 0.13 | 0.00 | 0.19 |
| **£10-20K** | 0.26 | -0.10 | -0.17 | 0.06 | 0.03 | 0.12 |
| **£20-30K** | 0.27 | -0.09 | -0.16 | 0.07 | 0.01 | 0.16 |
| **£30-40K** | 0.27 | -0.10 | -0.18 | 0.07 | 0.00 | 0.14 |
| **£40-50K** | 0.26 | -0.12 | -0.20 | 0.05 | -0.03 | 0.15 |
| **£50-60K** | 0.26 | -0.12 | -0.20 | 0.05 | -0.03 | 0.15 |
| **£60-70K** | 0.29 | -0.10 | -0.18 | 0.04 | -0.03 | 0.12 |
| **£70-80K** | 0.29 | -0.10 | -0.15 | 0.07 | -0.06 | 0.11 |
| **£80-90K** | 0.28 | -0.11 | -0.13 | 0.06 | -0.06 | 0.16 |
| **£90-100K** | 0.29 | -0.09 | -0.16 | 0.07 | 0.01 | 0.08 |
| **>100K** | 0.32 | -0.06 | -0.15 | 0.11 | -0.01 | 0.14 |

**Supplementary results 1 – PD-GIS mental health sampling bias analysis**

One concern could be that people who opt to answer Cornonavirus-19 questionnaires are those for whom it is more relevant, e.g., due to their mental health status. To address this issue, we quantified sampling bias for the optional self-perceived impact sub-scale by analysing differences in mood measures for participants who did (79,736) minus did not (112046) opt to complete the PD-GIS. Differences in anxiety (0.018SDs t=2.0247 p=0.043), depression (-0.037SDs t=-4.4958 p<0.001), concentration (-0.073 SDs t=-8.4393 p<0.001), insomnia (0.032SDs t=3.4731 p<0.001), hours slept (-0.058SDs t=-7.1141 p<0.001) and tiredness (0.035SDs t=4.0017 p<0.001) scores were statistically significant but bi-directional with respect to valence, and critically, of negligible effect size scale. This accords poorly with the possibility of sampling bias towards people for whom mental health problems are most relevant during the pandemic in the context of PD-GIS analysis.

**Supplementary figure 10 – PCA analysis of the PD-GIS items.**

A MATLAB implementation of Horn’s Parallel Analysis^6^ was applied to estimate the number of significant components from the principal component analysis. PCA. This is a permutation-based approach whereby the true data are permuted and data reduced with PCA many times, producing distributions of variance explained by components at each index for statistical comparison to those observed for the unpermuted data. Estimated with 1000 permutations indicated 7 statistically significant components (greater than 95% of values within the corresponding null distribution) when the PD-GIS data were analysed in this manner. Application of the Kaiser convention would indicate 11 components with eigenvalues >1. **Top left**, cross correlation matrix for PD-GIS items. **Top right**, question-component loadings after varimax rotation. **Bottom**, scree plot. Note 11^th^ components places above the scree and prior to the 4^th^ inflection point. (All data and models are available for download from the UK Data Service). Source data are provided as a Source Data file.

**Supplementary figure 11 – Sub sampling Train-Test pipeline to evaluate overfit in the PD-GIS by Mental Health Canonical Correlation analysis**


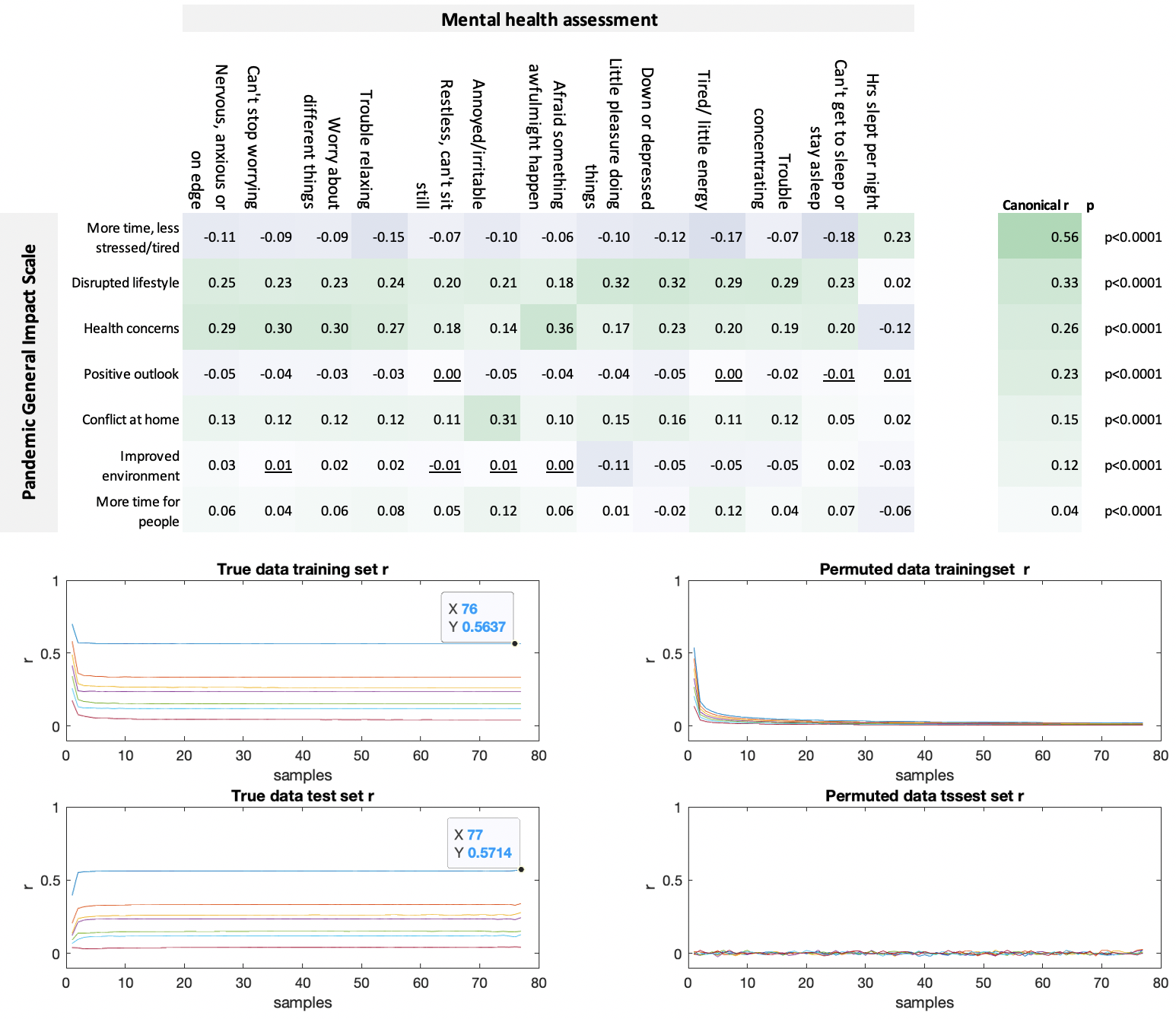


Upper panel left. Bivariate Pearson’s correlations between PD-GIS component scores and scores on Mood Self-Assessment items. Upper panel right. Canonical Correlation mode scores. Middle left. CCA mode scores for trained data sub sampling at different sizes (X axis is in thousands and Y axis is mode correlation value). Middle right, the same analyses conducted for data where the index of the X matrix was permuted, breaking the X-Y matrix linkage whilst retaining their inner structure. Note the near zero scores above 20K samples, indicating little overfit. Bottom left. Mode correlation scores when applying the trained CCA model to the held-out data, to which the model was naïve, with X axis corresponding to the number of participants in the trained set, whereby the held-out set comprises all other participants. Note that canonical r values approximate those of the trained set at higher sample size, indicating little overfit. Bottom right. The analysis of held-out data repeated for the permutated data. Source data are provided as a Source Data file.

**Predicting individual differences in PD-GIS component scores from population variables**

**Supplementary table 17. PD-GIS by sociodemographic factors ANOVA. More time, less stressed & tired**

|  |  | SumSq | DF | MeanSq | F | pValue |
| --- | --- | --- | --- | --- | --- | --- |
|  |  |  |  |  |  |  |
| Demographics | Age | 187 | 1 | 187 | 164 | <0.0001 |
|  | Sex | 124 | 2 | 62 | 55 | <0.0001 |
|  | Handedness | 16 | 2 | 8 | 7 | 0.0007 |
|  | First language | 18 | 1 | 18 | 16 | <0.0001 |
|  | Ethnicity | 152 | 5 | 30 | 27 | <0.0001 |
|  | Country of residence | 1 | 1 | 1 | 1 | 0.2809 |
|  | Education | 192 | 4 | 48 | 42 | <0.0001 |
|  | Relationship status | 24 | 5 | 5 | 4 | 0.0007 |
|  | Home type | 3 | 6 | 0 | 0 | 0.8628 |
|  | Work arrangements | 4296 | 11 | 391 | 343 | <0.0001 |
|  | Income negatively affected | 135 | 1 | 135 | 119 | <0.0001 |
| Cohabitees | adult children | 1 | 1 | 1 | 1 | 0.3620 |
|  | school children | 113 | 1 | 113 | 100 | <0.0001 |
|  | housemates | 1 | 1 | 1 | 1 | 0.3660 |
|  | friends | 2 | 1 | 2 | 2 | 0.2118 |
|  | grandparents | 2 | 1 | 2 | 2 | 0.1765 |
|  | Home schooled children | 48 | 1 | 48 | 42 | <0.0001 |
|  | Inlaws | 0 | 1 | 0 | 0 | 0.7917 |
|  | Alone | 1 | 1 | 1 | 1 | 0.3757 |
|  | Parents | 6 | 1 | 6 | 5 | 0.0252 |
|  | Partner | 1 | 1 | 1 | 1 | 0.2769 |
|  | Preschool children | 841 | 1 | 841 | 739 | <0.0001 |
| Outside space | Balcony | 3 | 1 | 3 | 3 | 0.1007 |
|  | Large garden | 6 | 1 | 6 | 5 | 0.0191 |
|  | None | 18 | 1 | 18 | 16 | <0.0001 |
|  | Overlooked | 51 | 1 | 51 | 45 | <0.0001 |
|  | Private | 8 | 1 | 8 | 7 | 0.0079 |
|  | Relaxing | 132 | 1 | 132 | 116 | <0.0001 |
|  | Small garden | 2 | 1 | 2 | 1 | 0.2336 |
|  | unpleasant | 98 | 1 | 98 | 86 | <0.0001 |
|  | Error | 86370 | 75919 | 1 |  |  |

**Supplementary table 18. PD-GIS by sociodemographic factors ANOVA. Disrupted lifestyle**

|  |  | SumSq | DF | MeanSq | F | pValue |
| --- | --- | --- | --- | --- | --- | --- |
|  |  |  |  |  |  |  |
| Demographics | Age | 1752 | 1 | 1752 | 1711 | <0.0001 |
|  | Sex | 332 | 2 | 166 | 162 | <0.0001 |
|  | Handedness | 24 | 2 | 12 | 12 | <0.0001 |
|  | First language | 328 | 1 | 328 | 321 | <0.0001 |
|  | Ethnicity | 15 | 5 | 3 | 3 | 0.0120 |
|  | Country of residence | 1 | 1 | 1 | 1 | 0.3901 |
|  | Education | 8 | 4 | 2 | 2 | 0.0834 |
|  | Relationship status | 78 | 5 | 16 | 15 | <0.0001 |
|  | Home type | 59 | 6 | 10 | 10 | <0.0001 |
|  | Work arrangements | 2846 | 11 | 259 | 253 | <0.0001 |
|  | Income negatively affected | 1321 | 1 | 1321 | 1291 | <0.0001 |
| Cohabitees | adult children | 9 | 1 | 9 | 9 | 0.0025 |
|  | school children | 10 | 1 | 10 | 10 | 0.0018 |
|  | housemates | 40 | 1 | 40 | 40 | <0.0001 |
|  | friends | 4 | 1 | 4 | 4 | 0.0553 |
|  | grandparents | 1 | 1 | 1 | 1 | 0.4349 |
|  | Home schooled children | 1 | 1 | 1 | 1 | 0.3356 |
|  | Inlaws | 3 | 1 | 3 | 3 | 0.1037 |
|  | Alone | 83 | 1 | 83 | 81 | <0.0001 |
|  | Parents | 7 | 1 | 7 | 7 | 0.0081 |
|  | Partner | 4 | 1 | 4 | 4 | 0.0396 |
|  | Preschool children | 21 | 1 | 21 | 21 | <0.0001 |
| Outside space | Balcony | 1 | 1 | 1 | 1 | 0.3100 |
|  | Large garden | 0 | 1 | 0 | 0 | 0.4879 |
|  | None | 46 | 1 | 46 | 45 | <0.0001 |
|  | Overlooked | 107 | 1 | 107 | 105 | <0.0001 |
|  | Private | 1 | 1 | 1 | 1 | 0.3443 |
|  | Relaxing | 32 | 1 | 32 | 31 | <0.0001 |
|  | Small garden | 13 | 1 | 13 | 13 | 0.0004 |
|  | unpleasant | 108 | 1 | 108 | 106 | <0.0001 |
|  | Error | 77712 | 75919 | 1 |  |  |

**Supplementary table 19. PD-GIS by sociodemographic factors ANOVA. Health concerns**

| Demographics | Age | 144 | 1 | 144 | 109 | <0.0001 |
| --- | --- | --- | --- | --- | --- | --- |
|  | Sex | 1714 | 2 | 857 | 644 | <0.0001 |
|  | Handedness | 3 | 2 | 2 | 1 | 0.3173 |
|  | First language | 96 | 1 | 96 | 72 | <0.0001 |
|  | Ethnicity | 141 | 5 | 28 | 21 | <0.0001 |
|  | Country of residence | 2 | 1 | 2 | 2 | 0.1872 |
|  | Education | 78 | 4 | 19 | 15 | <0.0001 |
|  | Relationship status | 26 | 5 | 5 | 4 | 0.0016 |
|  | Home type | 71 | 6 | 12 | 9 | <0.0001 |
|  | Work arrangements | 549 | 11 | 50 | 37 | <0.0001 |
|  | Income negatively affected | 258 | 1 | 258 | 194 | <0.0001 |
| Cohabitees | adult children | 77 | 1 | 77 | 58 | <0.0001 |
|  | school children | 179 | 1 | 179 | 135 | <0.0001 |
|  | housemates | 7 | 1 | 7 | 6 | 0.0181 |
|  | friends | 4 | 1 | 4 | 3 | 0.0795 |
|  | grandparents | 15 | 1 | 15 | 11 | 0.0010 |
|  | Home schooled children | 24 | 1 | 24 | 18 | <0.0001 |
|  | Inlaws | 0 | 1 | 0 | 0 | 0.7597 |
|  | Alone | 9 | 1 | 9 | 7 | 0.0080 |
|  | Parents | 43 | 1 | 43 | 32 | <0.0001 |
|  | Partner | 17 | 1 | 17 | 13 | 0.0004 |
|  | Preschool children | 0 | 1 | 0 | 0 | 0.6905 |
| Outside space | Balcony | 0 | 1 | 0 | 0 | 0.7740 |
|  | Large garden | 54 | 1 | 54 | 41 | <0.0001 |
|  | None | 8 | 1 | 8 | 6 | 0.0137 |
|  | Overlooked | 62 | 1 | 62 | 47 | <0.0001 |
|  | Private | 2 | 1 | 2 | 2 | 0.2090 |
|  | Relaxing | 48 | 1 | 48 | 36 | <0.0001 |
|  | Small garden | 4 | 1 | 4 | 3 | 0.0985 |
|  | unpleasant | 31 | 1 | 31 | 23 | <0.0001 |
|  | Error | 101030 | 75919 | 1 |  |  |

**Supplementary table 20. PD-GIS by sociodemographic factors ANOVA. Positive outlook**

| Demographics | Age | 7 | 1 | 7 | 5 | 0.0228 |
| --- | --- | --- | --- | --- | --- | --- |
|  | Sex | 684 | 2 | 342 | 261 | <0.0001 |
|  | Handedness | 5 | 2 | 3 | 2 | 0.1294 |
|  | First language | 75 | 1 | 75 | 58 | <0.0001 |
|  | Ethnicity | 206 | 5 | 41 | 31 | <0.0001 |
|  | Country of residence | 3 | 1 | 3 | 2 | 0.1456 |
|  | Education | 480 | 4 | 120 | 92 | <0.0001 |
|  | Relationship status | 48 | 5 | 10 | 7 | <0.0001 |
|  | Home type | 16 | 6 | 3 | 2 | 0.0521 |
|  | Work arrangements | 199 | 11 | 18 | 14 | <0.0001 |
|  | Income negatively affected | 415 | 1 | 415 | 317 | <0.0001 |
| Cohabitees | adult children | 3 | 1 | 3 | 2 | 0.1575 |
|  | school children | 30 | 1 | 30 | 23 | <0.0001 |
|  | housemates | 2 | 1 | 2 | 1 | 0.2545 |
|  | friends | 0 | 1 | 0 | 0 | 0.9235 |
|  | grandparents | 9 | 1 | 9 | 7 | 0.0098 |
|  | Home schooled children | 18 | 1 | 18 | 14 | 0.0002 |
|  | Inlaws | 0 | 1 | 0 | 0 | 0.7003 |
|  | Alone | 15 | 1 | 15 | 11 | 0.0007 |
|  | Parents | 0 | 1 | 0 | 0 | 0.6248 |
|  | Partner | 0 | 1 | 0 | 0 | 0.8729 |
|  | Preschool children | 22 | 1 | 22 | 17 | <0.0001 |
| Outside space | Balcony | 0 | 1 | 0 | 0 | 0.5687 |
|  | Large garden | 2 | 1 | 2 | 1 | 0.2540 |
|  | None | 5 | 1 | 5 | 4 | 0.0511 |
|  | Overlooked | 18 | 1 | 18 | 13 | 0.0002 |
|  | Private | 0 | 1 | 0 | 0 | 0.6835 |
|  | Relaxing | 17 | 1 | 17 | 13 | 0.0003 |
|  | Small garden | 0 | 1 | 0 | 0 | 0.6249 |
|  | unpleasant | 25 | 1 | 25 | 19 | <0.0001 |
|  | Error | 99354 | 75919 | 1 |  |  |

**Supplementary table 21. PD-GIS by sociodemographic factors ANOVA. Increased conflict at home**

| Demographics | Age | 147 | 1 | 147 | 132 | <0.0001 |
| --- | --- | --- | --- | --- | --- | --- |
|  | Sex | 12 | 2 | 6 | 5 | 0.0046 |
|  | Handedness | 5 | 2 | 2 | 2 | 0.1217 |
|  | First language | 4 | 1 | 4 | 4 | 0.0469 |
|  | Ethnicity | 7 | 5 | 1 | 1 | 0.2928 |
|  | Country of residence | 1 | 1 | 1 | 1 | 0.4715 |
|  | Education | 13 | 4 | 3 | 3 | 0.0220 |
|  | Relationship status | 96 | 5 | 19 | 17 | <0.0001 |
|  | Home type | 30 | 6 | 5 | 5 | 0.0001 |
|  | Work arrangements | 119 | 11 | 11 | 10 | <0.0001 |
|  | Income negatively affected | 42 | 1 | 42 | 38 | <0.0001 |
| Cohabitees | adult children | 86 | 1 | 86 | 77 | <0.0001 |
|  | school children | 756 | 1 | 756 | 681 | <0.0001 |
|  | housemates | 0 | 1 | 0 | 0 | 0.8502 |
|  | friends | 0 | 1 | 0 | 0 | 0.8406 |
|  | grandparents | 1 | 1 | 1 | 1 | 0.4107 |
|  | Home schooled children | 334 | 1 | 334 | 301 | <0.0001 |
|  | Inlaws | 24 | 1 | 24 | 21 | <0.0001 |
|  | Alone | 36 | 1 | 36 | 32 | <0.0001 |
|  | Parents | 430 | 1 | 430 | 388 | <0.0001 |
|  | Partner | 17 | 1 | 17 | 15 | <0.0001 |
|  | Preschool children | 424 | 1 | 424 | 382 | <0.0001 |
| Outside space | Balcony | 1 | 1 | 1 | 1 | 0.3852 |
|  | Large garden | 1 | 1 | 1 | 1 | 0.2929 |
|  | None | 1 | 1 | 1 | 1 | 0.4234 |
|  | Overlooked | 5 | 1 | 5 | 4 | 0.0405 |
|  | Private | 0 | 1 | 0 | 0 | 0.6200 |
|  | Relaxing | 47 | 1 | 47 | 42 | <0.0001 |
|  | Small garden | 0 | 1 | 0 | 0 | 0.7396 |
|  | unpleasant | 13 | 1 | 13 | 11 | 0.0007 |
|  | Error | 84236 | 75919 | 1 |  |  |

**Supplementary table 22. PD-GIS by sociodemographic factors ANOVA. Improved environment**

| Demographics | Age | 73 | 1 | 73 | 46 | <0.0001 |
| --- | --- | --- | --- | --- | --- | --- |
|  | Sex | 2623 | 2 | 1312 | 822 | <0.0001 |
|  | Handedness | 45 | 2 | 22 | 14 | <0.0001 |
|  | First language | 186 | 1 | 186 | 117 | <0.0001 |
|  | Ethnicity | 222 | 5 | 44 | 28 | <0.0001 |
|  | Country of residence | 62 | 1 | 62 | 39 | <0.0001 |
|  | Education | 353 | 4 | 88 | 55 | <0.0001 |
|  | Relationship status | 149 | 5 | 30 | 19 | <0.0001 |
|  | Home type | 51 | 6 | 9 | 5 | <0.0001 |
|  | Work arrangements | 601 | 11 | 55 | 34 | <0.0001 |
|  | Income negatively affected | 14 | 1 | 14 | 9 | 0.0027 |
| Cohabitees | adult children | 4 | 1 | 4 | 3 | 0.0992 |
|  | school children | 0 | 1 | 0 | 0 | 0.8859 |
|  | housemates | 0 | 1 | 0 | 0 | 0.7590 |
|  | friends | 6 | 1 | 6 | 4 | 0.0482 |
|  | grandparents | 4 | 1 | 4 | 3 | 0.1056 |
|  | Home schooled children | 1 | 1 | 1 | 1 | 0.4578 |
|  | Inlaws | 1 | 1 | 1 | 1 | 0.4747 |
|  | Alone | 20 | 1 | 20 | 13 | 0.0004 |
|  | Parents | 3 | 1 | 3 | 2 | 0.1986 |
|  | Partner | 3 | 1 | 3 | 2 | 0.1930 |
|  | Preschool children | 9 | 1 | 9 | 5 | 0.0194 |
| Outside space | Balcony | 14 | 1 | 14 | 9 | 0.0035 |
|  | Large garden | 63 | 1 | 63 | 40 | <0.0001 |
|  | None | 38 | 1 | 38 | 24 | <0.0001 |
|  | Overlooked | 38 | 1 | 38 | 24 | <0.0001 |
|  | Private | 12 | 1 | 12 | 8 | 0.0058 |
|  | Relaxing | 237 | 1 | 237 | 148 | <0.0001 |
|  | Small garden | 11 | 1 | 11 | 7 | 0.0092 |
|  | unpleasant | 96 | 1 | 96 | 60 | <0.0001 |
|  | Error | 121070 | 75919 | 2 |  |  |

**Supplementary table 23. PD-GIS by sociodemographic factors ANOVA. More time for people**

| Demographics | Age | 916 | 1 | 916 | 698 | <0.0001 |
| --- | --- | --- | --- | --- | --- | --- |
|  | Sex | 73 | 2 | 37 | 28 | <0.0001 |
|  | Handedness | 3 | 2 | 2 | 1 | 0.2765 |
|  | First language | 36 | 1 | 36 | 27 | <0.0001 |
|  | Ethnicity | 77 | 5 | 15 | 12 | <0.0001 |
|  | Country of residence | 0 | 1 | 0 | 0 | 0.9749 |
|  | Education | 11 | 4 | 3 | 2 | 0.0755 |
|  | Relationship status | 129 | 5 | 26 | 20 | <0.0001 |
|  | Home type | 79 | 6 | 13 | 10 | <0.0001 |
|  | Work arrangements | 1700 | 11 | 155 | 118 | <0.0001 |
|  | Income negatively affected | 42 | 1 | 42 | 32 | <0.0001 |
| Cohabitees | adult children | 250 | 1 | 250 | 191 | <0.0001 |
|  | school children | 1486 | 1 | 1486 | 1133 | <0.0001 |
|  | housemates | 460 | 1 | 460 | 351 | <0.0001 |
|  | friends | 14 | 1 | 14 | 11 | 0.0012 |
|  | grandparents | 6 | 1 | 6 | 4 | 0.0387 |
|  | Home schooled children | 811 | 1 | 811 | 618 | <0.0001 |
|  | Inlaws | 11 | 1 | 11 | 8 | 0.0037 |
|  | Alone | 1682 | 1 | 1682 | 1282 | <0.0001 |
|  | Parents | 172 | 1 | 172 | 131 | <0.0001 |
|  | Partner | 164 | 1 | 164 | 125 | <0.0001 |
|  | Preschool children | 1435 | 1 | 1435 | 1094 | <0.0001 |
| Outside space | Balcony | 1 | 1 | 1 | 1 | 0.3519 |
|  | Large garden | 20 | 1 | 20 | 16 | <0.0001 |
|  | None | 1 | 1 | 1 | 1 | 0.3502 |
|  | Overlooked | 34 | 1 | 34 | 26 | <0.0001 |
|  | Private | 1 | 1 | 1 | 1 | 0.3832 |
|  | Relaxing | 35 | 1 | 35 | 27 | <0.0001 |
|  | Small garden | 13 | 1 | 13 | 10 | 0.0017 |
|  | unpleasant | 4 | 1 | 4 | 3 | 0.0873 |
|  | Error | 99584 | 75919 | 1 |  |  |

**Supplementary table 24. PD-GIS by sociodemographic factors. Parameter estimates in standard deviation (SD) units**

Parameter estimates for predictors in the GLM. p<0.05*, p<0..01**, p<0.001. All predictors are binary and can be interpreted as effect sizes in standard deviation units (apart from age, which is reported separately).

Continued overleaf -->

Parameter estimates for predictors in the GLM. p<0.05*, p<0..01**, p<0.001. All predictors are binary and can be interpreted as effect sizes in standard deviation units (apart from age, which is reported separately). Effects sizes highlighted in blue (negative) and green (positive).

**Supplementary table 25. PD-GIS by pre-existing conditions. More time, less stressed and tired. (Parameter estimates in SD units)**

|  | estimate | N | SE | t | p |
| --- | --- | --- | --- | --- | --- |
| Anxiety | -0.028 | 4141 | 0 | -2 | 0.0799 |
| Attention deficit hyperactivity disorder | -0.007 | 485 | 0 | 0 | 0.8850 |
| Bipolar | -0.059 | 363 | 0 | -1 | 0.2609 |
| Depression | -0.097 | 4744 | 0 | -6 | <0.0001 |
| Obsessive compulsive disorder | 0.064 | 241 | 0 | 1 | 0.3218 |
| Other psychiatric | -0.011 | 1157 | 0 | 0 | 0.7062 |
| Learning disability | -0.081 | 890 | 0 | -2 | 0.0173 |
| Multiple sclerosis | -0.109 | 243 | 0 | -2 | 0.0903 |
| Stroke | 0.060 | 441 | 0 | 1 | 0.2117 |
| Other neurological | -0.024 | 2093 | 0 | -1 | 0.2906 |
| Traumatic brain injury | 0.049 | 110 | 0 | 1 | 0.6088 |
| Parkinson's disease | -0.158 | 122 | 0 | -2 | 0.0810 |
| OCD & anxiety | -0.119 | 602 | 0 | -3 | 0.0053 |
| Depression & anxiety | -0.159 | 5979 | 0 | -11 | <0.0001 |
| Weakened immune system | -0.045 | 2082 | 0 | -2 | 0.0443 |
| Kidney disease | -0.025 | 545 | 0 | -1 | 0.5638 |
| Diabetes | -0.034 | 2858 | 0 | -2 | 0.0749 |
| Heart disease | -0.027 | 2161 | 0 | -1 | 0.2227 |
| High blood pressure | 0.014 | 744 | 0 | 0 | 0.7138 |
| Irregular heart beat | 0.042 | 181 | 0 | 1 | 0.5761 |
| Liver disease | 0.034 | 364 | 0 | 1 | 0.5223 |
| Lung condition | -0.032 | 8226 | 0 | -3 | 0.0064 |
| Spleen/ sickle cell disease | -0.015 | 153 | 0 | 0 | 0.8492 |

**Supplementary table 26. PD-GIS by pre-existing conditions. Disrupted lifestyle. (Parameter estimates in SD units)**

|  | estimate | N | SE | t |  |
| --- | --- | --- | --- | --- | --- |
| Anxiety | 0.092 | 4141 | 0 | 6 | <0.0001 |
| Attention deficit hyperactivity disorder | 0.123 | 485 | 0 | 3 | 0.0074 |
| Bipolar | 0.109 | 363 | 0 | 2 | 0.0392 |
| Depression | 0.259 | 4744 | 0 | 17 | <0.0001 |
| Obsessive compulsive disorder | -0.046 | 241 | 0 | -1 | 0.4718 |
| Other psychiatric | 0.025 | 1157 | 0 | 1 | 0.4128 |
| Learning disability | -0.047 | 890 | 0 | -1 | 0.1601 |
| Multiple sclerosis | -0.038 | 243 | 0 | -1 | 0.5555 |
| Stroke | 0.019 | 441 | 0 | 0 | 0.6942 |
| Other neurological | -0.044 | 2093 | 0 | -2 | 0.0459 |
| Traumatic brain injury | -0.050 | 110 | 0 | -1 | 0.6006 |
| Parkinson's disease | 0.016 | 122 | 0 | 0 | 0.8621 |
| OCD & anxiety | -0.037 | 602 | 0 | -1 | 0.3883 |
| Depression & anxiety | 0.301 | 5979 | 0 | 22 | <0.0001 |
| Weakened immune system | 0.040 | 2082 | 0 | 2 | 0.0741 |
| Kidney disease | 0.031 | 545 | 0 | 1 | 0.4758 |
| Diabetes | 0.046 | 2858 | 0 | 2 | 0.0153 |
| Heart disease | 0.074 | 2161 | 0 | 3 | 0.0007 |
| High blood pressure | -0.004 | 744 | 0 | 0 | 0.9091 |
| Irregular heart beat | -0.032 | 181 | 0 | 0 | 0.6695 |
| Liver disease | 0.030 | 364 | 0 | 1 | 0.5709 |
| Lung condition | 0.024 | 8226 | 0 | 2 | 0.0371 |
| Spleen/ sickle cell | 0.060 | 153 | 0 | 1 | 0.4540 |

**Supplementary table 27. PD-GIS by pre-existing conditions. Increased health concerns. (Parameter estimates in SD units)**

|  | estimate | N | SE | t |  |
| --- | --- | --- | --- | --- | --- |
| Anxiety | 0.301 | 4141 | 0 | 19 | <0.0001 |
| Attention deficit hyperactivity disorder | -0.013 | 485 | 0 | 0 | 0.7689 |
| Bipolar | 0.010 | 363 | 0 | 0 | 0.8503 |
| Depression | 0.056 | 4744 | 0 | 4 | 0.0003 |
| Obsessive compulsive disorder | 0.226 | 241 | 0 | 4 | 0.0004 |
| Other psychiatric | 0.006 | 1157 | 0 | 0 | 0.8389 |
| Learning disability | 0.040 | 890 | 0 | 1 | 0.2384 |
| Multiple sclerosis | 0.009 | 243 | 0 | 0 | 0.8822 |
| Stroke | -0.019 | 441 | 0 | 0 | 0.6851 |
| Other neurological | 0.040 | 2093 | 0 | 2 | 0.0727 |
| Traumatic brain injury | 0.030 | 110 | 0 | 0 | 0.7493 |
| Parkinson's disease | 0.005 | 122 | 0 | 0 | 0.9519 |
| OCD & anxiety | 0.486 | 602 | 0 | 12 | <0.0001 |
| Depression & anxiety | 0.278 | 5979 | 0 | 20 | <0.0001 |
| Weakened immune system | 0.265 | 2082 | 0 | 12 | <0.0001 |
| Kidney disease | 0.111 | 545 | 0 | 3 | 0.0095 |
| Diabetes | 0.212 | 2858 | 0 | 11 | <0.0001 |
| Heart disease | 0.115 | 2161 | 0 | 5 | <0.0001 |
| High blood pressure | 0.051 | 744 | 0 | 1 | 0.1646 |
| Irregular heart beat | 0.173 | 181 | 0 | 2 | 0.0200 |
| Liver disease | 0.056 | 364 | 0 | 1 | 0.2822 |
| Lung condition | 0.220 | 8226 | 0 | 19 | <0.0001 |
| Spleen/ sickle cell | 0.104 | 153 | 0 | 1 | 0.1960 |

**Supplementary table 28. PD-GIS by pre-existing conditions. Positive outlook. (Parameter estimates in SD units)**

|  | estimate | N | SE | t |  |
| --- | --- | --- | --- | --- | --- |
| Anxiety | 0.034 | 4141 | 0 | 2 | 0.0341 |
| Attention deficit hyperactivity disorder | 0.086 | 485 | 0 | 2 | 0.0616 |
| Bipolar | 0.047 | 363 | 0 | 1 | 0.3791 |
| Depression | -0.040 | 4744 | 0 | -3 | 0.0109 |
| Obsessive compulsive disorder | 0.006 | 241 | 0 | 0 | 0.9278 |
| Other psychiatric | -0.038 | 1157 | 0 | -1 | 0.2134 |
| Learning disability | 0.058 | 890 | 0 | 2 | 0.0879 |
| Multiple sclerosis | -0.075 | 243 | 0 | -1 | 0.2454 |
| Stroke | 0.100 | 441 | 0 | 2 | 0.0365 |
| Other neurological | -0.006 | 2093 | 0 | 0 | 0.7842 |
| Traumatic brain injury | 0.095 | 110 | 0 | 1 | 0.3180 |
| Parkinson's disease | 0.009 | 122 | 0 | 0 | 0.9235 |
| OCD & anxiety | 0.044 | 602 | 0 | 1 | 0.2979 |
| Depression & anxiety | -0.029 | 5979 | 0 | -2 | 0.0342 |
| Weakened immune system | 0.062 | 2082 | 0 | 3 | 0.0056 |
| Kidney disease | 0.021 | 545 | 0 | 0 | 0.6202 |
| Diabetes | 0.089 | 2858 | 0 | 5 | <0.0001 |
| Heart disease | 0.011 | 2161 | 0 | 1 | 0.6064 |
| High blood pressure | -0.137 | 744 | 0 | -4 | 0.0002 |
| Irregular heart beat | -0.041 | 181 | 0 | -1 | 0.5895 |
| Liver disease | -0.064 | 364 | 0 | -1 | 0.2274 |
| Lung condition | 0.007 | 8226 | 0 | 1 | 0.5695 |
| Spleen/ sickle cell | 0.067 | 153 | 0 | 1 | 0.4087 |

**Supplementary table 29. PD-GIS by pre-existing conditions. Conflict at home. (Parameter estimates in SD units)**

|  | estimate | N | SE | t |  |
| --- | --- | --- | --- | --- | --- |
| Anxiety | -0.009 | 4141 | 0 | -1 | 0.5950 |
| Attention deficit hyperactivity disorder | 0.176 | 485 | 0 | 4 | 0.0001 |
| Bipolar | -0.097 | 363 | 0 | -2 | 0.0663 |
| Depression | 0.081 | 4744 | 0 | 5 | <0.0001 |
| Obsessive compulsive disorder | 0.110 | 241 | 0 | 2 | 0.0885 |
| Other psychiatric | 0.094 | 1157 | 0 | 3 | 0.0020 |
| Learning disability | 0.046 | 890 | 0 | 1 | 0.1788 |
| Multiple sclerosis | 0.061 | 243 | 0 | 1 | 0.3431 |
| Stroke | 0.042 | 441 | 0 | 1 | 0.3827 |
| Other neurological | 0.072 | 2093 | 0 | 3 | 0.0013 |
| Traumatic brain injury | 0.071 | 110 | 0 | 1 | 0.4591 |
| Parkinson's disease | -0.002 | 122 | 0 | 0 | 0.9822 |
| OCD & anxiety | 0.016 | 602 | 0 | 0 | 0.7010 |
| Depression & anxiety | 0.066 | 5979 | 0 | 5 | <0.0001 |
| Weakened immune system | -0.039 | 2082 | 0 | -2 | 0.0817 |
| Kidney disease | -0.026 | 545 | 0 | -1 | 0.5555 |
| Diabetes | -0.014 | 2858 | 0 | -1 | 0.4803 |
| Heart disease | -0.012 | 2161 | 0 | -1 | 0.5876 |
| High blood pressure | 0.044 | 744 | 0 | 1 | 0.2397 |
| Irregular heart beat | 0.039 | 181 | 0 | 1 | 0.6027 |
| Liver disease | 0.025 | 364 | 0 | 0 | 0.6295 |
| Lung condition | -0.030 | 8226 | 0 | -3 | 0.0100 |
| Spleen/ sickle cell | 0.023 | 153 | 0 | 0 | 0.7775 |

**Supplementary table 30. PD-GIS by pre-existing conditions. Improved environment. (Parameter estimates in SD units)**

|  | estimate | N | SE | t |  |
| --- | --- | --- | --- | --- | --- |
| Anxiety | 0.036 | 4141 | 0 | 2 | 0.0243 |
| Attention deficit hyperactivity disorder | -0.132 | 485 | 0 | -3 | 0.0042 |
| Bipolar | -0.220 | 363 | 0 | -4 | <0.0001 |
| Depression | 0.013 | 4744 | 0 | 1 | 0.4040 |
| Obsessive compulsive disorder | -0.015 | 241 | 0 | 0 | 0.8137 |
| Other psychiatric | -0.006 | 1157 | 0 | 0 | 0.8546 |
| Learning disability | -0.070 | 890 | 0 | -2 | 0.0384 |
| Multiple sclerosis | -0.016 | 243 | 0 | 0 | 0.8013 |
| Stroke | -0.069 | 441 | 0 | -1 | 0.1508 |
| Other neurological | -0.103 | 2093 | 0 | -5 | <0.0001 |
| Traumatic brain injury | 0.035 | 110 | 0 | 0 | 0.7139 |
| Parkinson's disease | -0.247 | 122 | 0 | -3 | 0.0064 |
| OCD & anxiety | -0.076 | 602 | 0 | -2 | 0.0749 |
| Depression & anxiety | 0.017 | 5979 | 0 | 1 | 0.2095 |
| Weakened immune system | -0.032 | 2082 | 0 | -1 | 0.1530 |
| Kidney disease | -0.143 | 545 | 0 | -3 | 0.0010 |
| Diabetes | -0.168 | 2858 | 0 | -9 | <0.0001 |
| Heart disease | -0.060 | 2161 | 0 | -3 | 0.0062 |
| High blood pressure | -0.004 | 744 | 0 | 0 | 0.9216 |
| Irregular heart beat | -0.136 | 181 | 0 | -2 | 0.0709 |
| Liver disease | -0.110 | 364 | 0 | -2 | 0.0369 |
| Lung condition | -0.015 | 8226 | 0 | -1 | 0.2047 |
| Spleen/ sickle cell | -0.050 | 153 | 0 | -1 | 0.5374 |

**Supplementary table 31. PD-GIS by pre-existing conditions. More time for people at home. (Parameter estimates in SD units)**

|  | estimate | N | SE | t |  |
| --- | --- | --- | --- | --- | --- |
| Anxiety | 0.090 | 4141 | 0 | 6 |  |
| Attention deficit hyperactivity disorder | 0.063 | 485 | 0 | 1 | 0.1683 |
| Bipolar | 0.069 | 363 | 0 | 1 | 0.1894 |
| Depression | 0.026 | 4744 | 0 | 2 | 0.0904 |
| Obsessive compulsive disorder | 0.008 | 241 | 0 | 0 | 0.9060 |
| Other psychiatric | -0.044 | 1157 | 0 | -1 | 0.1521 |
| Learning disability | 0.040 | 890 | 0 | 1 | 0.2333 |
| Multiple sclerosis | -0.071 | 243 | 0 | -1 | 0.2685 |
| Stroke | 0.010 | 441 | 0 | 0 | 0.8301 |
| Other neurological | 0.001 | 2093 | 0 | 0 | 0.9510 |
| Traumatic brain injury | 0.065 | 110 | 0 | 1 | 0.4944 |
| Parkinson's disease | -0.013 | 122 | 0 | 0 | 0.8896 |
| OCD & anxiety | 0.033 | 602 | 0 | 1 | 0.4421 |
| Depression & anxiety | 0.112 | 5979 | 0 | 8 | <0.0001 |
| Weakened immune system | 0.092 | 2082 | 0 | 4 | <0.0001 |
| Kidney disease | 0.065 | 545 | 0 | 1 | 0.1358 |
| Diabetes | 0.117 | 2858 | 0 | 6 | <0.0001 |
| Heart disease | 0.028 | 2161 | 0 | 1 | 0.2048 |
| High blood pressure | 0.012 | 744 | 0 | 0 | 0.7494 |
| Irregular heart beat | 0.177 | 181 | 0 | 2 | 0.0187 |
| Liver disease | 0.049 | 364 | 0 | 1 | 0.3565 |
| Lung condition | 0.067 | 8226 | 0 | 6 | <0.0001 |
| Spleen/ sickle cell | 0.125 | 153 | 0 | 2 | 0.1222 |

**Principal Component Analyses of questionnaire scales.**

Note – here we conform to the Kaiser convention of including components with eigenvalues > 1 in all analyses.

**Big 5 optimised (reduced sub-set of Big5 personality measures)**

**Supplementary table 32. Bivariate correlations for the Big 5 personality**

|  | **Talkative** | **Thorough job** | **Original/ new ideas** | **reserved** | **Relaxed, handles stress well** | **Forgiving** | **Disorgansied** | **Worries a lot** | **tends to be queit** | **Stable/ not easily upset** | **inventive** | **Perseveres till the task is finished** | **Values artistic/aesthetci experiences** | **Kind/considerate to almost everyone** | **Likes to reflect/play with ideas** | **Few artistic instincts** | **Cooperative** | **Sophisticated in art/music/literature** |
| --- | --- | --- | --- | --- | --- | --- | --- | --- | --- | --- | --- | --- | --- | --- | --- | --- | --- | --- |
| **Q1** |  | 0.09 | 0.21 | -0.53 | 0.03 | 0.08 | 0.05 | 0.01 | -0.42 | -0.02 | 0.11 | 0.03 | 0.11 | 0.14 | 0.08 | -0.03 | 0.19 | 0.11 |
| **Q2** | 0.09 |  | 0.16 | 0.04 | 0.09 | 0.05 | -0.32 | 0.01 | -0.13 | 0.12 | 0.11 | 0.53 | 0.09 | 0.20 | 0.11 | 0.00 | 0.17 | 0.04 |
| **Q3** | 0.21 | 0.16 |  | -0.12 | 0.17 | 0.09 | 0.06 | -0.08 | -0.12 | 0.12 | 0.69 | 0.16 | 0.29 | 0.11 | 0.43 | -0.15 | 0.06 | 0.25 |
| **Q4** | -0.53 | 0.04 | -0.12 |  | -0.05 | 0.00 | 0.03 | 0.18 | 0.49 | -0.01 | -0.07 | 0.04 | -0.02 | 0.00 | 0.03 | 0.06 | -0.07 | -0.03 |
| **Q5** | 0.03 | 0.09 | 0.17 | -0.05 |  | 0.24 | -0.05 | -0.54 | -0.04 | 0.59 | 0.18 | 0.12 | 0.00 | 0.12 | 0.10 | 0.04 | 0.14 | 0.02 |
| **Q6** | 0.08 | 0.05 | 0.09 | 0.00 | 0.24 |  | 0.09 | -0.06 | 0.00 | 0.21 | 0.10 | 0.08 | 0.13 | 0.43 | 0.17 | -0.02 | 0.32 | 0.08 |
| **Q7** | 0.05 | -0.32 | 0.06 | 0.03 | -0.05 | 0.09 |  | 0.12 | 0.19 | -0.10 | 0.08 | -0.29 | 0.05 | -0.01 | 0.10 | 0.00 | -0.04 | 0.05 |
| **Q8** | 0.01 | 0.01 | -0.08 | 0.18 | -0.54 | -0.06 | 0.12 |  | 0.14 | -0.48 | -0.10 | -0.02 | 0.09 | 0.07 | 0.03 | 0.00 | 0.00 | 0.05 |
| **Q9** | -0.42 | -0.13 | -0.12 | 0.49 | -0.04 | 0.00 | 0.19 | 0.14 |  | -0.03 | -0.07 | -0.12 | -0.01 | -0.02 | 0.01 | 0.06 | -0.10 | -0.01 |
| **Q10** | -0.02 | 0.12 | 0.12 | -0.01 | 0.59 | 0.21 | -0.10 | -0.48 | -0.03 |  | 0.17 | 0.17 | -0.01 | 0.11 | 0.08 | 0.04 | 0.15 | 0.00 |
| **Q11** | 0.11 | 0.11 | 0.69 | -0.07 | 0.18 | 0.10 | 0.08 | -0.10 | -0.07 | 0.17 |  | 0.18 | 0.30 | 0.09 | 0.44 | -0.16 | 0.04 | 0.25 |
| **Q12** | 0.03 | 0.53 | 0.16 | 0.04 | 0.12 | 0.08 | -0.29 | -0.02 | -0.12 | 0.17 | 0.18 |  | 0.12 | 0.18 | 0.15 | 0.00 | 0.17 | 0.04 |
| **Q13** | 0.11 | 0.09 | 0.29 | -0.02 | 0.00 | 0.13 | 0.05 | 0.09 | -0.01 | -0.01 | 0.30 | 0.12 |  | 0.22 | 0.37 | -0.41 | 0.14 | 0.56 |
| **Q14** | 0.14 | 0.20 | 0.11 | 0.00 | 0.12 | 0.43 | -0.01 | 0.07 | -0.02 | 0.11 | 0.09 | 0.18 | 0.22 |  | 0.23 | -0.04 | 0.41 | 0.13 |
| **Q15** | 0.08 | 0.11 | 0.43 | 0.03 | 0.10 | 0.17 | 0.10 | 0.03 | 0.01 | 0.08 | 0.44 | 0.15 | 0.37 | 0.23 |  | -0.15 | 0.16 | 0.29 |
| **Q16** | -0.03 | 0.00 | -0.15 | 0.06 | 0.04 | -0.02 | 0.00 | 0.00 | 0.06 | 0.04 | -0.16 | 0.00 | -0.41 | -0.04 | -0.15 |  | 0.00 | -0.37 |
| **Q17** | 0.19 | 0.17 | 0.06 | -0.07 | 0.14 | 0.32 | -0.04 | 0.00 | -0.10 | 0.15 | 0.04 | 0.17 | 0.14 | 0.41 | 0.16 | 0.00 |  | 0.09 |
| **Q18** | 0.11 | 0.04 | 0.25 | -0.03 | 0.02 | 0.08 | 0.05 | 0.05 | -0.01 | 0.00 | 0.25 | 0.04 | 0.56 | 0.13 | 0.29 | -0.37 | 0.09 |  |

**Supplementary figure 12. Principle component analysis with varimax rotation for the Big 5 personality**

Top left – correlation matrix for the Big 5 optimised personality scale. Top right, task-component loadings after varimax rotation. Bottom – scree plot of eigenvalues. Source data are provided as a Source Data file.

**Supplementary table 33. Varimax rotated component loadings table for the Big 5 personality.**

| **Secure** | **Open** | **Introverted** | **Artistic** | **Compassionate** | **Conscientious** |  |
| --- | --- | --- | --- | --- | --- | --- |
| -0.06 | 0.12 | **-0.67** | 0.05 | 0.20 | 0.00 | Talkative |
| 0.00 | 0.12 | -0.03 | 0.01 | 0.16 | **0.72** | Thorough job |
| 0.09 | **0.80** | -0.14 | 0.16 | 0.03 | 0.06 | Original/ new ideas |
| -0.09 | -0.01 | **0.80** | -0.03 | 0.02 | 0.08 | reserved |
| **0.76** | 0.12 | 0.00 | -0.02 | 0.19 | 0.05 | Relaxed, handles stress well |
| 0.17 | 0.07 | 0.01 | 0.03 | **0.59** | -0.06 | Forgiving |
| -0.11 | 0.14 | 0.07 | 0.02 | 0.10 | **-0.49** | Disorganised |
| **-0.73** | 0.00 | 0.13 | 0.03 | 0.10 | -0.01 | Worries a lot |
| -0.06 | -0.01 | **0.63** | -0.01 | 0.02 | -0.19 | tends to be quiet |
| **0.70** | 0.09 | 0.04 | -0.03 | 0.17 | 0.12 | Stable/ not easily upset |
| 0.13 | **0.80** | -0.05 | 0.18 | 0.01 | 0.04 | inventive |
| 0.05 | 0.16 | 0.01 | 0.03 | 0.15 | **0.68** | Perseveres till the task is finished |
| -0.06 | 0.21 | -0.01 | **0.76** | 0.19 | 0.04 | Values artistic/aesthetic experiences |
| -0.01 | 0.07 | -0.02 | 0.10 | **0.68** | 0.11 | Kind/considerate to almost everyone |
| 0.00 | **0.48** | 0.04 | 0.28 | 0.25 | 0.03 | Likes to reflect/play with ideas |
| 0.01 | -0.07 | 0.05 | **-0.54** | 0.04 | 0.01 | Few artistic instincts |
| 0.05 | 0.01 | -0.12 | 0.05 | **0.56** | 0.12 | Cooperative |
| -0.02 | 0.17 | -0.02 | **0.66** | 0.09 | -0.01 | Sophisticated in art/music/literature |

**Supplementary table 34. Bivariate correlations for the technology use scale**

|  | **Smart phone** | **Computer** | **Tablet** | **Games console** | **Email** | **Social media** | **News** | **Games** | **Gamble** | **Work** | **Learn/study** | **Shop** | **Stream** | **Search** | **Hrs per day** |
| --- | --- | --- | --- | --- | --- | --- | --- | --- | --- | --- | --- | --- | --- | --- | --- |
| **Q1** |  | 0.25 | 0.04 | 0.16 | 0.31 | 0.41 | 0.16 | 0.09 | 0.09 | 0.26 | 0.17 | 0.20 | 0.32 | 0.34 | 0.31 |
| **Q2** | 0.25 |  | 0.02 | 0.09 | 0.38 | 0.08 | 0.18 | 0.12 | 0.04 | 0.53 | 0.24 | 0.14 | 0.20 | 0.29 | 0.32 |
| **Q3** | 0.04 | 0.02 |  | 0.02 | 0.16 | -0.01 | 0.10 | 0.06 | 0.01 | 0.05 | 0.00 | 0.16 | -0.05 | 0.06 | -0.03 |
| **Q4** | 0.16 | 0.09 | 0.02 |  | -0.01 | 0.13 | -0.02 | 0.56 | 0.21 | 0.07 | 0.14 | 0.05 | 0.30 | 0.10 | 0.22 |
| **Q5** | 0.31 | 0.38 | 0.16 | -0.01 |  | 0.14 | 0.29 | 0.00 | 0.05 | 0.40 | 0.09 | 0.25 | 0.10 | 0.31 | 0.20 |
| **Q6** | 0.41 | 0.08 | -0.01 | 0.13 | 0.14 |  | 0.05 | 0.08 | 0.07 | 0.11 | 0.16 | 0.14 | 0.27 | 0.22 | 0.31 |
| **Q7** | 0.16 | 0.18 | 0.10 | -0.02 | 0.29 | 0.05 |  | 0.01 | 0.05 | 0.18 | 0.08 | 0.13 | 0.08 | 0.26 | 0.09 |
| **Q8** | 0.09 | 0.12 | 0.06 | 0.56 | 0.00 | 0.08 | 0.01 |  | 0.16 | 0.03 | 0.09 | 0.04 | 0.20 | 0.10 | 0.21 |
| **Q9** | 0.09 | 0.04 | 0.01 | 0.21 | 0.05 | 0.07 | 0.05 | 0.16 |  | 0.06 | 0.02 | 0.08 | 0.11 | 0.04 | 0.09 |
| **Q10** | 0.26 | 0.53 | 0.05 | 0.07 | 0.40 | 0.11 | 0.18 | 0.03 | 0.06 |  | 0.32 | 0.19 | 0.23 | 0.33 | 0.29 |
| **Q11** | 0.17 | 0.24 | 0.00 | 0.14 | 0.09 | 0.16 | 0.08 | 0.09 | 0.02 | 0.32 |  | 0.13 | 0.30 | 0.28 | 0.26 |
| **Q12** | 0.20 | 0.14 | 0.16 | 0.05 | 0.25 | 0.14 | 0.13 | 0.04 | 0.08 | 0.19 | 0.13 |  | 0.20 | 0.23 | 0.15 |
| **Q13** | 0.32 | 0.20 | -0.05 | 0.30 | 0.10 | 0.27 | 0.08 | 0.20 | 0.11 | 0.23 | 0.30 | 0.20 |  | 0.35 | 0.36 |
| **Q14** | 0.34 | 0.29 | 0.06 | 0.10 | 0.31 | 0.22 | 0.26 | 0.10 | 0.04 | 0.33 | 0.28 | 0.23 | 0.35 |  | 0.33 |
| **Q15** | 0.31 | 0.32 | -0.03 | 0.22 | 0.20 | 0.31 | 0.09 | 0.21 | 0.09 | 0.29 | 0.26 | 0.15 | 0.36 | 0.33 |  |

**Supplementary figure 13. Principle component analysis with varimax rotation for technology use**

Top left – correlation matrix for items of the technology use scale. Top right, task-component loadings after varimax rotation. Bottom – scree plot of eigenvalues. Source data are provided as a Source Data file.

**Supplementary table 35. Varimax rotated component loadings table for the technology use scale**

| **Phone, social, entertainment** | **Computer, Work, study** | **gaming** | **Email, news and info** |  |
| --- | --- | --- | --- | --- |
| 0.567 | 0.154 | 0.090 | 0.258 | Smart phone |
| 0.092 | 0.651 | 0.080 | 0.241 | Computer |
| -0.035 | -0.041 | 0.062 | 0.301 | Tablet |
| 0.140 | 0.065 | 0.774 | -0.046 | Games console |
| 0.164 | 0.325 | -0.039 | 0.652 | Email |
| 0.586 | 0.000 | 0.073 | 0.063 | Social media |
| 0.118 | 0.153 | -0.024 | 0.335 | News |
| 0.036 | 0.059 | 0.719 | 0.012 | Games |
| 0.086 | 0.015 | 0.235 | 0.067 | Gamble |
| 0.155 | 0.691 | 0.002 | 0.233 | Work |
| 0.286 | 0.394 | 0.089 | -0.094 | Learn/study |
| 0.254 | 0.119 | 0.037 | 0.266 | Shop |
| 0.515 | 0.262 | 0.252 | -0.086 | Stream |
| 0.434 | 0.332 | 0.054 | 0.216 | Search |
| 0.442 | 0.338 | 0.200 | 0.023 | Hrs per day |

**Supplementary table 36. Bivariate correlations for the technology stress scale**

|  | **Q1** | **Q2** | **Q3** | **Q4** |
| --- | --- | --- | --- | --- |
| Email |  | 0.19 | 0.15 | 0.03 |
| Social media | 0.19 |  | 0.17 | 0.12 |
| News | 0.15 | 0.17 |  | -0.02 |
| Computer games | 0.03 | 0.12 | -0.02 |  |

**Supplementary figure 14. Principle component analysis with varimax rotation for technology stress**

Top left – correlation matrix for the technology stress scale items. Top right, task-component loadings after varimax rotation. Bottom – scree plot of eigenvalues. Source data are provided as a Source Data file.

**Supplementary table 37. Varimax rotated component loadings table for technology stress**

| **Loadings** | **Q - stress from** |
| --- | --- |
| 0.35 | Email |
| 0.56 | Social media |
| 0.32 | News |
| 0.15 | Computer games |

**Supplementary table 38. Bivariate correlations for the technology addiction scale**

|  | **Q1** | **Q2** | **Q3** | **Q4** | **Q5** | **Q6** | **Q7** |
| --- | --- | --- | --- | --- | --- | --- | --- |
| **Q1** |  | 0.37 | 0.34 | 0.15 | 0.43 | 0.29 | 0.28 |
| **Q2** | 0.37 |  | 0.58 | 0.24 | 0.41 | 0.41 | 0.36 |
| **Q3** | 0.34 | 0.58 |  | 0.23 | 0.45 | 0.42 | 0.36 |
| **Q4** | 0.15 | 0.24 | 0.23 |  | 0.21 | 0.30 | 0.25 |
| **Q5** | 0.43 | 0.41 | 0.45 | 0.21 |  | 0.50 | 0.44 |
| **Q6** | 0.29 | 0.41 | 0.42 | 0.30 | 0.50 |  | 0.68 |
| **Q7** | 0.28 | 0.36 | 0.36 | 0.25 | 0.44 | 0.68 |  |

**Supplementary table 15. Principle component analysis with varimax rotation for technology addiction**

Top left – correlation matrix for the technology addiction scale items. Top right, task-component loadings after varimax rotation. Bottom – scree plot of eigenvalues. Source data are provided as a Source Data file.

**Supplementary table 39. Varimax rotated component loadings table for technology addiction**

| 0.48 | Q1 Check email/social media in bed |
| --- | --- |
| 0.62 | Q2 Internet activities to sooth self / block out disturbing thoughts |
| 0.63 | Q3 Time online to battle loneliness |
| 0.36 | Q4 Negative financial consequences due to online pursuits |
| 0.67 | Q5 Check email/social media before something important |
| 0.77 | Q6 Try to stop online activity but feel compelled |
| 0.71 | Q7 Try to cut down time online but fail |

**Supplementary table 40. Bivariate correlations for the compulsivity scale (CHIT)**

|  | **Q1** | **Q2** | **Q3** | **Q4** | **Q5** | **Q6** | **Q7** | **Q8** | **Q9** | **Q10** | **Q11** | **Q12** | **Q13** | **Q14** |
| --- | --- | --- | --- | --- | --- | --- | --- | --- | --- | --- | --- | --- | --- | --- |
| **Q1** |  | 0.45 | 0.40 | 0.10 | 0.16 | 0.02 | 0.07 | -0.04 | -0.11 | 0.27 | 0.29 | 0.37 | 0.12 | 0.16 |
| **Q2** | 0.45 |  | 0.52 | 0.22 | 0.28 | 0.09 | 0.17 | 0.02 | -0.02 | 0.38 | 0.42 | 0.46 | 0.23 | 0.25 |
| **Q3** | 0.40 | 0.52 |  | 0.28 | 0.20 | 0.12 | 0.16 | 0.05 | -0.04 | 0.48 | 0.45 | 0.42 | 0.21 | 0.34 |
| **Q4** | 0.10 | 0.22 | 0.28 |  | 0.34 | 0.31 | 0.24 | 0.21 | 0.23 | 0.37 | 0.20 | 0.31 | 0.32 | 0.28 |
| **Q5** | 0.16 | 0.28 | 0.20 | 0.34 |  | 0.25 | 0.22 | 0.06 | 0.10 | 0.25 | 0.15 | 0.27 | 0.34 | 0.17 |
| **Q6** | 0.02 | 0.09 | 0.12 | 0.31 | 0.25 |  | 0.28 | 0.34 | 0.39 | 0.21 | 0.14 | 0.16 | 0.17 | 0.24 |
| **Q7** | 0.07 | 0.17 | 0.16 | 0.24 | 0.22 | 0.28 |  | 0.25 | 0.20 | 0.22 | 0.22 | 0.18 | 0.19 | 0.23 |
| **Q8** | -0.04 | 0.02 | 0.05 | 0.21 | 0.06 | 0.34 | 0.25 |  | 0.48 | 0.17 | 0.06 | 0.09 | 0.09 | 0.17 |
| **Q9** | -0.11 | -0.02 | -0.04 | 0.23 | 0.10 | 0.39 | 0.20 | 0.48 |  | 0.19 | 0.02 | 0.08 | 0.13 | 0.15 |
| **Q10** | 0.27 | 0.38 | 0.48 | 0.37 | 0.25 | 0.21 | 0.22 | 0.17 | 0.19 |  | 0.39 | 0.43 | 0.33 | 0.36 |
| **Q11** | 0.29 | 0.42 | 0.45 | 0.20 | 0.15 | 0.14 | 0.22 | 0.06 | 0.02 | 0.39 |  | 0.46 | 0.18 | 0.34 |
| **Q12** | 0.37 | 0.46 | 0.42 | 0.31 | 0.27 | 0.16 | 0.18 | 0.09 | 0.08 | 0.43 | 0.46 |  | 0.30 | 0.31 |
| **Q13** | 0.12 | 0.23 | 0.21 | 0.32 | 0.34 | 0.17 | 0.19 | 0.09 | 0.13 | 0.33 | 0.18 | 0.30 |  | 0.34 |
| **Q14** | 0.16 | 0.25 | 0.34 | 0.28 | 0.17 | 0.24 | 0.23 | 0.17 | 0.15 | 0.36 | 0.34 | 0.31 | 0.34 |  |

**Supplementary figure 16. Principle component analysis with varimax rotation for the compulsivity scale (CHIT)**

Top left – correlation matrix for the CHIT compulsivity scale items. Top right, task-component loadings after varimax rotation. Bottom – scree plot of eigenvalues. Source data are provided as a Source Data file.

**Supplementary table 41. Varimax rotated component loadings table for compulsivity scale (CHIT)**

| **Perfectionism** | **Reward drive** | **Cognitive rigidity** |  |
| --- | --- | --- | --- |
| **0.57** | -0.11 | 0.06 | Leaving tasks unfinished |
| **0.67** | -0.01 | 0.20 | Doing things just right |
| **0.71** | 0.04 | 0.16 | Completion to high standard |
| 0.22 | 0.29 | **0.48** | Repetitive thoughts |
| 0.17 | 0.08 | **0.54** | Habits |
| 0.06 | **0.51** | 0.28 | Addictive personality |
| 0.17 | **0.32** | 0.24 | Stubborn/rigid |
| 0.03 | **0.67** | 0.01 | Acting on urges |
| -0.08 | **0.70** | 0.11 | Immediate reward |
| **0.53** | 0.25 | 0.32 | Obsession with perfection |
| **0.61** | 0.10 | 0.13 | Higher standards than others |
| **0.59** | 0.11 | 0.30 | Soothed by completing tasks |
| 0.19 | 0.10 | **0.55** | Avoid uncontrolled situations |
| **0.36** | 0.24 | 0.30 | Need to be the best at things |

**Figure - data key**

Data are available for third party analysis via the UK Data Service. Downloadable as ‘COVID-19 impact dataset: Great British Intelligence Test, 2020’. Data and analyses from main text display items are available as follows.

Figure 1. Study schematic

- no data

Figure 2. Visual comparison of mental health and sleep measures during the Pre-UK Pandemic and Mid-UK Lockdown epochs

- Raw data - 01_raw_pre_and_mid_stage_data.mat

- Analyses - a01_MHcounts.mat

Figure 3. Modulation of differences in national mental health scores by population variables

- Raw data - 01_raw_pre_and_mid_stage_data.mat

- Analyses - a02_preVmid.mat & a03_preVmid_bydemographics.mat

- Estimates - Supplement 4

Figure 4. Individual item responses & principal component analysis for the PD-GIS at peak UK lockdown

- Raw data - 02_raw_mid_data_with_PDGIS.mat

- Analyses - a05_PDGIS_analysis.mat

- Estimates - Supplement 6

Figure 5. Interrelationships between the PD-GIS sub-scales and mental health assessment

- Raw data - 02_raw_mid_data_with_PDGIS.mat

Figure 6. Self-perceived pandemic impact by age

- Raw data - 02_raw_mid_data_with_PDGIS.mat

- Analyses - a05_PDGIS_analysis.mat

- Estimates - Supplement 8

Figure 7. Self-perceived pandemic impact by occupational status and cohabitees

- Raw data - 02_raw_mid_data_with_PDGIS.mat

- Analyses - a05_PDGIS_analysis.mat

- Estimates - Supplement 8

Figure 8. Self-perceived pandemic impact by outside space at home

- Raw data - 02_raw_mid_data_with_PDGIS.mat

- Analyses - a05_PDGIS_analysis.mat

- Estimates - Supplement 8

Figure 9. Self-perceived impact of the COVID-19 pandemic by pre-existing conditions

- Raw data - 02_raw_mid_data_with_PDGIS.mat

- Analyses - a05_PDGIS_analysis.mat

- Estimates - Supplement 8

Figure 10. Correlation of Trait and Technology with PD-GIS component scores

- Raw data - 02_raw_mid_data_with_PDGIS.mat

- Analyses - a05_PDGIS_analysis.mat

**Supplementary references**

1 Kroenke, K., Spitzer, R. L. & Williams, J. B. The Patient Health Questionnaire-2: validity of a two-item depression screener. *Med Care* **41**, 1284-1292, doi:10.1097/01.MLR.0000093487.78664.3C (2003).

2 Spitzer, R. L., Kroenke, K., Williams, J. B. & Lowe, B. A brief measure for assessing generalized anxiety disorder: the GAD-7. *Arch Intern Med* **166**, 1092-1097, doi:10.1001/archinte.166.10.1092 (2006).

3 John O.P, Naumann L.P & C.J., S. in *Handbook of personality: Theory and research* (ed R. W. Robins O. P. John, & L. A. Pervin ) (Guilford Press, 2008).

4 Chamberlain, S. R. & Grant, J. E. Initial validation of a transdiagnostic compulsivity questionnaire: the Cambridge-Chicago Compulsivity Trait Scale. *CNS Spectr* **23**, 340-346, doi:10.1017/S1092852918000810 (2018).

5 Albertella, L. *et al.* Compulsivity is measurable across distinct psychiatric symptom domains and is associated with familial risk and reward-related attentional capture. *CNS Spectr*, 1-8, doi:10.1017/S1092852919001330 (2019).

6 Horn, J. L. A rationale and test for the number of factors in factor analysis. *Psychometrika* (1965).
