# Supplementary material for: Associations between dimensions of behaviour, personality traits, and mental-health during the COVID-19 pandemic in the United Kingdom
Source: Nat Commun. 2021 Jul 16;12:4111. doi: 10.1038/s41467-021-24365-5 (PMC8285408; doi:10.1038/s41467-021-24365-5)
Supplement: Supplementary file 1 [file 41467_2021_24365_MOESM1_ESM.pdf]

Supplementary information: Hampshire et al.

Contents

Pages 2-4 **Supplementary methods** - Study Questionnaires

Pages 5-8 **Supplementary figures 1-8 & Supplementary table 1** - Comparison of demographic distributions in the pre-, early- and mid-pandemic datasets

Page 9 **Supplementary figure 9** – Analysis of day-by-day differences in mood self-assessment scores in January and May

Pages 10-16 **Supplementary tables 2-16** – Relationship between standard mental health score differences pre- to mid- pandemic lockdown and population factors

Page 17 **Supplementary results 1** - PD-GIS mental health sample bias analysis

Page 17 **Supplementary figure 10** - PD-GIS Principal Component Analysis

Page 18 **Supplementary figure 11** - PD-GIS vs Mental Health CCA train-test and sub sampling analysis

Page 19-34 **Supplementary tables 17-31** - Predicting individual differences in PD-GIS component scores from population variables

Pages 35-44 **Supplementary tables 32-41 & supplementary figures 12-16** - Principal Component Analyses of questionnaire scales.

Page 35 **Figure - data key**

Supplementary methods - Study Questionnaires

(a) Demographic and other contextual information

The following background information was collected from participants: age, gender, ethnicity, country of residence, level of education, occupational status, and income.

(b) Mood, anxiety, and sleep

Mood and anxiety symptoms were recorded using items from the extensively validated Patient Health Questionnaire 2 (PHQ-2) and GAD-7 respectively [1,2]The PHQ-2 and GAD-7 ask about symptoms over the preceding two weeks, and each question is answered on a 4-point scale, from 0 (not at all) to 3 (nearly every day). Additionally, we asked how many hours on average participants slept per night.

(c) Personality traits, and compulsivity

Personality traits were quantified using the extensively validated Big-5 Inventory, which comprises 44 questions[3]. Each question is a short phrase and is answered on a 5-point rating scale from 1 (strongly disagree) to 5 (strongly agree). Aspects of personality classically reflect extraversion, agreeableness, conscientiousness, neuroticism and openness to experience [3]Based on prior factor analysis of data from 60,000 participants, we used an abbreviated version, comprising 18 questions with a data-driven structure of 6 components. These are reported in the factor analysis in **Appendix 2**.

Compulsivity is a trans-diagnostic concept representing the tendency towards repetitive habits, and was measured using the Cambridge-Chicago Compulsivity Trait Scale (CHI-T)[4]. This is a 15-item questionnaire that is answered on a 4-point rating scale ranging from 1 (strongly disagree) to 4 (strongly agree). The CHI-T is sensitive to compulsivity across a range of disorders [4,5].

(d) Impact of the pandemic

The Pandemic General Impact Scale (PD-GIS) was developed specifically for the current study to quantify the self-perceived negative and positive impacts of the COVID-19 pandemic on daily life, as well as outlook, on multiple levels of psycho-socio-economic investigation. These pandemic-oriented measures can be juxtaposed against more generic scales, e.g., measuring of mental health symptoms. The PD-GIS was generated in response to the need for a scale that captured aspects of how people considered their daily lives to have been affected, that is, as opposed to more generic measures of their mental health status. The items were generated by the authors of this article, comprising psychiatrists, psychologists and neuroscientists. At a coarse grain, it was designed to have three main sub-sections. (1) Aspects of positive impact. (2) Aspects of negative impact. (3) Outlook. The exact wording of the scale was refined through multiple iterations by the researchers, who are experienced in developing new scales, with feedback from producers at BBC and BBC media, who are knowledgeable regarding the wording of questions such that people will be comfortable answering them online. Prior to application in the main study, the preliminary scale was deployed in ~1,000 participants as a pilot. On analysing the pilot data it was observed that there appeared to be multiple dimensions to the PD-GIS latent variable structure, that these were quite independent within the positive and negative domains, and that the item-component loadings were relatively simple and readily interpretable. Therefore, the full scale study included the scale as originally designed, so no items were removed.

The instrument comprises 47 questions, relating to potential negative and positive aspects of the situation, and longer-term outlook. Each item is answered on a 5-point scale ranging from 1 (strongly disagree) to 4 (strongly agree). Negative and positive impact items are generally couched in terms of how things have changed due to the pandemic and are contextualised by 'Please indicate how well the following statements describe the impact of the pandemic on you.' Negative impact questions were designed to cover areas of concern for health (own health and that of others), being concerned with the consequences of contracting COVID-19, loneliness, conflict at home, negative emotions from reading or listening to news, grieving, loss of employment, job or income, loss of leisure and wellbeing activities, loss of daily structure, disruption of sleep patterns, less healthy lifestyles, less focus on personal hygiene, loss of productivity, social disconnection, life being dominated by infection control routines, loss of important goods, medication or services, more arguments in the household, and going on the internet to avoid people at home. Positive impact questions were designed to cover less commuting time, more structure to the day, joy at being able to spent more time with people at home, more connections with people online, sense of shared community, more efficient or productive work, being more relaxed due to more time at

home, better sleep due to spare time, greater sense of purpose in work, greater opportunity to exercise, improved natural environment, time to read for pleasure, work less stressful due to doing it from home, spending more time on hobbies, spending less and saving money, more social contact outside of the home, feeling less tired, feeling better connected with people at home, more wildlife, taking greater appreciation for the simple things in life, and being less stressed by daily responsibilities.

Full-text for the PD-GIS is as follows:

Cue- Please indicate how well the following statements describe the impact of lockdown on you
I am more concerned about my personal health
I am more concerned about the health of my loved ones
I feel more lonely than before
There is an increased frequency or intensity of conflict at home
I am preoccupied with consequences of getting COVID-19
Watching or reading the news brings on unpleasant emotions, or distressing thoughts that are hard to get rid of
I have been grieving due to the loss of someone close to me
I have lost employment, job opportunities or income
I have lost leisure opportunities or activities important for my well-being
I feel that my daily routine no longer has enough structure
I have experienced changes in my sleep/wake patterns
My lifestyle and/or daily routine has become more unhealthy
I have not paid as much attention to my personal hygiene
My productivity has gone down
I have felt disconnected from important people in my life
Infection control routines more than ever dominate my life
I have lost access to essential goods, services or medication
I am arguing more often with the people I live with
I go online more to avoid the people I live with

I feel that I have more time as am commuting less
There is now more structure to my day
I am happier as am able to spend more time with people within my home
I am connecting online with people who I had trouble finding the time for before
There is a greater sense of shared community
I am working more efficiently/productively now
I feel more relaxed as am spending more time at home
I am sleeping better as have more spare time
I feel a greater sense of purpose in the work that I do
I am able to exercise more often
When I go outside, the environment is quieter and more relaxing than it was before
I have more time to read just for pleasure
I find work less stressful now that I am doing it from home
I am now spending more time on hobbies that I enjoy
I am spending less and saving more money than before
I have more social contact outside of my home
I feel less tired now
I am better connected now with the people I live with
There seems to be more wildlife now
I am enjoying the simple things in life more
I feel less stressed by my daily responsibilities

Cue - How will things change in the long term?
I believe the world will be a better place than it was
I believe the negative impact on the economy will be short lived
Things will change but not necessarily for the worse
I have more belief that we can cope with global problems like climate change
Technology science and healthcare will improve more rapidly than before

(e) Online Technology use

Technology use was quantified by asking about frequency of use of the following, over the previous 4 week period: Smart Phone, Computer (Desktop or Laptop), Tablet Device, Gaming Console, Email, Social Media, reading the news, playing computer games, online gambling, working, learning/studying, shopping, streaming films or music, and searching for information online. Each question was responded to on a 7-point scale, from 0 (never) to 7 (more often than hourly every day).

(f) Stress from online technology

Stress from online technology was measured by asking the participants the following questions, regarding the past 4 weeks: When you checked Email, did it tend to make you feel stressed/unhappy or relieved/happy? When you used social media, did it tend to make you feel stressed/unhappy or relieved/happy? When you read the news, did it tend to make you feel stressed/unhappy or relieved/happy? When you played computer games, did it tend to make you feel stressed/unhappy or relieved/happy? The response options for each question were: "Mostly stressed/unhappy", "Mostly relieved/happy", "Both", or "Neither".

(g) Maladaptive ('Addictive') use of online technology

Maladaptive use of online technology was quantified using the following questions, which were based on expert consensus amongst the study team in the field of Problematic Usage of the Internet: How often did you check email or social media accounts after you went to bed? How often did you use internet related activities to block out disturbing thoughts or soothe yourself? How often did you choose to spend time on internet related activities to battle loneliness or boredom? How often did you suffer from negative financial consequences because of an online activity? How often did you check your email or social media account or equivalent before something else that you needed to do? How often did you try to stop an excessive online activity but feel a compulsion to continue? How often did you try to cut down the amount of time you spend on-line and fail? The questions asked about these areas over the preceding 4-week period. For the first question (using technology before bed), response options were 1 (never) to 5 (daily). For the other questions, response options were: 1 (never) to 7 (more than hourly every day).

Comparison of demographic distributions in the pre, early and mid-pandemic datasets Proportions of participants in final analyses by population factor (Source data are provided as a Source Data file).

Supplementary figure 1. Age sample probability distributions

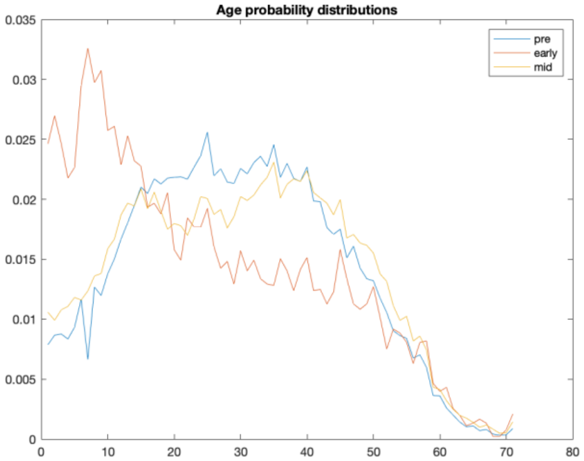

Proportion of participants per age year within the pre-, early- and mid-pandemic epochs. X axis is age, Y axis is proportion per epoch.

Supplementary figure 2. Sex sample probability distributions

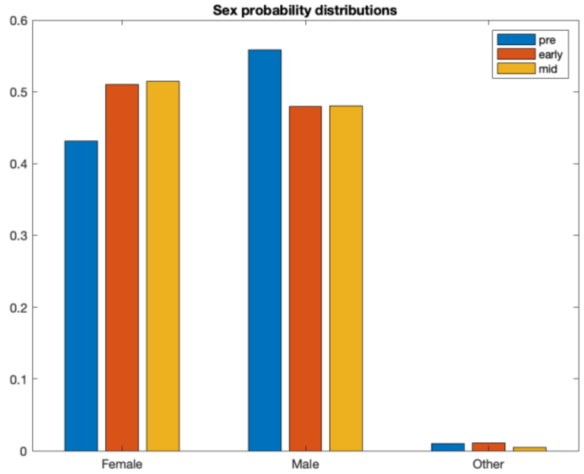

Proportion of participants per sex group (male, female or other) within the pre-, early- and mid-pandemic epochs. Y axis is proportion per epoch.

Supplementary figure 3. Handedness sample probability distributions

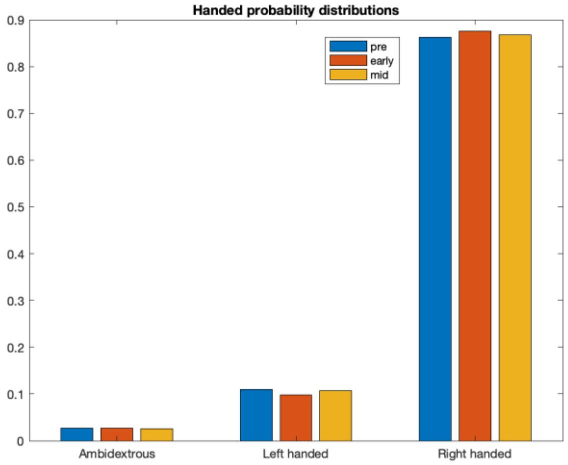

Proportion of participants per handedness group within the pre-, early- and mid-pandemic epochs. Y axis is proportion per epoch.

Supplementary figure 4. Education level sample probability distributions

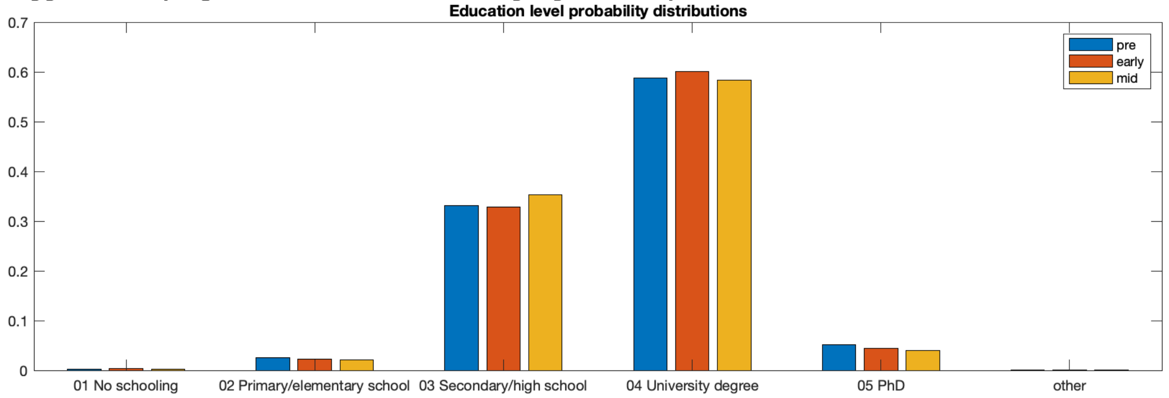

Proportion of participants per education level within the pre-, early- and mid-pandemic epochs. Y axis is proportion per epoch.

Supplementary figure 5. Occupational status sample probability distributions

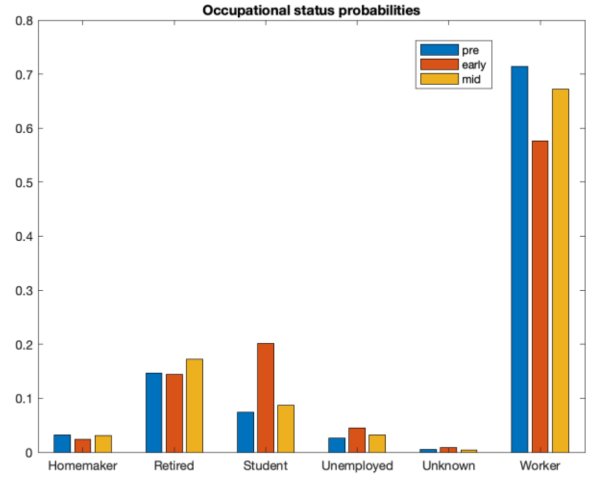

Proportion of participants per occupational status group within the pre-, early- and mid-pandemic epochs. Y axis is proportion per epoch.

Supplementary figure 6. First language sample probability distributions

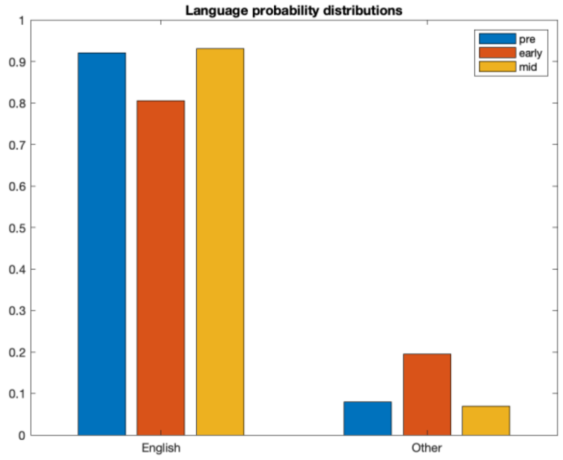

Proportion of participants per first language within the pre-, early- and mid-pandemic epochs. Y axis is proportion per epoch.

Supplementary figure 7 Country of residence sample probability distributions

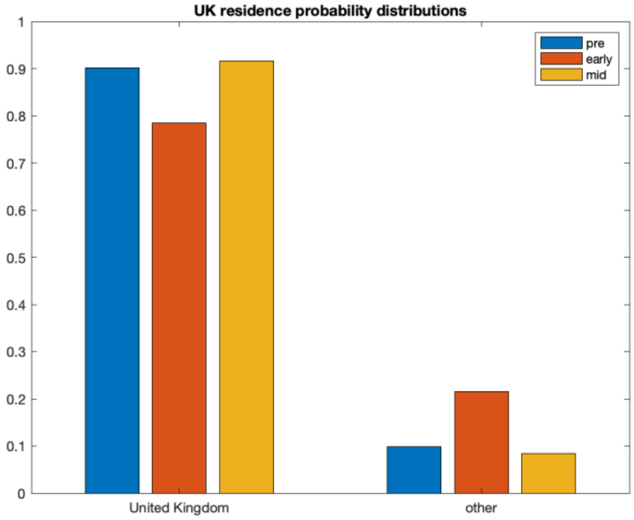

Proportion of participants per country of residence within the pre-, early- and mid-pandemic epochs. Y axis is proportion per epoch.

Supplementary figure 8 Earnings sample probability distributions

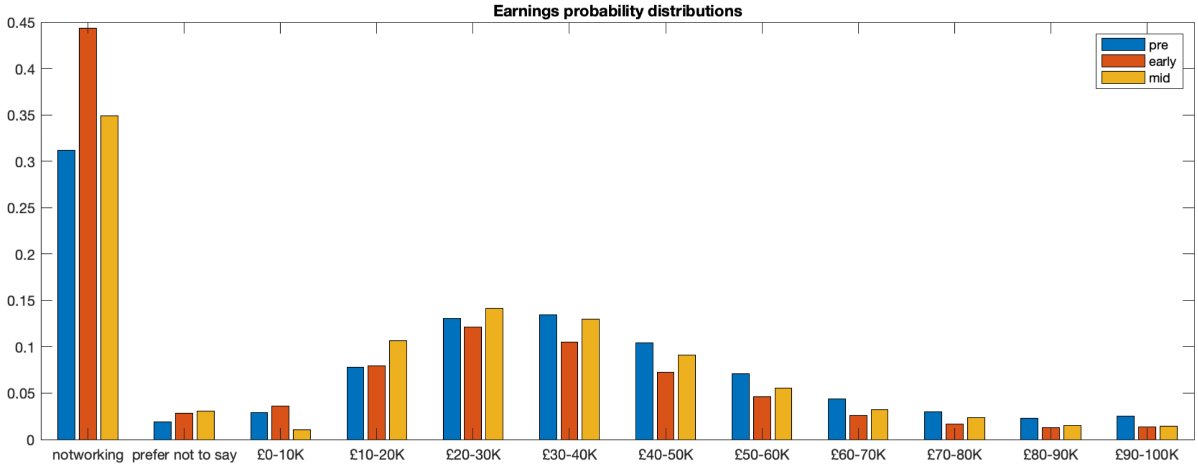

Proportion of participants per earnings bracket within the pre-, early- and mid-pandemic epochs. Y axis is proportion per epoch.

Supplementary table 1 Sample probability distributions for ethnicity within the pre-, early- and mid-pandemic epochs

Pre	Early	Mid	
0.838	0.759	0.897	White European or North American
0.045	0.102	0.014	East Asian
0.037	0.039	0.035	Indian, South Asian or South-East Asian
0.035	0.026	0.006	American Hispanic
0.023	0.034	0.025	Mixed ethnicity
0.011	0.017	0.015	Unknown/other
0.006	0.009	0.004	Sub-Saharan African or Afro-American
0.004	0.013	0.004	West-Central Asian
0.003	0.003	0.002	North African

Supplementary figure 9 – **Analysis of day-by-day mood self-assessment scores in January and May**

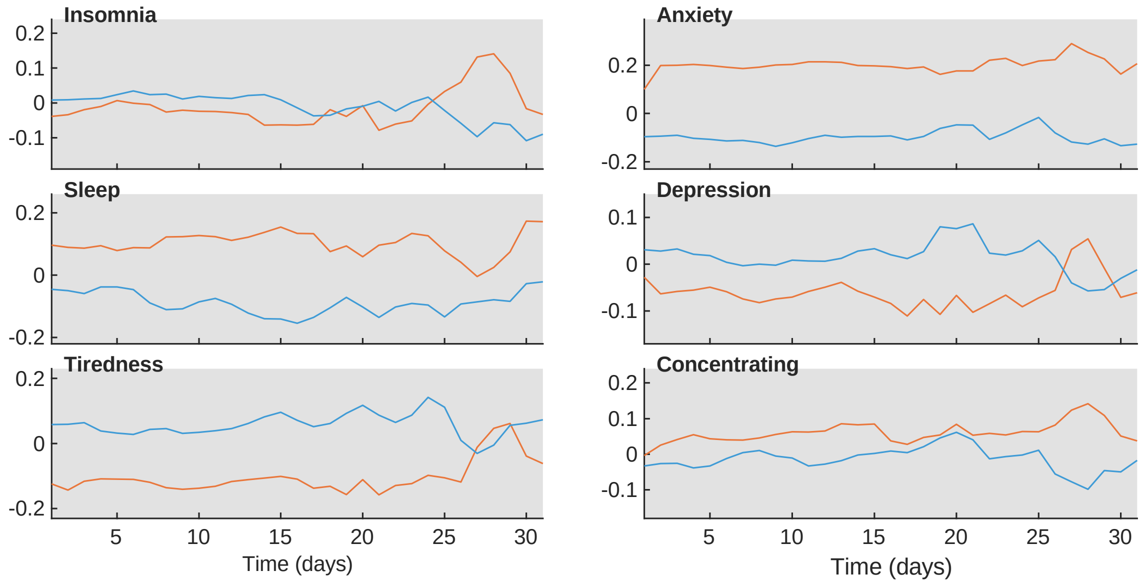

Mean scores for mood self-assessment measures were contrasted separately for each of 31 days after the two promotion launches on January 1st (blue, pre-pandemic) and May 2nd (orange, mid-lockdown). Demographic variables including age, sex, handedness, ethnicity, first language, country of residence, education level, employment status and earning have been factored out. Y axis is in standard deviation units. X axis is days since launch. Shading represents the standard error of the mean for data collected on that day. The overall pattern of differences can be seen to be consistent throughout these two months with increased anxiety, increased sleep, reduced tiredness, and similar mean levels of depression, insomnia and problems concentrating. Therefore, the observed differences in mental health measures reflect sustained differences throughout these epochs, that is, as opposed to transient spikes in national mood on individual days. Source data are provided as a Source Data file.

Relationship of mental health score differences pre- to mid- pandemic lockdown with population factors

Supplementary table 2 Anxiety – statistical significance of association between pre to mid pandemic difference with population variables

	DF	F	p
**age**	1	8683.70	<0.0001
**sex**	2	1943.50	<0.0001
**handedness**	2	3.52	0.0295
**first language**	1	45.67	<0.0001
**ethnic group**	9	26.87	<0.0001
**country of residence**	1	18.29	<0.0001
**education**	5	44.82	<0.0001
**occupational status**	5	701.29	<0.0001
**earnings**	12	97.43	<0.0001
**epoch**	1	7172.50	<0.0001
**age * epoch**	1	75.94	<0.0001
**sex * epoch**	2	152.25	<0.0001
**handedness * epoch**	2	3.18	0.0415
**first language * epoch**	1	1.85	0.1732
**ethnic group * epoch**	9	4.80	<0.0001
**country of residence * epoch**	1	4.34	0.0372
**education * epoch**	5	11.10	<0.0001
**occupational status * epoch**	5	24.80	<0.0001
**earnings * epoch**	11	4.14	<0.0001
**Error**	3.442E+05		

Supplementary table 3 Depression – statistical significance of association between pre to mid pandemic difference with population variables

	DF	F	p
**age**	1	6975.91	<0.0001
**sex**	2	451.96	<0.0001
**handedness**	2	0.89	0.4087
**first language**	1	9.57	0.0020
**ethnic group**	9	20.84	<0.0001
**country of residence**	1	11.86	0.0006
**education**	5	4.42	0.0005
**occupational status**	5	982.65	<0.0001
**earnings**	12	217.32	<0.0001
**epoch**	1	641.03	<0.0001
**age * epoch**	1	12.23	0.0005
**sex * epoch**	2	3.26	0.0385
**handedness * epoch**	2	0.45	0.6370
**first language * epoch**	1	2.33	0.1271
**ethnic group * epoch**	9	4.33	<0.0001
**country of residence * epoch**	1	4.90	0.0269
**education * epoch**	5	5.00	0.0001
**occupational status * epoch**	5	13.30	<0.0001
**earnings * epoch**	11	3.19	0.0002
**Error**	3.44E+05		

Supplementary table 4 Tiredness – statistical significance of association between pre to mid pandemic difference with population variables

	DF	F	p
age	1	6828.41	<0.0001
sex	2	1436.80	<0.0001
handedness	2	3.68	0.0253
first language	1	233.96	<0.0001
ethnic group	9	18.71	<0.0001
country of residence	1	8.79	0.0030
education	5	39.21	<0.0001
occupational status	5	1014.74	<0.0001
earnings	12	121.64	<0.0001
epoch	1	2193.40	<0.0001
age * epoch	1	0.24	0.6271
sex * epoch	2	15.13	<0.0001
handedness * epoch	2	3.42	0.0328
first language * epoch	1	0.72	0.3962
ethnic group * epoch	9	2.84	0.0024
country of residence * epoch	1	0.00	0.9528
education * epoch	5	4.08	0.0010
occupational status * epoch	5	2.72	0.0184
earnings * epoch	11	3.72	<0.0001
Error	3.44E+05		

Supplementary table 5 Trouble concentrating – statistical significance of association between pre to mid pandemic difference with population variables

	DF	F	p
age	1	8034.33	<0.0001
sex	2	145.74	<0.0001
handedness	2	2.05	0.1290
first language	1	30.00	<0.0001
ethnic group	9	13.63	<0.0001
country of residence	1	0.28	0.5993
education	5	5.80	<0.0001
occupational status	5	707.83	<0.0001
earnings	12	81.65	<0.0001
epoch	1	550.19	<0.0001
age * epoch	1	2.34	0.1259
sex * epoch	2	3.92	0.0199
handedness * epoch	2	1.40	0.2456
first language * epoch	1	2.16	0.1412
ethnic group * epoch	9	5.18	<0.0001
country of residence * epoch	1	0.36	0.5476
education * epoch	5	4.14	0.0009
occupational status * epoch	5	10.83	<0.0001
earnings * epoch	11	1.54	0.1097
Error	3.44E+05		

Supplementary table 6 Insomnia – statistical significance of association between pre to mid pandemic difference with population variables

	DF	F	p
**age**	1	395.76	<0.0001
**sex**	2	885.26	<0.0001
**handedness**	2	6.36	0.0017
**first language**	1	471.69	<0.0001
**ethnic group**	9	14.87	<0.0001
**country of residence**	1	4.41	0.0357
**education**	5	74.51	<0.0001
**occupational status**	5	521.86	<0.0001
**earnings**	12	63.87	<0.0001
**epoch**	1	18.22	<0.0001
**age * epoch**	1	24.19	<0.0001
**sex * epoch**	2	0.36	0.6948
**handedness * epoch**	2	1.71	0.1817
**first language * epoch**	1	9.24	0.0024
**ethnic group * epoch**	9	2.06	0.0294
**country of residence * epoch**	1	2.37	0.1240
**education * epoch**	5	2.39	0.0356
**occupational status * epoch**	5	2.27	0.0449
**earnings * epoch**	11	2.66	0.0020
**Error**	3.44E+05		

Supplementary table 7 Hours slept – statistical significance of association between pre to mid pandemic difference with population variables

	DF	F	p
**age**	1	3985.93	<0.0001
**sex**	2	39.91	<0.0001
**handedness**	2	38.85	<0.0001
**first language**	1	0.63	0.4273
**ethnic group**	9	75.53	<0.0001
**country of residence**	1	11.35	0.0008
**education**	5	213.37	<0.0001
**occupational status**	5	215.77	<0.0001
**earnings**	12	9.45	<0.0001
**epoch**	1	1397.34	<0.0001
**age * epoch**	1	197.47	<0.0001
**sex * epoch**	2	0.33	0.7163
**handedness * epoch**	2	0.17	0.8431
**first language * epoch**	1	14.97	0.0001
**ethnic group * epoch**	9	3.07	0.0011
**country of residence * epoch**	1	8.69	0.0032
**education * epoch**	5	0.76	0.5782
**occupational status * epoch**	5	20.49	<0.0001
**earnings * epoch**	11	1.12	0.3438
**Error**	3.44E+05		

Supplementary table 8 Differences in mean mid-pandemic minus pre-pandemic mental health scores by age (SD units)

AGE	Anxiety	Depression	Tiredness	Problems concentrating	Insomnia	Hours Slept
16	0.09	-0.17	-0.15	0.03	0.07	0.49
17	0.17	-0.05	-0.03	0.17	0.15	0.41
18	0.10	-0.14	-0.16	0.03	0.07	0.44
19	0.10	-0.14	-0.09	0.06	-0.03	0.42
20	0.15	-0.15	-0.15	0.05	-0.02	0.26
21	0.13	-0.17	-0.14	0.04	0.01	0.29
22	0.22	-0.10	-0.06	0.08	0.03	0.36
23	0.24	-0.12	-0.09	0.08	0.08	0.21
24	0.20	-0.15	-0.17	0.06	-0.02	0.28
25	0.22	-0.09	-0.13	0.08	0.04	0.25
26	0.24	-0.08	-0.16	0.10	0.07	0.25
27	0.23	-0.10	-0.14	0.12	0.00	0.26
28	0.21	-0.14	-0.14	0.05	-0.01	0.30
29	0.23	-0.10	-0.13	0.09	0.06	0.19
30	0.23	-0.12	-0.17	0.06	0.00	0.21
31	0.26	-0.11	-0.17	0.08	0.04	0.21
32	0.28	-0.10	-0.17	0.08	0.00	0.18
33	0.30	-0.07	-0.17	0.11	0.03	0.11
34	0.26	-0.09	-0.16	0.06	-0.01	0.13
35	0.29	-0.05	-0.17	0.06	0.02	0.13
36	0.27	-0.06	-0.15	0.08	0.01	0.14
37	0.27	-0.08	-0.15	0.11	-0.01	0.14
38	0.30	-0.05	-0.11	0.13	0.04	0.09
39	0.33	-0.05	-0.12	0.10	0.00	0.13
40	0.33	-0.08	-0.16	0.04	0.01	0.13
41	0.27	-0.05	-0.15	0.09	0.01	0.07
42	0.31	-0.05	-0.13	0.09	0.03	0.07
43	0.30	-0.08	-0.13	0.06	-0.01	0.06
44	0.28	-0.09	-0.17	0.02	-0.02	0.09
45	0.29	-0.05	-0.12	0.10	-0.01	0.13
46	0.29	-0.09	-0.16	0.03	-0.06	0.12
47	0.29	-0.10	-0.14	0.05	-0.02	0.12
48	0.27	-0.07	-0.14	0.07	-0.02	0.13
49	0.25	-0.11	-0.19	0.06	-0.08	0.17
50	0.32	-0.04	-0.14	0.09	0.00	0.08
51	0.32	-0.07	-0.12	0.09	-0.01	0.07
52	0.30	-0.08	-0.12	0.09	0.00	0.09
53	0.31	-0.07	-0.13	0.09	0.00	0.11
54	0.29	-0.06	-0.11	0.06	0.01	0.07

**55**	0.31	-0.06	-0.16	0.06	0.00	0.05
**56**	0.31	-0.08	-0.12	0.05	-0.02	0.10
**57**	0.36	-0.01	-0.12	0.08	-0.01	0.11
**58**	0.35	-0.03	-0.13	0.10	0.00	0.03
**59**	0.36	-0.02	-0.09	0.16	0.00	0.02
**60**	0.34	-0.03	-0.12	0.11	0.01	0.06
**61**	0.39	-0.03	-0.16	0.07	0.00	0.01
**62**	0.36	-0.02	-0.12	0.11	-0.05	0.06
**63**	0.39	0.01	-0.09	0.14	0.00	0.03
**64**	0.42	0.04	-0.06	0.14	0.02	-0.03
**65**	0.43	0.04	-0.09	0.17	0.01	0.01
**66**	0.39	0.03	-0.07	0.15	-0.04	-0.02
**67**	0.40	0.04	-0.10	0.16	-0.03	-0.02
**68**	0.41	0.05	-0.07	0.17	-0.02	-0.02
**69**	0.32	-0.02	-0.14	0.09	-0.03	0.02
**70**	0.40	0.01	-0.09	0.13	-0.03	-0.04
**71**	0.43	0.07	-0.05	0.16	0.04	-0.06
**72**	0.38	0.02	-0.13	0.10	0.01	-0.06
**73**	0.39	0.01	-0.13	0.10	-0.09	0.05
**74**	0.43	0.04	-0.03	0.17	0.01	-0.01
**75**	0.31	-0.02	-0.16	0.13	-0.02	0.01
**76**	0.43	0.07	-0.03	0.08	0.03	-0.09
**77**	0.40	0.09	-0.02	0.11	0.04	-0.04
**78**	0.28	-0.11	-0.13	0.05	-0.05	-0.05
**79**	0.27	-0.08	0.00	0.06	0.04	-0.04
**80**	0.39	0.05	-0.13	0.05	0.00	-0.02
**81**	0.44	0.13	-0.05	0.07	-0.16	0.02
**82**	0.38	0.10	-0.15	0.19	-0.05	0.19
**83**	0.53	0.18	-0.08	0.25	0.14	-0.05
**84**	0.40	0.33	0.03	0.27	-0.01	-0.15
**85**	0.43	0.18	-0.18	0.01	-0.08	0.15
**86**	0.29	-0.02	-0.15	-0.01	-0.14	0.19

Supplementary table 9 Differences in mean mid- minus pre-pandemic mental health scores by gender (SD units)

AGE	Anxiety	Depression	Tiredness	Problems concentrating	Insomnia	Hours Slept
Female	0.33	-0.08	-0.17	0.08	0.00	0.12
Male	0.22	-0.07	-0.14	0.08	-0.01	0.14
Other	0.39	0.07	-0.02	0.22	0.07	0.22

Supplementary table 10 Differences in mean mid- minus pre-pandemic mental health scores by handedness (SD units)

Handed	Anxiety	Depression	Tiredness	Problems concentrating	Insomnia	Hours Slept
Ambidextrous	0.23	-0.05	-0.08	0.10	0.04	0.12
Left	0.30	-0.07	-0.14	0.07	0.00	0.14
Right	0.29	-0.07	-0.14	0.08	0.01	0.14

Supplementary table 11 Differences in mean mid- minus pre-pandemic mental health scores by 1st language (SD units)

1st language	Anxiety	Depression	Tiredness	Problems concentrating	Insomnia	Hours Slept
English	0.29	-0.06	-0.14	0.09	0.01	0.13
Other	0.23	-0.13	-0.17	0.03	-0.03	0.21

Supplementary table 12 Differences in mean mid- minus pre-pandemic mental health scores by ethnic group (SD units)

	Anxiety	Depression	Tiredness	Problems concentrating	Insomnia	Hours Slept
American Hispanic	0.16	-0.20	-0.28	0.00	-0.08	0.13
Mixed	0.22	-0.07	-0.09	0.06	0.00	0.19
African	0.19	-0.13	-0.15	0.03	0.03	0.29
Asian	0.21	-0.09	-0.18	0.06	0.02	0.18
Other/unknown	0.27	-0.07	-0.11	0.09	0.06	0.10
White	0.30	-0.06	-0.13	0.09	0.01	0.13

Supplementary table 13 Differences in mean mid- minus pre-pandemic mental health scores by country of residence (SD units)

	Anxiety	Depression	Tiredness	Problems concentrating	Insomnia	Hours Slept
UK	0.29	-0.06	-0.14	0.09	0.00	0.14
Other	0.24	-0.11	-0.14	0.05	0.03	0.09

Supplementary table 14 Differences in mean mid- minus pre-pandemic mental health scores by education (SD units)

	Anxiety	Depression	Tiredness	Problems concentrating	Insomnia	Hours Slept
No school	0.34	0.15	0.09	0.22	0.09	0.18
Primary/elementary	0.22	-0.03	-0.14	0.10	0.02	0.05
Secondary/high	0.26	-0.05	-0.13	0.08	0.02	0.12
Degree	0.31	-0.07	-0.15	0.08	0.00	0.15
PhD	0.31	-0.09	-0.13	0.10	-0.01	0.12

Supplementary table 15 Differences in mean mid- minus pre-pandemic mental health scores by occupational status (SD units)

	Anxiety	Depression	Tiredness	Problems concentrating	Insomnia	Hours Slept
Disabled	0.01	-0.12	-0.06	-0.06	-0.07	0.00
Homemaker	0.31	-0.07	-0.13	0.05	-0.02	0.02
Retired	0.38	0.02	-0.09	0.13	-0.02	-0.01
Student	0.18	-0.11	-0.11	0.07	0.04	0.37
Unemployed	0.16	-0.14	-0.10	0.07	-0.04	0.11
Worker	0.29	-0.08	-0.15	0.08	0.01	0.14

Supplementary table 16 Differences in mean mid- minus pre-pandemic mental health scores by earnings (SD units)

	Anxiety	Depression	Tiredness	Problems concentrating	Insomnia	Hours Slept
Not saying	0.38	-0.01	-0.04	0.09	0.08	0.15
Not working	0.28	-0.05	-0.11	0.09	-0.01	0.11
£0-10K	0.32	0.01	-0.10	0.13	0.00	0.19
£10-20K	0.26	-0.10	-0.17	0.06	0.03	0.12
£20-30K	0.27	-0.09	-0.16	0.07	0.01	0.16
£30-40K	0.27	-0.10	-0.18	0.07	0.00	0.14
£40-50K	0.26	-0.12	-0.20	0.05	-0.03	0.15
£50-60K	0.26	-0.12	-0.20	0.05	-0.03	0.15
£60-70K	0.29	-0.10	-0.18	0.04	-0.03	0.12
£70-80K	0.29	-0.10	-0.15	0.07	-0.06	0.11
£80-90K	0.28	-0.11	-0.13	0.06	-0.06	0.16
£90-100K	0.29	-0.09	-0.16	0.07	0.01	0.08
>100K	0.32	-0.06	-0.15	0.11	-0.01	0.14

Supplementary results 1 – **PD-GIS mental health sampling bias analysis**

One concern could be that people who opt to answer Cornonavirus-19 questionnaires are those for whom it is more relevant, e.g., due to their mental health status. To address this issue, we quantified sampling bias for the optional self-perceived impact sub-scale by analysing differences in mood measures for participants who did (79,736) minus did not (112046) opt to complete the PD-GIS. Differences in anxiety (0.018SDs t=2.0247 p=0.043), depression (-0.037SDs t=-4.4958 p<0.001), concentration (-0.073 SDs t=-8.4393 p<0.001), insomnia (0.032SDs t=3.4731 p<0.001), hours slept (-0.058SDs t=-7.1141 p<0.001) and tiredness (0.035SDs t=4.0017 p<0.001) scores were statistically significant but bi-directional with respect to valence, and critically, of negligible effect size scale. This accords poorly with the possibility of sampling bias towards people for whom mental health problems are most relevant during the pandemic in the context of PD-GIS analysis.

Supplementary figure 10 – **PCA analysis of the PD-GIS items.**

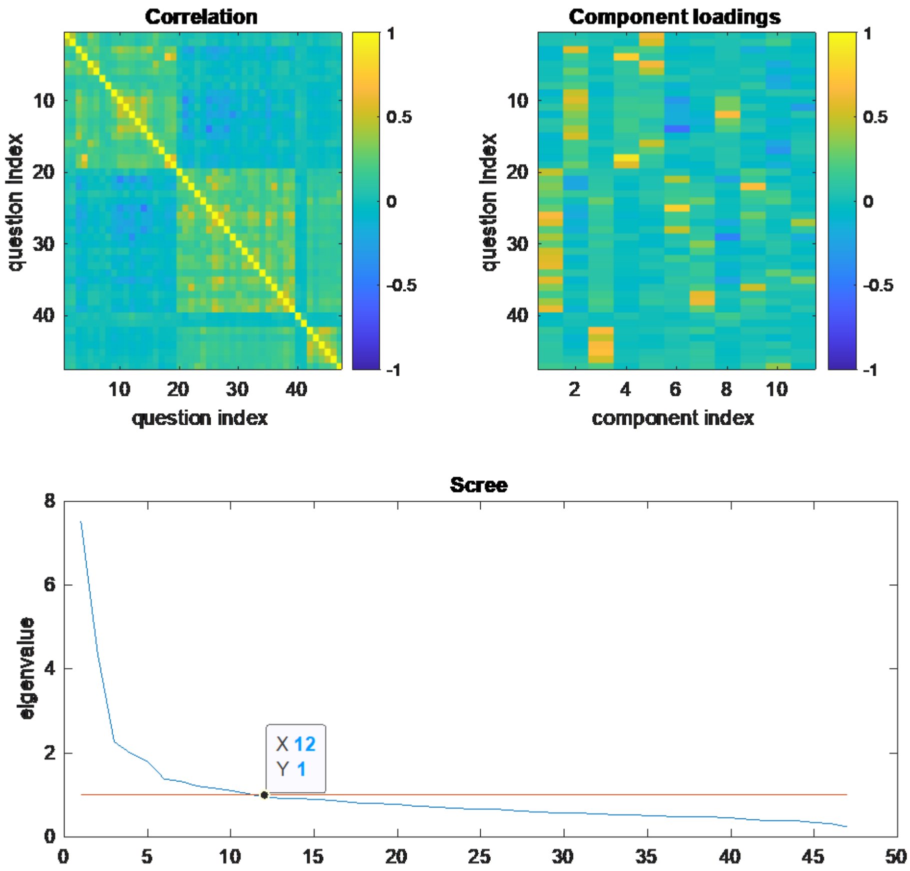

A MATLAB implementation of Horn's Parallel Analysis[6] was applied to estimate the number of significant components from the principal component analysis. PCA. This is a permutation-based approach whereby the true data are permuted and data reduced with PCA many times, producing distributions of variance explained by components at each index for statistical comparison to those observed for the unpermuted data. Estimated with 1000 permutations indicated 7 statistically significant components (greater than 95% of values within the corresponding null distribution) when the PD-GIS data were analysed in this manner. Application of the Kaiser convention would indicate 11 components with eigenvalues >1. **Top left**, cross correlation matrix for PD-GIS items. **Top right**, question-component loadings after varimax rotation. **Bottom**, scree plot. Note 11th components places above the scree and prior to the 4th inflection point. (All data and models are available for download from the UK Data Service). Source data are provided as a Source Data file.

Supplementary figure 11 – Sub sampling Train-Test pipeline to evaluate overfit in the PD-GIS by Mental Health Canonical Correlation analysis

Pandemic General Impact Scale	Mental health assessment: Nervous, anxious or on edge	Can't stop worrying	Worry about different things	Trouble relaxing	Restless, can't sit still	Annoyed/irritable	Afraid something awful might happen	Little pleasure doing things	Down or depressed	Tired/ little energy	Trouble concentrating	Can't get to sleep or stay asleep	Hrs slept per night	Canonical r	p
More time, less stressed/tired	-0.11	-0.09	-0.09	-0.15	-0.07	-0.10	-0.06	-0.10	-0.12	-0.17	-0.07	-0.18	0.23	0.56	p<0.0001
Disrupted lifestyle	0.25	0.23	0.23	0.24	0.20	0.21	0.18	0.32	0.32	0.29	0.29	0.23	0.02	0.33	p<0.0001
Health concerns	0.29	0.30	0.30	0.27	0.18	0.14	0.36	0.17	0.23	0.20	0.19	0.20	-0.12	0.26	p<0.0001
Positive outlook	-0.05	-0.04	-0.03	-0.03	0.00	-0.05	-0.04	-0.04	-0.05	0.00	-0.02	-0.01	0.01	0.23	p<0.0001
Conflict at home	0.13	0.12	0.12	0.12	0.11	0.31	0.10	0.15	0.16	0.11	0.12	0.05	0.02	0.15	p<0.0001
Improved environment	0.03	0.01	0.02	0.02	-0.01	0.01	0.00	-0.11	-0.05	-0.05	-0.05	0.02	-0.03	0.12	p<0.0001
More time for people	0.06	0.04	0.06	0.08	0.05	0.12	0.06	0.01	-0.02	0.12	0.04	0.07	-0.06	0.04	p<0.0001

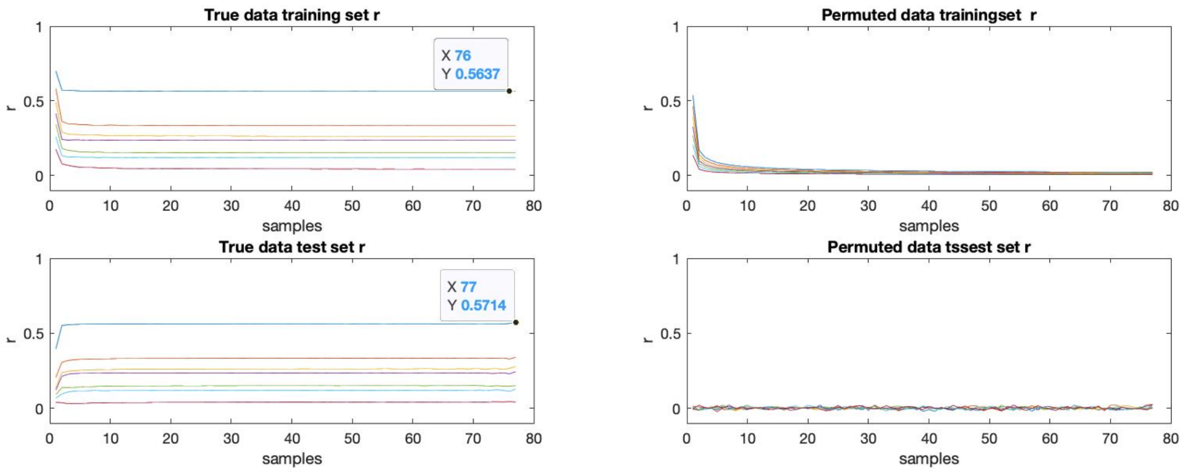

Upper panel left. Bivariate Pearson's correlations between PD-GIS component scores and scores on Mood Self-Assessment items. Upper panel right. Canonical Correlation mode scores. Middle left. CCA mode scores for trained data sub sampling at different sizes (X axis is in thousands and Y axis is mode correlation value). Middle right, the same analyses conducted for data where the index of the X matrix was permuted, breaking the X-Y matrix linkage whilst retaining their inner structure. Note the near zero scores above 20K samples, indicating little overfit. Bottom left. Mode correlation scores when applying the trained CCA model to the held-out data, to which the model was naïve, with X axis corresponding to the number of participants in the trained set, whereby the held-out set comprises all other participants. Note that canonical r values approximate those of the trained set at higher sample size, indicating little overfit. Bottom right. The analysis of held-out data repeated for the permutated data. Source data are provided as a Source Data file.

Predicting individual differences in PD-GIS component scores from population variables

Supplementary table 17. PD-GIS by sociodemographic factors ANOVA. More time, less stressed & tired

		SumSq	DF	MeanSq	F	pValue
Demographics	Age	187	1	187	164	<0.0001
	Sex	124	2	62	55	<0.0001
	Handedness	16	2	8	7	0.0007
	First language	18	1	18	16	<0.0001
	Ethnicity	152	5	30	27	<0.0001
	Country of residence	1	1	1	1	0.2809
	Education	192	4	48	42	<0.0001
	Relationship status	24	5	5	4	0.0007
	Home type	3	6	0	0	0.8628
	Work arrangements	4296	11	391	343	<0.0001
	Income negatively affected	135	1	135	119	<0.0001
Cohabitees	adult children	1	1	1	1	0.3620
	school children	113	1	113	100	<0.0001
	housemates	1	1	1	1	0.3660
	friends	2	1	2	2	0.2118
	grandparents	2	1	2	2	0.1765
	Home schooled children	48	1	48	42	<0.0001
	Inlaws	0	1	0	0	0.7917
	Alone	1	1	1	1	0.3757
	Parents	6	1	6	5	0.0252
	Partner	1	1	1	1	0.2769
	Preschool children	841	1	841	739	<0.0001
Outside space	Balcony	3	1	3	3	0.1007
	Large garden	6	1	6	5	0.0191
	None	18	1	18	16	<0.0001
	Overlooked	51	1	51	45	<0.0001
	Private	8	1	8	7	0.0079
	Relaxing	132	1	132	116	<0.0001
	Small garden	2	1	2	1	0.2336
	unpleasant	98	1	98	86	<0.0001
	Error	86370	75919	1		

Supplementary table 18. PD-GIS by sociodemographic factors ANOVA. Disrupted lifestyle

		SumSq	DF	MeanSq	F	pValue
Demographics	Age	1752	1	1752	1711	<0.0001
	Sex	332	2	166	162	<0.0001
	Handedness	24	2	12	12	<0.0001
	First language	328	1	328	321	<0.0001
	Ethnicity	15	5	3	3	0.0120
	Country of residence	1	1	1	1	0.3901
	Education	8	4	2	2	0.0834
	Relationship status	78	5	16	15	<0.0001
	Home type	59	6	10	10	<0.0001
	Work arrangements	2846	11	259	253	<0.0001
	Income negatively affected	1321	1	1321	1291	<0.0001
Cohabitees	adult children	9	1	9	9	0.0025
	school children	10	1	10	10	0.0018
	housemates	40	1	40	40	<0.0001
	friends	4	1	4	4	0.0553
	grandparents	1	1	1	1	0.4349
	Home schooled children	1	1	1	1	0.3356
	Inlaws	3	1	3	3	0.1037
	Alone	83	1	83	81	<0.0001
	Parents	7	1	7	7	0.0081
	Partner	4	1	4	4	0.0396
	Preschool children	21	1	21	21	<0.0001
Outside space	Balcony	1	1	1	1	0.3100
	Large garden	0	1	0	0	0.4879
	None	46	1	46	45	<0.0001
	Overlooked	107	1	107	105	<0.0001
	Private	1	1	1	1	0.3443
	Relaxing	32	1	32	31	<0.0001
	Small garden	13	1	13	13	0.0004
	unpleasant	108	1	108	106	<0.0001
	Error	77712	75919	1		

Supplementary table 19. PD-GIS by sociodemographic factors ANOVA. Health concerns

Demographics	Age	144	1	144	109	<0.0001
	Sex	1714	2	857	644	<0.0001
	Handedness	3	2	2	1	0.3173
	First language	96	1	96	72	<0.0001
	Ethnicity	141	5	28	21	<0.0001
	Country of residence	2	1	2	2	0.1872
	Education	78	4	19	15	<0.0001
	Relationship status	26	5	5	4	0.0016
	Home type	71	6	12	9	<0.0001
	Work arrangements	549	11	50	37	<0.0001
	Income negatively affected	258	1	258	194	<0.0001
Cohabitees	adult children	77	1	77	58	<0.0001
	school children	179	1	179	135	<0.0001
	housemates	7	1	7	6	0.0181
	friends	4	1	4	3	0.0795
	grandparents	15	1	15	11	0.0010
	Home schooled children	24	1	24	18	<0.0001
	Inlaws	0	1	0	0	0.7597
	Alone	9	1	9	7	0.0080
	Parents	43	1	43	32	<0.0001
	Partner	17	1	17	13	0.0004
	Preschool children	0	1	0	0	0.6905
Outside space	Balcony	0	1	0	0	0.7740
	Large garden	54	1	54	41	<0.0001
	None	8	1	8	6	0.0137
	Overlooked	62	1	62	47	<0.0001
	Private	2	1	2	2	0.2090
	Relaxing	48	1	48	36	<0.0001
	Small garden	4	1	4	3	0.0985
	unpleasant	31	1	31	23	<0.0001
	Error	101030	75919	1		

Supplementary table 20. PD-GIS by sociodemographic factors ANOVA. Positive outlook

Demographics	Age	7	1	7	5	0.0228
	Sex	684	2	342	261	<0.0001
	Handedness	5	2	3	2	0.1294
	First language	75	1	75	58	<0.0001
	Ethnicity	206	5	41	31	<0.0001
	Country of residence	3	1	3	2	0.1456
	Education	480	4	120	92	<0.0001
	Relationship status	48	5	10	7	<0.0001
	Home type	16	6	3	2	0.0521
	Work arrangements	199	11	18	14	<0.0001
	Income negatively affected	415	1	415	317	<0.0001
Cohabitees	adult children	3	1	3	2	0.1575
	school children	30	1	30	23	<0.0001
	housemates	2	1	2	1	0.2545
	friends	0	1	0	0	0.9235
	grandparents	9	1	9	7	0.0098
	Home schooled children	18	1	18	14	0.0002
	Inlaws	0	1	0	0	0.7003
	Alone	15	1	15	11	0.0007
	Parents	0	1	0	0	0.6248
	Partner	0	1	0	0	0.8729
	Preschool children	22	1	22	17	<0.0001
Outside space	Balcony	0	1	0	0	0.5687
	Large garden	2	1	2	1	0.2540
	None	5	1	5	4	0.0511
	Overlooked	18	1	18	13	0.0002
	Private	0	1	0	0	0.6835
	Relaxing	17	1	17	13	0.0003
	Small garden	0	1	0	0	0.6249
	unpleasant	25	1	25	19	<0.0001
	Error	99354	75919	1		

Supplementary table 21. PD-GIS by sociodemographic factors ANOVA. Increased conflict at home

Demographics	Age	147	1	147	132	<0.0001
	Sex	12	2	6	5	0.0046
	Handedness	5	2	2	2	0.1217
	First language	4	1	4	4	0.0469
	Ethnicity	7	5	1	1	0.2928
	Country of residence	1	1	1	1	0.4715
	Education	13	4	3	3	0.0220
	Relationship status	96	5	19	17	<0.0001
	Home type	30	6	5	5	0.0001
	Work arrangements	119	11	11	10	<0.0001
	Income negatively affected	42	1	42	38	<0.0001
Cohabitees	adult children	86	1	86	77	<0.0001
	school children	756	1	756	681	<0.0001
	housemates	0	1	0	0	0.8502
	friends	0	1	0	0	0.8406
	grandparents	1	1	1	1	0.4107
	Home schooled children	334	1	334	301	<0.0001
	Inlaws	24	1	24	21	<0.0001
	Alone	36	1	36	32	<0.0001
	Parents	430	1	430	388	<0.0001
	Partner	17	1	17	15	<0.0001
	Preschool children	424	1	424	382	<0.0001
Outside space	Balcony	1	1	1	1	0.3852
	Large garden	1	1	1	1	0.2929
	None	1	1	1	1	0.4234
	Overlooked	5	1	5	4	0.0405
	Private	0	1	0	0	0.6200
	Relaxing	47	1	47	42	<0.0001
	Small garden	0	1	0	0	0.7396
	unpleasant	13	1	13	11	0.0007
	Error	84236	75919	1		

Supplementary table 22. PD-GIS by sociodemographic factors ANOVA. Improved environment

Demographics	Age	73	1	73	46	<0.0001
	Sex	2623	2	1312	822	<0.0001
	Handedness	45	2	22	14	<0.0001
	First language	186	1	186	117	<0.0001
	Ethnicity	222	5	44	28	<0.0001
	Country of residence	62	1	62	39	<0.0001
	Education	353	4	88	55	<0.0001
	Relationship status	149	5	30	19	<0.0001
	Home type	51	6	9	5	<0.0001
	Work arrangements	601	11	55	34	<0.0001
	Income negatively affected	14	1	14	9	0.0027
Cohabitees	adult children	4	1	4	3	0.0992
	school children	0	1	0	0	0.8859
	housemates	0	1	0	0	0.7590
	friends	6	1	6	4	0.0482
	grandparents	4	1	4	3	0.1056
	Home schooled children	1	1	1	1	0.4578
	Inlaws	1	1	1	1	0.4747
	Alone	20	1	20	13	0.0004
	Parents	3	1	3	2	0.1986
	Partner	3	1	3	2	0.1930
	Preschool children	9	1	9	5	0.0194
Outside space	Balcony	14	1	14	9	0.0035
	Large garden	63	1	63	40	<0.0001
	None	38	1	38	24	<0.0001
	Overlooked	38	1	38	24	<0.0001
	Private	12	1	12	8	0.0058
	Relaxing	237	1	237	148	<0.0001
	Small garden	11	1	11	7	0.0092
	unpleasant	96	1	96	60	<0.0001
	Error	121070	75919	2		

Supplementary table 23. PD-GIS by sociodemographic factors ANOVA. More time for people

Demographics	Age	916	1	916	698	<0.0001
	Sex	73	2	37	28	<0.0001
	Handedness	3	2	2	1	0.2765
	First language	36	1	36	27	<0.0001
	Ethnicity	77	5	15	12	<0.0001
	Country of residence	0	1	0	0	0.9749
	Education	11	4	3	2	0.0755
	Relationship status	129	5	26	20	<0.0001
	Home type	79	6	13	10	<0.0001
	Work arrangements	1700	11	155	118	<0.0001
	Income negatively affected	42	1	42	32	<0.0001
Cohabitees	adult children	250	1	250	191	<0.0001
	school children	1486	1	1486	1133	<0.0001
	housemates	460	1	460	351	<0.0001
	friends	14	1	14	11	0.0012
	grandparents	6	1	6	4	0.0387
	Home schooled children	811	1	811	618	<0.0001
	Inlaws	11	1	11	8	0.0037
	Alone	1682	1	1682	1282	<0.0001
	Parents	172	1	172	131	<0.0001
	Partner	164	1	164	125	<0.0001
	Preschool children	1435	1	1435	1094	<0.0001
Outside space	Balcony	1	1	1	1	0.3519
	Large garden	20	1	20	16	<0.0001
	None	1	1	1	1	0.3502
	Overlooked	34	1	34	26	<0.0001
	Private	1	1	1	1	0.3832
	Relaxing	35	1	35	27	<0.0001
	Small garden	13	1	13	10	0.0017
	unpleasant	4	1	4	3	0.0873
	Error	99584	75919	1		

Supplementary table 24. PD-GIS by sociodemographic factors. Parameter estimates in standard deviation (SD) units

		More time, less stressed/tired	Disrupted lifestyle	Health concerns	Positive outlook	Conflict at home	Improved environment	More time for people
Sex	Other	-0.170 **	-0.021	0.012	0.119	0.056	-0.310 ***	-0.098
	Male	-0.082 ***	-0.137 ***	-0.312 ***	-0.196 ***	0.025 **	-0.386 ***	-0.064 ***
	Female	0.000 ***	0.000 ***	0.000 ***	0.000 ***	0.000 ***	0.000 ***	0.000 ***
Handed	Ambidextrous	-0.080 **	-0.111 ***	0.034	0.038	-0.046	-0.021	0.038
	Left	-0.027 *	-0.008	0.012	-0.018	0.009	0.077 ***	-0.009
	Right	0.000 ***	0.000 ***	0.000 ***	0.000 ***	0.000 ***	0.000 ***	0.000 ***
Ethnicity	White	0.000 ***	0.000 ***	0.000 ***	0.000 ***	0.000 ***	0.000 ***	0.000 ***
	Other	0.028	-0.072 *	0.195 ***	0.048	0.001	-0.041	0.068
	Mixed	0.071 **	-0.017	0.033	-0.019	0.016	-0.105 ***	0.076 **
	Hispanic	0.087	-0.163 **	0.311 ***	0.191 **	-0.017	-0.049	0.235 ***
	Asian	0.217 ***	-0.026	0.181 ***	0.273 ***	0.021	-0.263 ***	0.142 ***
	African	0.300 ***	0.002	0.008	0.145 *	-0.122 *	-0.340 ***	-0.029
First language	English	0.000 ***	0.000 ***	0.000 ***	0.000 ***	0.000 ***	0.000 ***	0.000 ***
	Other	0.071 ***	-0.298 ***	0.161 ***	-0.143 ***	-0.034 *	-0.224 ***	-0.099 ***
Country	UK	0.000 ***	0.000 ***	0.000 ***	0.000 ***	0.000 ***	0.000 ***	0.000 ***
	Other	0.016	-0.012	-0.021	-0.023	-0.011	-0.109 ***	-0.001
Education	Primary/elemen	-0.165 ***	-0.013	0.081 **	0.234 ***	0.004	-0.159 ***	0.044
	None	-0.140	-0.272 *	0.274 *	0.364 **	0.250 *	-0.192	0.033
	High school	-0.103 ***	-0.009	0.053 ***	0.147 ***	-0.019 *	-0.149 ***	0.002
	PhD	-0.091 ***	0.008	-0.073 ***	-0.134 ***	-0.022	-0.042	0.054 *
	Degree	0.000 ***	0.000 ***	0.000 ***	0.000 ***	0.000 ***	0.000 ***	0.000 ***
Occupational sta	COVID-19 hea	-0.807 ***	-0.655 ***	0.205 ***	0.198 ***	0.064 *	0.104 **	-0.024
	Disabled/shielde	-0.541 ***	-0.066	0.535 ***	-0.002	0.045	-0.269 ***	0.249 ***
	Frontline health	-0.527 ***	-0.438 ***	0.142 ***	0.186 ***	0.069 **	0.002	0.044
	Office/lab	-0.418 ***	-0.378 ***	0.034	0.101 ***	0.052 *	-0.019	-0.002
	Retail/public fa	-0.351 ***	-0.356 ***	0.134 ***	0.117 ***	0.033	0.021	0.025
	Homemaker	-0.225 ***	-0.167 ***	0.199 ***	0.040	0.082 **	-0.144 ***	0.042
	Retired	-0.171 ***	-0.031	0.147 ***	0.084 ***	0.102 ***	-0.024	-0.267 ***
	Student	-0.031	0.298 ***	-0.081 **	0.242 ***	0.186 ***	-0.135 ***	-0.113 ***
	Job seeker	-0.011	0.123 ***	0.063 *	-0.006	0.107 ***	-0.149 ***	-0.021
	Other	0.000 ***	0.000 ***	0.000 ***	0.000 ***	0.000 ***	0.000 ***	0.000 ***
	Work from hom	0.037 *	-0.102 ***	0.037	0.117 ***	0.066 ***	-0.201 ***	0.205 ***
	Furloughed	0.425 ***	0.267 ***	-0.100 ***	0.087 ***	0.012	-0.009	0.017

Parameter estimates for predictors in the GLM. p<0.05*, p<0..01**, p<0.001. All predictors are binary and can be interpreted as effect sizes in standard deviation units (apart from age, which is reported separately).

Continued overleaf -->

		More time, less stressed/tired	Disrupted lifestyle	Health concerns	Positive outlook	Conflict at home	Improved environment	More time for people
Reduced	No	-0.101 ***	-0.314 ***	-0.139 ***	0.176 ***	-0.056 ***	-0.033 **	-0.056 ***
income	Yes	0.000 ***	0.000 ***	0.000 ***	0.000 ***	0.000 ***	0.000 ***	0.000 ***
Relationship	Seperated	-0.013	0.196 ***	-0.007	0.011	0.065	-0.031	-0.137 ***
status	Single	-0.012	0.077 ***	-0.048 *	-0.002	0.023	-0.184 ***	-0.171 ***
	Married	0.000 ***	0.000 ***	0.000 ***	0.000 ***	0.000 ***	0.000 ***	0.000 ***
	Partner	0.022	0.037 **	0.005	-0.019	0.089 ***	-0.048 ***	-0.084 ***
	Divorced	0.046	0.139 ***	-0.038	0.048	-0.070 **	-0.045	-0.041
	Widowed	0.078 **	0.174 ***	0.058	0.169 ***	-0.051	-0.187 ***	-0.047
Cohabitees	Preschool child	-0.447 ***	-0.071 ***	-0.007	0.072 ***	0.317 ***	-0.046 *	0.584 ***
	School children	-0.114 ***	0.034 **	-0.144 ***	0.059 ***	0.296 ***	0.002	0.415 ***
	Homeschooled	-0.110 ***	-0.016	-0.078 ***	0.068 ***	0.291 ***	0.015	0.454 ***
	Grandparents	-0.076	-0.041	0.200 ***	0.155 **	0.045	-0.107	-0.124 *
	Parents	-0.043 *	0.048 **	0.118 ***	0.010	0.375 ***	-0.029	-0.237 ***
	Housemates	-0.023	0.152 ***	0.065 *	0.031	0.005	-0.009	-0.511 ***
	Partner	-0.018	-0.032 *	-0.063 ***	-0.003	0.063 ***	0.025	0.197 ***
	Adult children	-0.010	0.033 **	-0.094 ***	0.017	0.099 ***	-0.022	0.170 ***
	Inlaws	0.010	0.059	-0.013	0.016	0.174 ***	0.032	-0.119 **
	Alone	0.017	0.160 ***	0.054 **	0.068 ***	-0.105 ***	-0.079 ***	-0.719 ***
	Friends	0.038	0.055	-0.057	0.003	0.006	0.071 *	-0.105 **
Home	River	-0.091	0.021	-0.065	0.096	-0.051	-0.048	0.207 *
	Other	-0.016	-0.106 **	-0.017	0.007	-0.021	-0.176 ***	0.131 **
	Bungalow	0.000	0.021	0.087 ***	0.038	-0.018	-0.097 ***	0.050 **
	Detached	0.000 ***	0.000 ***	0.000 ***	0.000 ***	0.000 ***	0.000 ***	0.000 ***
	Semi	0.004	0.054 ***	0.070 ***	0.013	-0.037 ***	-0.030 *	0.056 ***
	Terrace	0.009	0.062 ***	0.027 *	-0.008	-0.052 ***	-0.021	0.093 ***
	Flat	0.013	0.072 ***	0.032	-0.033	-0.070 ***	-0.016	0.072 ***
Outside	Unpleasant	-0.288 ***	0.302 ***	0.162 ***	-0.147 ***	0.104 ***	-0.285 ***	0.057
	None	-0.097 ***	0.154 ***	0.064 *	-0.050	-0.019	-0.140 ***	0.024
	Overlooked	-0.092 ***	0.134 ***	0.102 ***	-0.054 ***	0.028 *	-0.080 ***	0.075 ***
	Private	-0.033 **	0.011	-0.017	0.005	-0.006	-0.040 **	0.011
	Small garden	-0.020	0.058 ***	-0.030	-0.009	-0.006	0.052 **	0.057 **
	Balcony	0.035	0.020	-0.007	0.013	0.018	-0.073 **	0.021
	Large garden	0.041 *	-0.012	-0.121 ***	0.021	-0.018	0.130 ***	0.074 ***
	Relaxing	0.119 ***	-0.059 ***	-0.072 ***	0.043 ***	-0.071 ***	0.160 ***	0.062 ***

Parameter estimates for predictors in the GLM. p<0.05*, p<0..01**, p<0.001. All predictors are binary and can be interpreted as effect sizes in standard deviation units (apart from age, which is reported separately). Effects sizes highlighted in blue (negative) and green (positive).

Supplementary table 25. **PD-GIS by pre-existing conditions. More time, less stressed and tired. (Parameter estimates in SD units)**

	estimate	N	SE	t	p
Anxiety	-0.028	4141	0	-2	0.0799
Attention deficit hyperactivity disorder	-0.007	485	0	0	0.8850
Bipolar	-0.059	363	0	-1	0.2609
Depression	-0.097	4744	0	-6	<0.0001
Obsessive compulsive disorder	0.064	241	0	1	0.3218
Other psychiatric	-0.011	1157	0	0	0.7062
Learning disability	-0.081	890	0	-2	0.0173
Multiple sclerosis	-0.109	243	0	-2	0.0903
Stroke	0.060	441	0	1	0.2117
Other neurological	-0.024	2093	0	-1	0.2906
Traumatic brain injury	0.049	110	0	1	0.6088
Parkinson's disease	-0.158	122	0	-2	0.0810
OCD & anxiety	-0.119	602	0	-3	0.0053
Depression & anxiety	-0.159	5979	0	-11	<0.0001
Weakened immune system	-0.045	2082	0	-2	0.0443
Kidney disease	-0.025	545	0	-1	0.5638
Diabetes	-0.034	2858	0	-2	0.0749
Heart disease	-0.027	2161	0	-1	0.2227
High blood pressure	0.014	744	0	0	0.7138
Irregular heart beat	0.042	181	0	1	0.5761
Liver disease	0.034	364	0	1	0.5223
Lung condition	-0.032	8226	0	-3	0.0064
Spleen/ sickle cell disease	-0.015	153	0	0	0.8492

Supplementary table 26. **PD-GIS by pre-existing conditions. Disrupted lifestyle. (Parameter estimates in SD units)**

	estimate	N	SE	t	
Anxiety	0.092	4141	0	6	<0.0001
Attention deficit hyperactivity disorder	0.123	485	0	3	0.0074
Bipolar	0.109	363	0	2	0.0392
Depression	0.259	4744	0	17	<0.0001
Obsessive compulsive disorder	-0.046	241	0	-1	0.4718
Other psychiatric	0.025	1157	0	1	0.4128
Learning disability	-0.047	890	0	-1	0.1601
Multiple sclerosis	-0.038	243	0	-1	0.5555
Stroke	0.019	441	0	0	0.6942
Other neurological	-0.044	2093	0	-2	0.0459
Traumatic brain injury	-0.050	110	0	-1	0.6006
Parkinson's disease	0.016	122	0	0	0.8621
OCD & anxiety	-0.037	602	0	-1	0.3883
Depression & anxiety	0.301	5979	0	22	<0.0001
Weakened immune system	0.040	2082	0	2	0.0741
Kidney disease	0.031	545	0	1	0.4758
Diabetes	0.046	2858	0	2	0.0153
Heart disease	0.074	2161	0	3	0.0007
High blood pressure	-0.004	744	0	0	0.9091
Irregular heart beat	-0.032	181	0	0	0.6695
Liver disease	0.030	364	0	1	0.5709
Lung condition	0.024	8226	0	2	0.0371
Spleen/ sickle cell	0.060	153	0	1	0.4540

Supplementary table 27. **PD-GIS by pre-existing conditions. Increased health concerns. (Parameter estimates in SD units)**

	estimate	N	SE	t	
Anxiety	0.301	4141	0	19	<0.0001
Attention deficit hyperactivity disorder	-0.013	485	0	0	0.7689
Bipolar	0.010	363	0	0	0.8503
Depression	0.056	4744	0	4	0.0003
Obsessive compulsive disorder	0.226	241	0	4	0.0004
Other psychiatric	0.006	1157	0	0	0.8389
Learning disability	0.040	890	0	1	0.2384
Multiple sclerosis	0.009	243	0	0	0.8822
Stroke	-0.019	441	0	0	0.6851
Other neurological	0.040	2093	0	2	0.0727
Traumatic brain injury	0.030	110	0	0	0.7493
Parkinson's disease	0.005	122	0	0	0.9519
OCD & anxiety	0.486	602	0	12	<0.0001
Depression & anxiety	0.278	5979	0	20	<0.0001
Weakened immune system	0.265	2082	0	12	<0.0001
Kidney disease	0.111	545	0	3	0.0095
Diabetes	0.212	2858	0	11	<0.0001
Heart disease	0.115	2161	0	5	<0.0001
High blood pressure	0.051	744	0	1	0.1646
Irregular heart beat	0.173	181	0	2	0.0200
Liver disease	0.056	364	0	1	0.2822
Lung condition	0.220	8226	0	19	<0.0001
Spleen/ sickle cell	0.104	153	0	1	0.1960

Supplementary table 28. **PD-GIS by pre-existing conditions. Positive outlook. (Parameter estimates in SD units)**

	estimate	N	SE	t	
Anxiety	0.034	4141	0	2	0.0341
Attention deficit hyperactivity disorder	0.086	485	0	2	0.0616
Bipolar	0.047	363	0	1	0.3791
Depression	-0.040	4744	0	-3	0.0109
Obsessive compulsive disorder	0.006	241	0	0	0.9278
Other psychiatric	-0.038	1157	0	-1	0.2134
Learning disability	0.058	890	0	2	0.0879
Multiple sclerosis	-0.075	243	0	-1	0.2454
Stroke	0.100	441	0	2	0.0365
Other neurological	-0.006	2093	0	0	0.7842
Traumatic brain injury	0.095	110	0	1	0.3180
Parkinson's disease	0.009	122	0	0	0.9235
OCD & anxiety	0.044	602	0	1	0.2979
Depression & anxiety	-0.029	5979	0	-2	0.0342
Weakened immune system	0.062	2082	0	3	0.0056
Kidney disease	0.021	545	0	0	0.6202
Diabetes	0.089	2858	0	5	<0.0001
Heart disease	0.011	2161	0	1	0.6064
High blood pressure	-0.137	744	0	-4	0.0002
Irregular heart beat	-0.041	181	0	-1	0.5895
Liver disease	-0.064	364	0	-1	0.2274
Lung condition	0.007	8226	0	1	0.5695
Spleen/ sickle cell	0.067	153	0	1	0.4087

Supplementary table 29. **PD-GIS by pre-existing conditions. Conflict at home. (Parameter estimates in SD units)**

	estimate	N	SE	t	
Anxiety	-0.009	4141	0	-1	0.5950
Attention deficit hyperactivity disorder	0.176	485	0	4	0.0001
Bipolar	-0.097	363	0	-2	0.0663
Depression	0.081	4744	0	5	<0.0001
Obsessive compulsive disorder	0.110	241	0	2	0.0885
Other psychiatric	0.094	1157	0	3	0.0020
Learning disability	0.046	890	0	1	0.1788
Multiple sclerosis	0.061	243	0	1	0.3431
Stroke	0.042	441	0	1	0.3827
Other neurological	0.072	2093	0	3	0.0013
Traumatic brain injury	0.071	110	0	1	0.4591
Parkinson's disease	-0.002	122	0	0	0.9822
OCD & anxiety	0.016	602	0	0	0.7010
Depression & anxiety	0.066	5979	0	5	<0.0001
Weakened immune system	-0.039	2082	0	-2	0.0817
Kidney disease	-0.026	545	0	-1	0.5555
Diabetes	-0.014	2858	0	-1	0.4803
Heart disease	-0.012	2161	0	-1	0.5876
High blood pressure	0.044	744	0	1	0.2397
Irregular heart beat	0.039	181	0	1	0.6027
Liver disease	0.025	364	0	0	0.6295
Lung condition	-0.030	8226	0	-3	0.0100
Spleen/ sickle cell	0.023	153	0	0	0.7775

Supplementary table 30. **PD-GIS by pre-existing conditions. Improved environment. (Parameter estimates in SD units)**

	estimate	N	SE	t	
Anxiety	0.036	4141	0	2	0.0243
Attention deficit hyperactivity disorder	-0.132	485	0	-3	0.0042
Bipolar	-0.220	363	0	-4	<0.0001
Depression	0.013	4744	0	1	0.4040
Obsessive compulsive disorder	-0.015	241	0	0	0.8137
Other psychiatric	-0.006	1157	0	0	0.8546
Learning disability	-0.070	890	0	-2	0.0384
Multiple sclerosis	-0.016	243	0	0	0.8013
Stroke	-0.069	441	0	-1	0.1508
Other neurological	-0.103	2093	0	-5	<0.0001
Traumatic brain injury	0.035	110	0	0	0.7139
Parkinson's disease	-0.247	122	0	-3	0.0064
OCD & anxiety	-0.076	602	0	-2	0.0749
Depression & anxiety	0.017	5979	0	1	0.2095
Weakened immune system	-0.032	2082	0	-1	0.1530
Kidney disease	-0.143	545	0	-3	0.0010
Diabetes	-0.168	2858	0	-9	<0.0001
Heart disease	-0.060	2161	0	-3	0.0062
High blood pressure	-0.004	744	0	0	0.9216
Irregular heart beat	-0.136	181	0	-2	0.0709
Liver disease	-0.110	364	0	-2	0.0369
Lung condition	-0.015	8226	0	-1	0.2047
Spleen/ sickle cell	-0.050	153	0	-1	0.5374

Supplementary table 31. PD-GIS by pre-existing conditions. More time for people at home. (Parameter estimates in SD units)

	estimate	N	SE	t	
Anxiety	0.090	4141	0	6	
Attention deficit hyperactivity disorder	0.063	485	0	1	0.1683
Bipolar	0.069	363	0	1	0.1894
Depression	0.026	4744	0	2	0.0904
Obsessive compulsive disorder	0.008	241	0	0	0.9060
Other psychiatric	-0.044	1157	0	-1	0.1521
Learning disability	0.040	890	0	1	0.2333
Multiple sclerosis	-0.071	243	0	-1	0.2685
Stroke	0.010	441	0	0	0.8301
Other neurological	0.001	2093	0	0	0.9510
Traumatic brain injury	0.065	110	0	1	0.4944
Parkinson's disease	-0.013	122	0	0	0.8896
OCD & anxiety	0.033	602	0	1	0.4421
Depression & anxiety	0.112	5979	0	8	<0.0001
Weakened immune system	0.092	2082	0	4	<0.0001
Kidney disease	0.065	545	0	1	0.1358
Diabetes	0.117	2858	0	6	<0.0001
Heart disease	0.028	2161	0	1	0.2048
High blood pressure	0.012	744	0	0	0.7494
Irregular heart beat	0.177	181	0	2	0.0187
Liver disease	0.049	364	0	1	0.3565
Lung condition	0.067	8226	0	6	<0.0001
Spleen/ sickle cell	0.125	153	0	2	0.1222

Principal Component Analyses of questionnaire scales.
Note – here we conform to the Kaiser convention of including components with eigenvalues > 1 in all analyses.

Big 5 optimised (reduced sub-set of Big5 personality measures)

Supplementary table 32. **Bivariate correlations for the Big 5 personality**

	Talkative	Thorough job	Original/ new ideas	reserved	Relaxed, handles stress well	Forgiving	Disorgansied	Worries a lot	tends to be queit	Stable/ not easily upset	inventive	Perseveres till the task is finished	Values artistic/aesthetci experiences	Kind/considerate to almost everyone	Likes to reflect/play with ideas	Few artistic instincts	Cooperative	Sophisticated in art/music/literature
**Q1**		0.09	0.21	-0.53	0.03	0.08	0.05	0.01	-0.42	-0.02	0.11	0.03	0.11	0.14	0.08	-0.03	0.19	0.11
**Q2**	0.09		0.16	0.04	0.09	0.05	-0.32	0.01	-0.13	0.12	0.11	0.53	0.09	0.20	0.11	0.00	0.17	0.04
**Q3**	0.21	0.16		-0.12	0.17	0.09	0.06	-0.08	-0.12	0.12	0.69	0.16	0.29	0.11	0.43	-0.15	0.06	0.25
**Q4**	-0.53	0.04	-0.12		-0.05	0.00	0.03	0.18	0.49	-0.01	-0.07	0.04	-0.02	0.00	0.03	0.06	-0.07	-0.03
**Q5**	0.03	0.09	0.17	-0.05		0.24	-0.05	-0.54	-0.04	0.59	0.18	0.12	0.00	0.12	0.10	0.04	0.14	0.02
**Q6**	0.08	0.05	0.09	0.00	0.24		0.09	-0.06	0.00	0.21	0.10	0.08	0.13	0.43	0.17	-0.02	0.32	0.08
**Q7**	0.05	-0.32	0.06	0.03	-0.05	0.09		0.12	0.19	-0.10	0.08	-0.29	0.05	-0.01	0.10	0.00	-0.04	0.05
**Q8**	0.01	0.01	-0.08	0.18	-0.54	-0.06	0.12		0.14	-0.48	-0.10	-0.02	0.09	0.07	0.03	0.00	0.00	0.05
**Q9**	-0.42	-0.13	-0.12	0.49	-0.04	0.00	0.19	0.14		-0.03	-0.07	-0.12	-0.01	-0.02	0.01	0.06	-0.10	-0.01
**Q10**	-0.02	0.12	0.12	-0.01	0.59	0.21	-0.10	-0.48	-0.03		0.17	0.17	-0.01	0.11	0.08	0.04	0.15	0.00
**Q11**	0.11	0.11	0.69	-0.07	0.18	0.10	0.08	-0.10	-0.07	0.17		0.18	0.30	0.09	0.44	-0.16	0.04	0.25
**Q12**	0.03	0.53	0.16	0.04	0.12	0.08	-0.29	-0.02	-0.12	0.17	0.18		0.12	0.18	0.15	0.00	0.17	0.04
**Q13**	0.11	0.09	0.29	-0.02	0.00	0.13	0.05	0.09	-0.01	-0.01	0.30	0.12		0.22	0.37	-0.41	0.14	0.56
**Q14**	0.14	0.20	0.11	0.00	0.12	0.43	-0.01	0.07	-0.02	0.11	0.09	0.18	0.22		0.23	-0.04	0.41	0.13
**Q15**	0.08	0.11	0.43	0.03	0.10	0.17	0.10	0.03	0.01	0.08	0.44	0.15	0.37	0.23		-0.15	0.16	0.29
**Q16**	-0.03	0.00	-0.15	0.06	0.04	-0.02	0.00	0.00	0.06	0.04	-0.16	0.00	-0.41	-0.04	-0.15		0.00	-0.37
**Q17**	0.19	0.17	0.06	-0.07	0.14	0.32	-0.04	0.00	-0.10	0.15	0.04	0.17	0.14	0.41	0.16	0.00		0.09
**Q18**	0.11	0.04	0.25	-0.03	0.02	0.08	0.05	0.05	-0.01	0.00	0.25	0.04	0.56	0.13	0.29	-0.37	0.09	

Supplementary figure 12. **Principle component analysis with varimax rotation for the Big 5 personality**

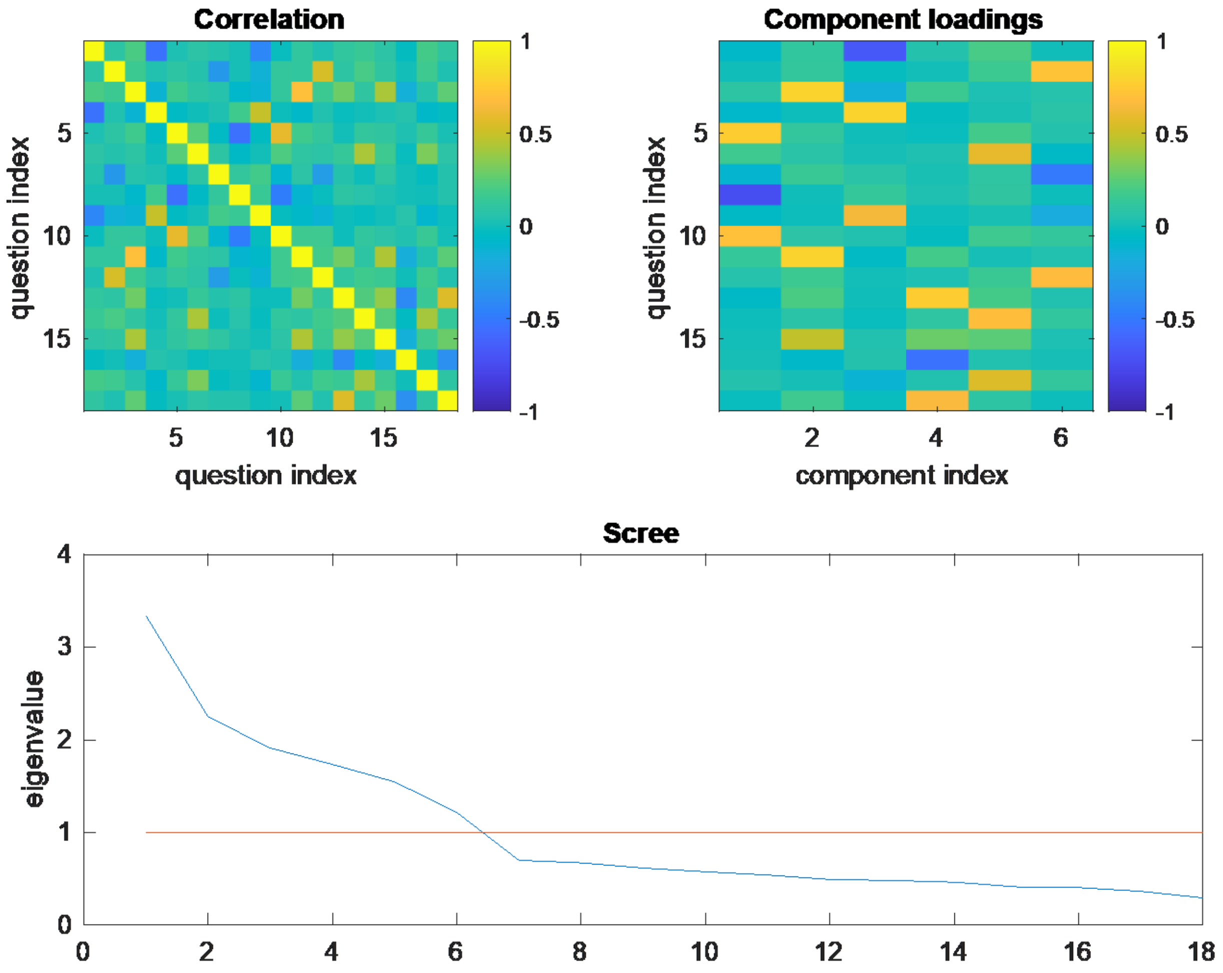

Top left – correlation matrix for the Big 5 optimised personality scale. Top right, task-component loadings after varimax rotation. Bottom – scree plot of eigenvalues. Source data are provided as a Source Data file.

Supplementary table 33. **Varimax rotated component loadings table for the Big 5 personality.**

Secure	Open	Introverted	Artistic	Compassionate	Conscientious	
-0.06	0.12	**-0.67**	0.05	0.20	0.00	Talkative
0.00	0.12	-0.03	0.01	0.16	**0.72**	Thorough job
0.09	**0.80**	-0.14	0.16	0.03	0.06	Original/ new ideas
-0.09	-0.01	**0.80**	-0.03	0.02	0.08	reserved
**0.76**	0.12	0.00	-0.02	0.19	0.05	Relaxed, handles stress well
0.17	0.07	0.01	0.03	**0.59**	-0.06	Forgiving
-0.11	0.14	0.07	0.02	0.10	**-0.49**	Disorganised
**-0.73**	0.00	0.13	0.03	0.10	-0.01	Worries a lot
-0.06	-0.01	**0.63**	-0.01	0.02	-0.19	tends to be quiet
**0.70**	0.09	0.04	-0.03	0.17	0.12	Stable/ not easily upset
0.13	**0.80**	-0.05	0.18	0.01	0.04	inventive
0.05	0.16	0.01	0.03	0.15	**0.68**	Perseveres till the task is finished
-0.06	0.21	-0.01	**0.76**	0.19	0.04	Values artistic/aesthetic experiences
-0.01	0.07	-0.02	0.10	**0.68**	0.11	Kind/considerate to almost everyone
0.00	**0.48**	0.04	0.28	0.25	0.03	Likes to reflect/play with ideas
0.01	-0.07	0.05	**-0.54**	0.04	0.01	Few artistic instincts
0.05	0.01	-0.12	0.05	**0.56**	0.12	Cooperative
-0.02	0.17	-0.02	**0.66**	0.09	-0.01	Sophisticated in art/music/literature

Supplementary table 34. **Bivariate correlations for the technology use scale**

	Smart phone	Computer	Tablet	Games console	Email	Social media	News	Games	Gamble	Work	Learn/study	Shop	Stream	Search	Hrs per day
**Q1**		0.25	0.04	0.16	0.31	0.41	0.16	0.09	0.09	0.26	0.17	0.20	0.32	0.34	0.31
**Q2**	0.25		0.02	0.09	0.38	0.08	0.18	0.12	0.04	0.53	0.24	0.14	0.20	0.29	0.32
**Q3**	0.04	0.02		0.02	0.16	-0.01	0.10	0.06	0.01	0.05	0.00	0.16	-0.05	0.06	-0.03
**Q4**	0.16	0.09	0.02		-0.01	0.13	-0.02	0.56	0.21	0.07	0.14	0.05	0.30	0.10	0.22
**Q5**	0.31	0.38	0.16	-0.01		0.14	0.29	0.00	0.05	0.40	0.09	0.25	0.10	0.31	0.20
**Q6**	0.41	0.08	-0.01	0.13	0.14		0.05	0.08	0.07	0.11	0.16	0.14	0.27	0.22	0.31
**Q7**	0.16	0.18	0.10	-0.02	0.29	0.05		0.01	0.05	0.18	0.08	0.13	0.08	0.26	0.09
**Q8**	0.09	0.12	0.06	0.56	0.00	0.08	0.01		0.16	0.03	0.09	0.04	0.20	0.10	0.21
**Q9**	0.09	0.04	0.01	0.21	0.05	0.07	0.05	0.16		0.06	0.02	0.08	0.11	0.04	0.09
**Q10**	0.26	0.53	0.05	0.07	0.40	0.11	0.18	0.03	0.06		0.32	0.19	0.23	0.33	0.29
**Q11**	0.17	0.24	0.00	0.14	0.09	0.16	0.08	0.09	0.02	0.32		0.13	0.30	0.28	0.26
**Q12**	0.20	0.14	0.16	0.05	0.25	0.14	0.13	0.04	0.08	0.19	0.13		0.20	0.23	0.15
**Q13**	0.32	0.20	-0.05	0.30	0.10	0.27	0.08	0.20	0.11	0.23	0.30	0.20		0.35	0.36
**Q14**	0.34	0.29	0.06	0.10	0.31	0.22	0.26	0.10	0.04	0.33	0.28	0.23	0.35		0.33
**Q15**	0.31	0.32	-0.03	0.22	0.20	0.31	0.09	0.21	0.09	0.29	0.26	0.15	0.36	0.33	

Supplementary figure 13. **Principle component analysis with varimax rotation for technology use**

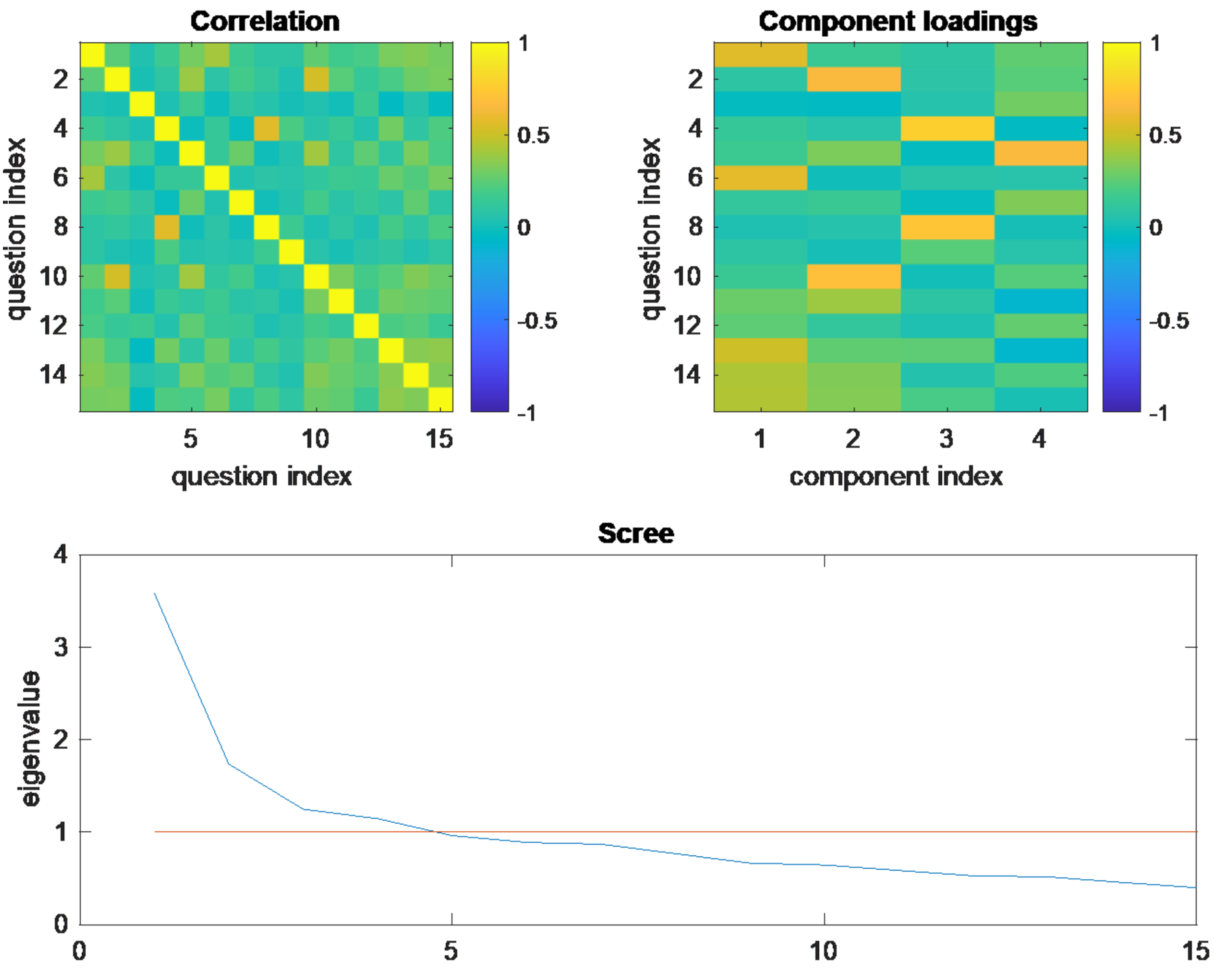

Top left – correlation matrix for items of the technology use scale. Top right, task-component loadings after varimax rotation. Bottom – scree plot of eigenvalues. Source data are provided as a Source Data file.

Supplementary table 35. **Varimax rotated component loadings table for the technology use scale**

Phone, social, entertainment	Computer, Work, study	gaming	Email, news and info	
0.567	0.154	0.090	0.258	Smart phone
0.092	0.651	0.080	0.241	Computer
-0.035	-0.041	0.062	0.301	Tablet
0.140	0.065	0.774	-0.046	Games console
0.164	0.325	-0.039	0.652	Email
0.586	0.000	0.073	0.063	Social media
0.118	0.153	-0.024	0.335	News
0.036	0.059	0.719	0.012	Games
0.086	0.015	0.235	0.067	Gamble
0.155	0.691	0.002	0.233	Work
0.286	0.394	0.089	-0.094	Learn/study
0.254	0.119	0.037	0.266	Shop
0.515	0.262	0.252	-0.086	Stream
0.434	0.332	0.054	0.216	Search
0.442	0.338	0.200	0.023	Hrs per day

Supplementary table 36. **Bivariate correlations for the technology stress scale**

	Q1	Q2	Q3	Q4
Email		0.19	0.15	0.03
Social media	0.19		0.17	0.12
News	0.15	0.17		-0.02
Computer games	0.03	0.12	-0.02	

Supplementary figure 14. **Principle component analysis with varimax rotation for technology stress**

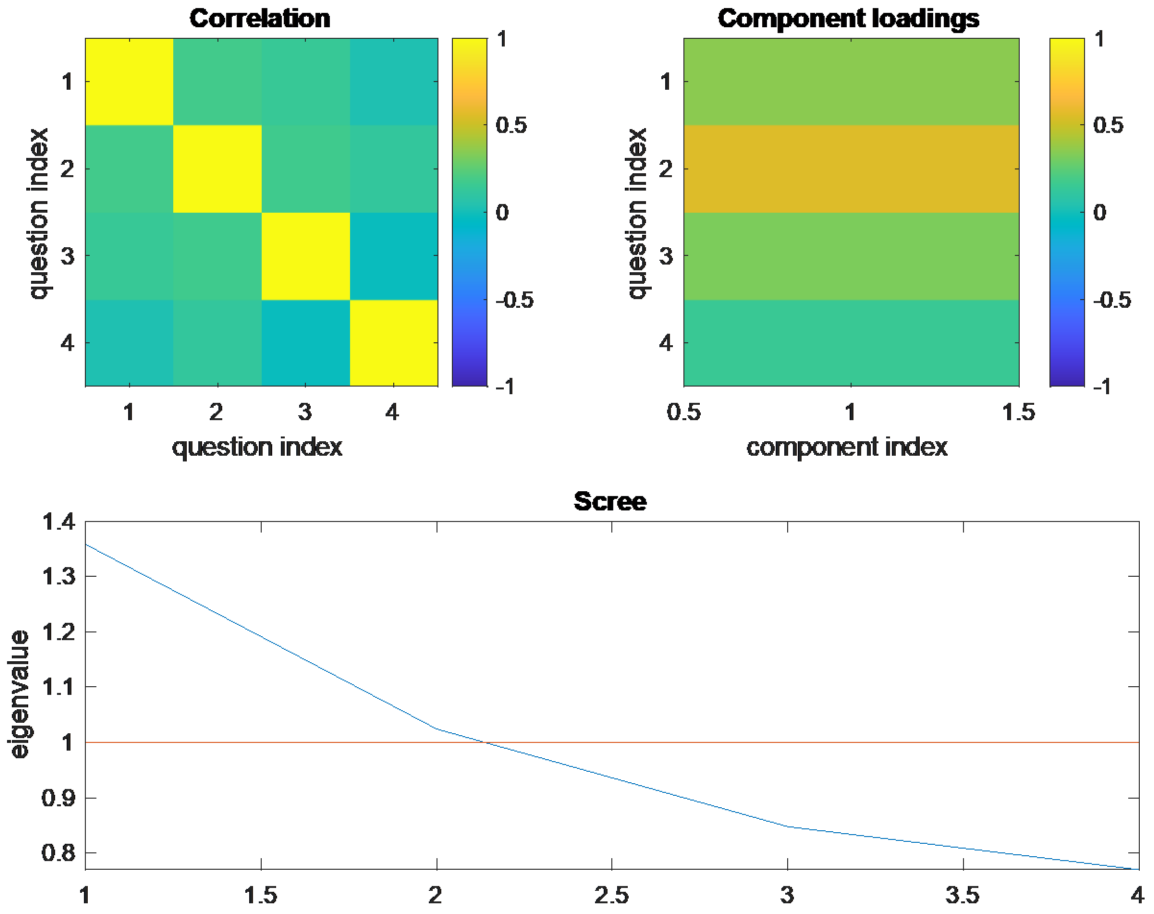

Top left – correlation matrix for the technology stress scale items. Top right, task-component loadings after varimax rotation. Bottom – scree plot of eigenvalues. Source data are provided as a Source Data file.

Supplementary table 37. **Varimax rotated component loadings table for technology stress**

Loadings	Q - stress from
0.35	Email
0.56	Social media
0.32	News
0.15	Computer games

Supplementary table 38. **Bivariate correlations for the technology addiction scale**

	Q1	Q2	Q3	Q4	Q5	Q6	Q7
**Q1**		0.37	0.34	0.15	0.43	0.29	0.28
**Q2**	0.37		0.58	0.24	0.41	0.41	0.36
**Q3**	0.34	0.58		0.23	0.45	0.42	0.36
**Q4**	0.15	0.24	0.23		0.21	0.30	0.25
**Q5**	0.43	0.41	0.45	0.21		0.50	0.44
**Q6**	0.29	0.41	0.42	0.30	0.50		0.68
**Q7**	0.28	0.36	0.36	0.25	0.44	0.68	

Supplementary table 15. Principle component analysis with varimax rotation for technology addiction

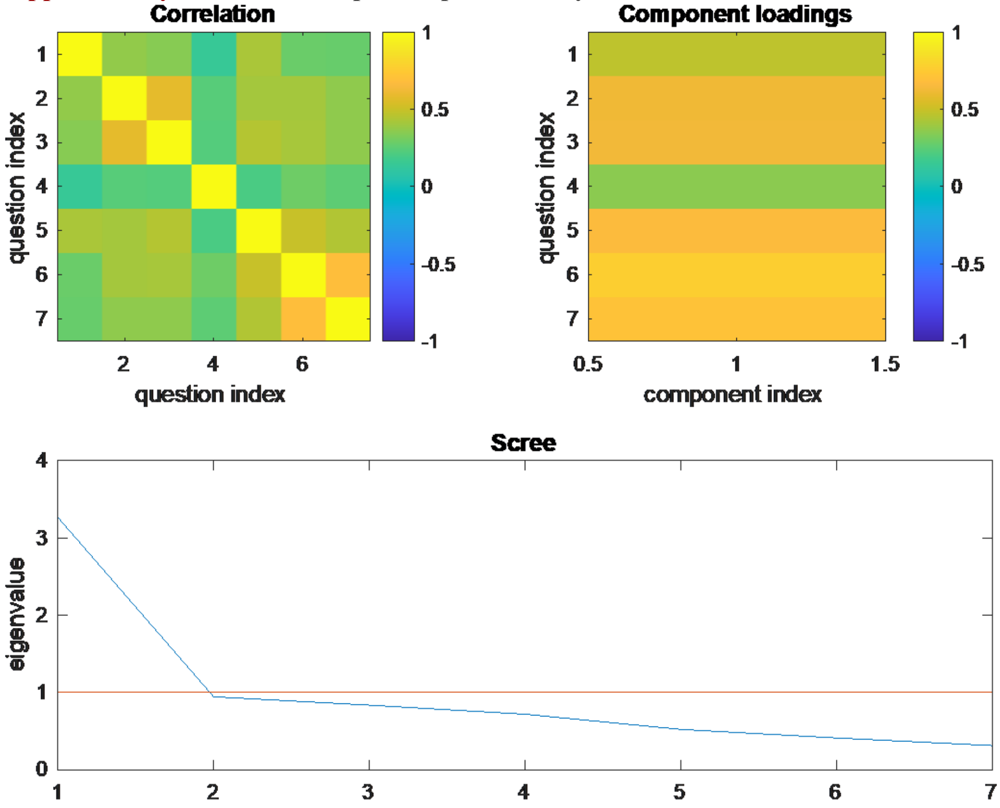

Top left – correlation matrix for the technology addiction scale items. Top right, task-component loadings after varimax rotation. Bottom – scree plot of eigenvalues. Source data are provided as a Source Data file.

Supplementary table 39. Varimax rotated component loadings table for technology addiction

0.48	Q1 Check email/social media in bed
0.62	Q2 Internet activities to sooth self / block out disturbing thoughts
0.63	Q3 Time online to battle loneliness
0.36	Q4 Negative financial consequences due to online pursuits
0.67	Q5 Check email/social media before something important
0.77	Q6 Try to stop online activity but feel compelled
0.71	Q7 Try to cut down time online but fail

Supplementary table 40. Bivariate correlations for the compulsivity scale (CHIT)

	Q1	Q2	Q3	Q4	Q5	Q6	Q7	Q8	Q9	Q10	Q11	Q12	Q13	Q14
**Q1**		0.45	0.40	0.10	0.16	0.02	0.07	-0.04	-0.11	0.27	0.29	0.37	0.12	0.16
**Q2**	0.45		0.52	0.22	0.28	0.09	0.17	0.02	-0.02	0.38	0.42	0.46	0.23	0.25
**Q3**	0.40	0.52		0.28	0.20	0.12	0.16	0.05	-0.04	0.48	0.45	0.42	0.21	0.34
**Q4**	0.10	0.22	0.28		0.34	0.31	0.24	0.21	0.23	0.37	0.20	0.31	0.32	0.28
**Q5**	0.16	0.28	0.20	0.34		0.25	0.22	0.06	0.10	0.25	0.15	0.27	0.34	0.17
**Q6**	0.02	0.09	0.12	0.31	0.25		0.28	0.34	0.39	0.21	0.14	0.16	0.17	0.24
**Q7**	0.07	0.17	0.16	0.24	0.22	0.28		0.25	0.20	0.22	0.22	0.18	0.19	0.23
**Q8**	-0.04	0.02	0.05	0.21	0.06	0.34	0.25		0.48	0.17	0.06	0.09	0.09	0.17
**Q9**	-0.11	-0.02	-0.04	0.23	0.10	0.39	0.20	0.48		0.19	0.02	0.08	0.13	0.15
**Q10**	0.27	0.38	0.48	0.37	0.25	0.21	0.22	0.17	0.19		0.39	0.43	0.33	0.36
**Q11**	0.29	0.42	0.45	0.20	0.15	0.14	0.22	0.06	0.02	0.39		0.46	0.18	0.34
**Q12**	0.37	0.46	0.42	0.31	0.27	0.16	0.18	0.09	0.08	0.43	0.46		0.30	0.31
**Q13**	0.12	0.23	0.21	0.32	0.34	0.17	0.19	0.09	0.13	0.33	0.18	0.30		0.34
**Q14**	0.16	0.25	0.34	0.28	0.17	0.24	0.23	0.17	0.15	0.36	0.34	0.31	0.34	

Supplementary figure 16. **Principle component analysis with varimax rotation for the compulsivity scale (CHIT)**

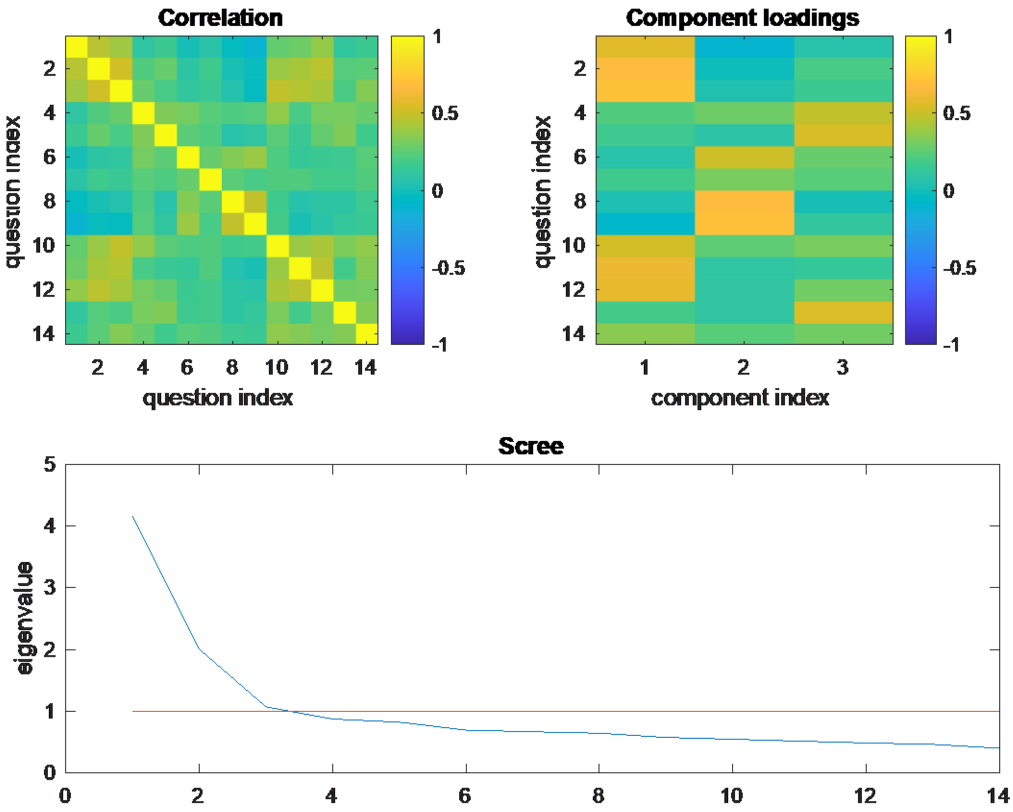

Top left – correlation matrix for the CHIT compulsivity scale items. Top right, task-component loadings after varimax rotation. Bottom – scree plot of eigenvalues. Source data are provided as a Source Data file.

Supplementary table 41. **Varimax rotated component loadings table for compulsivity scale (CHIT)**

Perfectionism	Reward drive	Cognitive rigidity	
**0.57**	-0.11	0.06	Leaving tasks unfinished
**0.67**	-0.01	0.20	Doing things just right
**0.71**	0.04	0.16	Completion to high standard
0.22	0.29	**0.48**	Repetitive thoughts
0.17	0.08	**0.54**	Habits
0.06	**0.51**	0.28	Addictive personality
0.17	**0.32**	0.24	Stubborn/rigid
0.03	**0.67**	0.01	Acting on urges
-0.08	**0.70**	0.11	Immediate reward
**0.53**	0.25	0.32	Obsession with perfection
**0.61**	0.10	0.13	Higher standards than others
**0.59**	0.11	0.30	Soothed by completing tasks
0.19	0.10	**0.55**	Avoid uncontrolled situations
**0.36**	0.24	0.30	Need to be the best at things

Figure - data key

Data are available for third party analysis via the UK Data Service. Downloadable as 'COVID-19 impact dataset: Great British Intelligence Test, 2020'. Data and analyses from main text display items are available as follows.

Figure 1. Study schematic
- no data

Figure 2. Visual comparison of mental health and sleep measures during the Pre-UK Pandemic and Mid-UK Lockdown epochs
- Raw data - 01_raw_pre_and_mid_stage_data.mat
- Analyses - a01_MHcounts.mat

Figure 3. Modulation of differences in national mental health scores by population variables
- Raw data - 01_raw_pre_and_mid_stage_data.mat
- Analyses - a02_preVmid.mat & a03_preVmid_bydemographics.mat
- Estimates - Supplement 4

Figure 4. Individual item responses & principal component analysis for the PD-GIS at peak UK lockdown
- Raw data - 02_raw_mid_data_with_PDGIS.mat
- Analyses - a05_PDGIS_analysis.mat
- Estimates - Supplement 6

Figure 5. Interrelationships between the PD-GIS sub-scales and mental health assessment
- Raw data - 02_raw_mid_data_with_PDGIS.mat

Figure 6. Self-perceived pandemic impact by age
- Raw data - 02_raw_mid_data_with_PDGIS.mat
- Analyses - a05_PDGIS_analysis.mat
- Estimates - Supplement 8

Figure 7. Self-perceived pandemic impact by occupational status and cohabitees
- Raw data - 02_raw_mid_data_with_PDGIS.mat
- Analyses - a05_PDGIS_analysis.mat
- Estimates - Supplement 8

Figure 8. Self-perceived pandemic impact by outside space at home
- Raw data - 02_raw_mid_data_with_PDGIS.mat
- Analyses - a05_PDGIS_analysis.mat
- Estimates - Supplement 8

Figure 9. Self-perceived impact of the COVID-19 pandemic by pre-existing conditions
- Raw data - 02_raw_mid_data_with_PDGIS.mat
- Analyses - a05_PDGIS_analysis.mat
- Estimates - Supplement 8

Figure 10. Correlation of Trait and Technology with PD-GIS component scores
- Raw data - 02_raw_mid_data_with_PDGIS.mat
- Analyses - a05_PDGIS_analysis.mat

Supplementary references

1 Kroenke, K., Spitzer, R. L. & Williams, J. B. The Patient Health Questionnaire-2: validity of a two-item depression screener. *Med Care* **41**, 1284-1292, doi:10.1097/01.MLR.0000093487.78664.3C (2003).

2 Spitzer, R. L., Kroenke, K., Williams, J. B. & Lowe, B. A brief measure for assessing generalized anxiety disorder: the GAD-7. *Arch Intern Med* **166**, 1092-1097, doi:10.1001/archinte.166.10.1092 (2006).

3 John O.P, Naumann L.P & C.J., S. in *Handbook of personality: Theory and research* (ed R. W. Robins O. P. John, & L. A. Pervin) (Guilford Press, 2008).

4 Chamberlain, S. R. & Grant, J. E. Initial validation of a transdiagnostic compulsivity questionnaire: the Cambridge-Chicago Compulsivity Trait Scale. *CNS Spectr* **23**, 340-346, doi:10.1017/S1092852918000810 (2018).

5 Albertella, L. *et al.* Compulsivity is measurable across distinct psychiatric symptom domains and is associated with familial risk and reward-related attentional capture. *CNS Spectr*, 1-8, doi:10.1017/S1092852919001330 (2019).

6 Horn, J. L. A rationale and test for the number of factors in factor analysis. *Psychometrika* (1965).
