# Supplementary material for: Associations between dimensions of behaviour, personality traits, and mental-health during the COVID-19 pandemic in the United Kingdom
Source: Nat Commun. 2021 Jul 16;12:4111. doi: 10.1038/s41467-021-24365-5 (PMC8285408; doi:10.1038/s41467-021-24365-5)
Supplement: Supplementary file 3 [file 41467_2021_24365_MOESM3_ESM.pdf]

nature research

Corresponding author(s): Adam Hampshire

Last updated by author(s): 14/05/2021

Reporting Summary

Nature Research wishes to improve the reproducibility of the work that we publish. This form provides structure for consistency and transparency in reporting. For further information on Nature Research policies, see our Editorial Policies and the Editorial Policy Checklist.

Statistics

For all statistical analyses, confirm that the following items are present in the figure legend, table legend, main text, or Methods section.

n/a	Confirmed	
☐	☒	The exact sample size (*n*) for each experimental group/condition, given as a discrete number and unit of measurement
☐	☒	A statement on whether measurements were taken from distinct samples or whether the same sample was measured repeatedly
☐	☒	The statistical test(s) used AND whether they are one- or two-sided<br>*Only common tests should be described solely by name; describe more complex techniques in the Methods section.*
☐	☒	A description of all covariates tested
☐	☒	A description of any assumptions or corrections, such as tests of normality and adjustment for multiple comparisons
☐	☒	A full description of the statistical parameters including central tendency (e.g. means) or other basic estimates (e.g. regression coefficient) AND variation (e.g. standard deviation) or associated estimates of uncertainty (e.g. confidence intervals)
☐	☒	For null hypothesis testing, the test statistic (e.g. *F*, *t*, *r*) with confidence intervals, effect sizes, degrees of freedom and *P* value noted<br>*Give P values as exact values whenever suitable.*
☒	☐	For Bayesian analysis, information on the choice of priors and Markov chain Monte Carlo settings
☐	☒	For hierarchical and complex designs, identification of the appropriate level for tests and full reporting of outcomes
☐	☒	Estimates of effect sizes (e.g. Cohen's *d*, Pearson's *r*), indicating how they were calculated

Our web collection on statistics for biologists contains articles on many of the points above.

Software and code

Policy information about availability of computer code

Data collection	Collection of data is detailed in the main text. Data were collected via our custom server system, which produces study-specific cognitive testing and survey websites (https://gbws.cognitron.co.uk) on the Amazon EC2. Questionnaires and tests were programmed in JavaScript and HTML5. They were deliverable via personal computers, tablets and smartphones.
Data analysis	All analyses were conducted in MATLAB.

For manuscripts utilizing custom algorithms or software that are central to the research but not yet described in published literature, software must be made available to editors and reviewers. We strongly encourage code deposition in a community repository (e.g. GitHub). See the Nature Research guidelines for submitting code & software for further information.

Data

Policy information about availability of data

All manuscripts must include a data availability statement. This statement should provide the following information, where applicable:

- Accession codes, unique identifiers, or web links for publicly available datasets
- A list of figures that have associated raw data
- A description of any restrictions on data availability

All statistical models alongside MATLAB commands for calling them and the analysed data are available via the UK Data Service. The link to the data will be provided on acceptance for publication.

Field-specific reporting

Please select the one below that is the best fit for your research. If you are not sure, read the appropriate sections before making your selection.

☐ Life sciences ☒ Behavioural & social sciences ☐ Ecological, evolutionary & environmental sciences

For a reference copy of the document with all sections, see nature.com/documents/nr-reporting-summary-flat.pdf

Behavioural & social sciences study design

All studies must disclose on these points even when the disclosure is negative.

Study description	This study reports analyses of comprehensive survey data comprising quantitative and categorical measures from self report questionnaires.
Research sample	The dataset includes measures from a very large sample of the general public, predominantly within the UK. It is a very inclusive and representative cross section of the population. This broad sample was selected to enable analysis of mental health and pandemic impact in relation to population variables.
Sampling strategy	Starting from December 26th, 2019, participants were recruited to the study website, where they completed cognitive tests and a detailed questionnaire. The sampling approach was large-scale and non-probabilistic. Specifically, to maximise visibility and inclusiveness articles describing the study were placed on the BBC2 Horizon, BBC homepage, BBC News homepage and circulated on mobile news meta-apps from January 1st, 202052. To maximise representativeness of the sample there were no inclusion/exclusion criteria. Analyses here exclude data from participants under 16 years old, as they completed a briefer questionnaire, and those who responded to the questionnaire unfeasibly fast (<4 minutes). Data were collected from individuals at a particular time point, rather than data being collected serially from the same individuals. This broad sample was selected to enable analysis of mental health and pandemic impact in relation to population variables.
Data collection	All data were collected via a custom website and recorded automatically into a database. People responded using mouse or touch screen interfaces dependent on whether they were using desktop/laptop or mobile devices.
Timing	Data were collected continuously throughout 2020
Data exclusions	To maximise representativeness of the sample there were no inclusion/exclusion criteria. Analyses here exclude data from participants under 16 years old, as they completed a briefer questionnaire, and those who responded to the questionnaire unfeasibly fast (<4 minutes).
Non-participation	Not applicable, we only know who it is that participated in response to advertisement.
Randomization	Participants were not allocated into groups, instead, mental health and self-perceived impact scores were analysed in relation to population variables.

Reporting for specific materials, systems and methods

We require information from authors about some types of materials, experimental systems and methods used in many studies. Here, indicate whether each material, system or method listed is relevant to your study. If you are not sure if a list item applies to your research, read the appropriate section before selecting a response.

Materials & experimental systems

n/a	Involved in the study	
☒	☐	Antibodies
☒	☐	Eukaryotic cell lines
☒	☐	Palaeontology and archaeology
☒	☐	Animals and other organisms
☐	☒	Human research participants
☒	☐	Clinical data
☒	☐	Dual use research of concern

Methods

n/a	Involved in the study	
☒	☐	ChIP-seq
☒	☐	Flow cytometry
☒	☐	MRI-based neuroimaging

Human research participants

Policy information about studies involving human research participants

Population characteristics	see above
Recruitment	To maximise visibility and inclusiveness articles describing the study were placed on the BBC2 Horizon, BBC homepage, BBC

Recruitment	News homepage and circulated on mobile news meta-apps from January 1st, 202052. To maximise representativeness of the sample there were no inclusion/exclusion criteria. Due to this high visibility and accessibility of the software, our resultant sample is more inclusive than studies that use randomised sampling methods within preexisting databases or through telephone or letter recruitment.
Ethics oversight	The Imperial College Research Ethics Committee (17IC4009)

Note that full information on the approval of the study protocol must also be provided in the manuscript.
